# Mendelian randomization identifies blood metabolites previously linked to midlife cognition as causal candidates in Alzheimer’s disease

**DOI:** 10.1073/pnas.2009808118

**Published:** 2021-04-16

**Authors:** Jodie Lord, Bradley Jermy, Rebecca Green, Andrew Wong, Jin Xu, Cristina Legido-Quigley, Richard Dobson, Marcus Richards, Petroula Proitsi

**Affiliations:** ^a^Department of Basic and Clinical Neuroscience, Maurice Wohl Clinical Neuroscience Institute, Institute of Psychiatry, Psychology & Neuroscience, King’s College London, London, SE5 5AF, United Kingdom;; ^b^Social, Genetic and Developmental Psychiatry Centre, Institute of Psychiatry, Psychology & Neuroscience, King’s College London, London, SE5 8AF, United Kingdom;; ^c^National Institute for Health Research Maudsley Biomedical Research Centre, South London and Maudsley National Health Service (NHS) Foundation Trust, London, SE5 8AF, United Kingdom;; ^d^MRC Unit for Lifelong Health and Ageing at UCL, University College London, London, WC1E 7HB, United Kingdom;; ^e^Institute of Pharmaceutical Science, King’s College London, London, SE1 9NH, United Kingdom;; ^f^Systems Medicine, Steno Diabetes Centre Copenhagen, 2820 Gentofte, Denmark;; ^g^Department of Biostatistics and Health Informatics, Institute of Psychiatry, Psychology and Neuroscience, King’s College London, London, SE5 8AF, United Kingdom;; ^h^National Institute for Health Research Biomedical Research at South London and Maudsley NHS Foundation Trust and King’s College London, London, SE5 8AF, United Kingdom;; ^i^Health Data Research UK London, University College London, London, NW1 2DA, United Kingdom;; ^j^Institute of Health Informatics, University College London, London, NW1 2DA, United Kingdom;; ^k^National Institute for Health Research Biomedical Research Centre at University College London Hospitals NHS Foundation Trust, London, NW1 2DA, United Kingdom

**Keywords:** Alzheimer’s disease, metabolomics, causality, biomarkers, Mendelian randomization

## Abstract

There are currently no disease-modifying treatments for Alzheimer’s disease (AD), and an understanding of preclinical causal biomarkers to help target disease pathogenesis in the earliest phases remains elusive. Here, we investigated whether 19 metabolites previously associated with midlife cognition—a preclinical predictor of AD—translate to later clinical risk, using Mendelian randomization (MR) to tease out AD-specific causal relationships. Summary statistics from the largest genome-wide association studies (GWASs) for AD and metabolites were used to perform bidirectional univariable MR. Bayesian model averaging (BMA) was additionally performed to address high correlation between metabolites and identify metabolite combinations that may be on the AD causal pathway. Univariable MR indicated four extra-large high-density lipoproteins (XL.HDL) on the causal pathway to AD: free cholesterol (XL.HDL.FC: 95% CI = 0.78 to 0.94), total lipids (XL.HDL.L: 95% CI = 0.80 to 0.97), phospholipids (XL.HDL.PL: 95% CI = 0.81 to 0.97), and concentration of XL.HDL particles (95% CI = 0.79 to 0.96), significant at an adjusted *P* < 0.009. MR–BMA corroborated XL.HDL.FC to be among the top three causal metabolites, in addition to total cholesterol in XL.HDL (XL.HDL.C) and glycoprotein acetyls (GP). Both XL.HDL.C and GP demonstrated suggestive univariable evidence of causality (*P* < 0.05), and GP successfully replicated within an independent dataset. This study offers insight into the causal relationship between metabolites demonstrating association with midlife cognition and AD. It highlights GP in addition to several XL.HDLs—particularly XL.HDL.FC—as causal candidates warranting further investigation. As AD pathology is thought to develop decades prior to symptom onset, expanding on these findings could inform risk reduction strategies.

## Significance

The absence of disease-modifying therapeutics for Alzheimer’s disease (AD) continues, and an understanding of early, easily accessible biomarkers to inform treatment strategies remains elusive. This study uses knowledge of blood metabolites previously associated with midlife cognition—a preclinical predictor of AD—to systematically investigate causal associations with later AD status. Given that the pathological changes underlying AD are thought to develop years before clinical manifestations of the disease, developing these findings further could hold special utility in informing early treatment intervention.

More than 50 million people worldwide currently live with dementia, and with an aging world population, this figure is expected to increase to more than 152 million by 2050 (World Alzheimer Report 2018). The most common dementia type is Alzheimer’s disease (AD), characterized by impaired everyday function, severe cognitive decline—particularly working, episodic, and declarative memory (1)—and a range of neuropsychiatric symptoms (2). It represents a major source of global morbidity and mortality and poses significant human and economic costs (3).

Disappointingly, AD drug development has proven difficult, with a 99.6% failure rate in the decade of 2002 to 2012, and this rate continues at the same low level today (4). Numerous reasons have been proposed as to why such clinical trials have failed, including incomplete understanding of true causal mechanisms and a failure to intervene early enough in the pathological cascade. It is therefore necessary to discover biomarkers that can identify individuals at high risk of developing AD and at the earliest possible stages of pathology onset. Moreover, it is important for these to be potentially modifiable so as to offer targets for preventative or therapeutic strategies.

Metabolomics represents one avenue that may give a deeper insight into AD etiology. Metabolites are small molecules (<1,500 atomic mass units) with a role in metabolism (5). As the products of many biological processes, they sit at the end of the systems biology pathway and therefore represent effective intermediate phenotypes to a given disease because of their proximity to the clinical endpoint (6, 7). Due to 1) their noninvasive nature of measurement, 2) the fact that they are potentially modifiable through diet and lifestyle, and 3) the ability of many to cross the blood brain barrier, blood metabolites are both practical and valuable markers of biological processes and disease states in dementia (8).

Markers of lipid metabolism have received particular attention in this context, as the impairment of lipid metabolism has been associated with AD (5, 8–11) and beta-amyloid (Aβ) burden (12, 13). Relevant to early intervention, they have also been associated with cognitive performance and brain function during normal aging (14, 15). Recently, using a large British population-based birth cohort, we investigated associations between 233 blood metabolites and both memory and processing speed at 60 to 64 y of age as well as changes in these cognitive domains from 60 to 64 to 69 y old. Associations with several metabolite classes were observed, including fatty acids (FAs), various compositions of high-density lipoproteins (HDLs), and glycoprotein acetyls (GP) (16).

However, it is not yet established whether these metabolites are causally associated with dementia and AD. Using knowledge from these preclinical associations to investigate translatability to later AD risk could hold special utility in informing early treatment intervention, particularly if a causal relationship can be shown. This study therefore aims to expand our observational findings and assess whether 19 blood metabolites previously associated with late midlife cognition causally associate with later clinical AD status. Both univariable and Bayesian multivariable Mendelian randomization (MR) approaches are harnessed to interrogate independent as well as group associations, and a range of sensitivity analyses are performed to further scrutinize results. Identifying candidate blood metabolites, which are detectable preclinically and on the causal pathway to later AD diagnosis, will aid in facilitating further research into early intervention strategies and more targeted therapeutics.

## Results

### Metabolite Selection.

Metabolite data were obtained from summary statistics of the latest and largest metabolite genome-wide association study (GWAS), which investigated the genetic component of 123 blood metabolites on nearly 25,000 individuals (17) (data: computationalmedicine.fi/data#NMR_GWAS). Of the 123 metabolites available for analysis, selection was based on our previously published observational study, which investigated associations between blood metabolites and lifetime cognition using data from the Medical Research Council National Survey of Health and Development (1946 British birth cohort) (18). Briefly, this study measured the association between three domains of cognition [short-term memory, delayed verbal memory, and processing speed (19)] and levels of 233 blood metabolites in 798 participants aged 60 to 64 y old (18, 20) and then again at age 69 y (*n* = 633) (18). A total of 20 metabolites were significantly associated with at least one measure of midlife cognition in our observational study, and 19 of these were causally investigated within the present study (for further information, see *Methods*).

### Primary Analyses.

#### Bidirectional univariable MR.

**Fig. 1.**
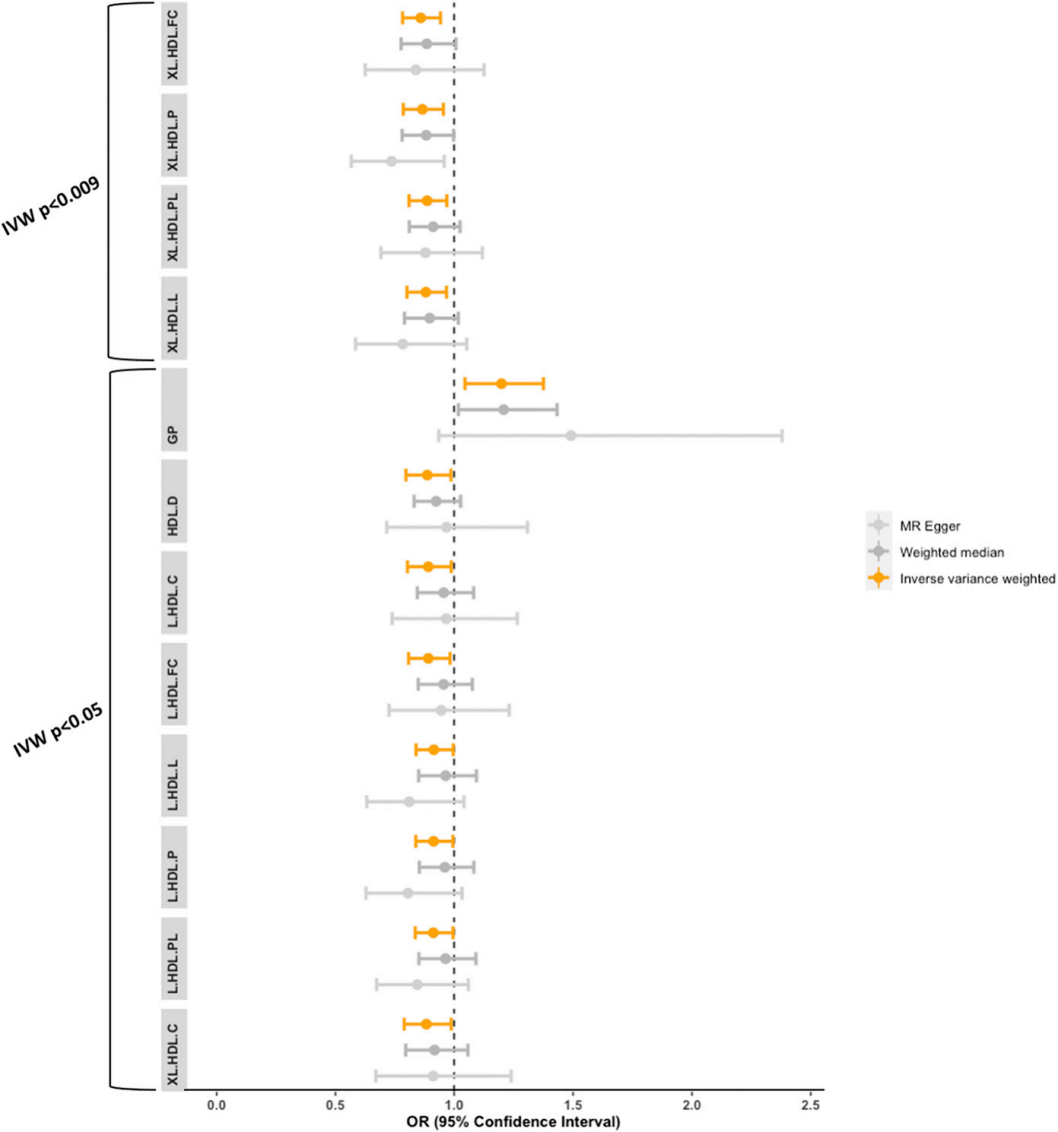
Association of metabolites associated with AD at *P* < 0.05 in primary univariable analyses. Standardized odds ratio (μ = 0, SD = 1) and 95% CI error bars for IVW, MR–Egger, and weighted median estimates (*n* = 12). Orange bars represent estimates from primary univariable analyses. Gray bars represent conservative estimates from MR–Egger and weighted median sensitivity analyses. Sensitivity estimates appear in gray to indicate lower precision of these estimates relative to primary analyses, resulting in larger windows of uncertainty. HDL = high-density lipoproteins, XL.HDL = very large high-density lipoproteins, L.HDL = large high-density lipoproteins, FC = free cholesterol, P = concentration of particles, PL = phospholipids, L = total lipids, C = total cholesterol, D = mean diameter, and GP = glycoprotein acetyls.

Using metabolite data from Kettunen et al. (17) together with clinically diagnosed AD data from Kunkle et al. (21), a series of two-sample univariable inverse-variance weighted (IVW) MR analyses were conducted to investigate the bidirectional causal relationship between each of the selected metabolites and AD. For strong evidence of causality, estimates were required to demonstrate association below an adjusted significance threshold of *P* < 0.009 (*SI Appendix*, Info. S3). By this criterion, four metabolites retained strong evidence of an inverse causal association with AD: free cholesterol in very large HDLs (XL.HDL.FC) [Odds Ratio (OR) = 0.86, 95% CI = 0.78 to 0.94, *P* = 0.001], total lipids in very large HDLs (XL.HDL.L) (OR = 0.88, 95% CI = 0.80 to 0.97, *P* = 0.008), phospholipids in very large HDLs (XL.HDL.PL) (OR = 0.89, 95% CI = 0.81 to 0.97, *P* = 0.008), and concentration of very large HDL particles (XL.HDL.P) (OR = 0.87, 95% CI = 0.79 to 0.96, *P* = 0.004). GP also demonstrated evidence of suggestive causal association, with IVW estimates indicating increased odds of AD given higher GP levels (OR = 1.20, 95% CI = 1.05 to 1.38), and both HDL.D and XL.HDL.C demonstrated nominally significant associations in the negative direction (HDL.D: OR = 0.89, 95% CI = 0.80 to 0.99, XL.HDL.C: OR = 0.88, 95% CI = 0.79 to 0.99), though *P* values did not reach adjusted significance (*P* > 0.009) (Dataset S1, Fig. 1, and *SI Appendix*, Fig. S1 *A*–*S*).

For seven large and one small HDL (L.HDLs and S.HDL, respectively) (Dataset S2), single nucleotide polymorphism (SNP) instrumental variables (IVs) within the ApoE genomic region were removed prior to analyses due to known violations to core MR assumptions (see *Methods*). The predicted causal effect for each of the L.HDLs on clinical AD using non-ApoE–related IVs were in the negative direction with a similar magnitude of effect across point estimates (OR range: 0.89 to 0.91). 95% CIs remained in the negative direction for all seven L.HDLs (Fig. 1 and Dataset S1), though only nominal significance was reached (*P* < 0.05) (Dataset S1) and not for S.HDL.TG. No other metabolites were found to be genetically predicted by ApoE.

When exposure and outcome were reversed to investigate the potential for reverse causation, there was no evidence of a causal relationship in the opposite direction, from AD to metabolite. Using 24 independent SNP IVs, excluding those within the ApoE genomic region, significance did not exceed *P* < 0.1 (Dataset S3 and *SI Appendix*, Figs. S2 and S3*S*).

#### Bayesian model averaging MR.

Metabolites demonstrate notable correlation both phenotypically (22) and genetically (Dataset S4). Consequently, a high degree of instrumental variable overlap is identifiable across metabolites in univariable analyses (Dataset S5). Univariable approaches, while useful for identifying individual causal associations, assume exposures to be independent and therefore 1) neglect instances in which “group” relationships may exist and 2) do not allow for the effect of interrelated exposures to be disentangled by way of removing nonindependent signal. Bayesian model averaging MR (MR–BMA) offers an alternative approach which allows multiple metabolites to be modeled together. In this way, subgroups of metabolites which may act together on the causal pathway to AD may be identified, and independent metabolites can be appropriately ranked according to their independent causal signal. Thus, this method allows related metabolites to be disentangled to identify which may be driving the true causal signal over others. Like conventional multivariable MR, the inclusion of multiple exposures with overlapping instruments allows for “measured pleiotropy” to be sufficiently handled (22). Unlike conventional multivariable MR (23), however, this method also scales particularly well to high-throughput and highly correlated data (22).

Following the pruning of metabolites with genetic correlations >95% (including the removal of univariably significant XL.HDL.L, XL.HDL.PL, and XL.HDL.P), nine metabolites were jointly analyzed (see *Methods* and Dataset S4). Results of single-metabolite causal rankings in accordance with their marginal posterior probability (MIP) are presented in Table 1. As this is a Bayesian method, frequentist *P* values are unavailable. Instead, inferences can be made on the basis of posterior probabilities and ranking performance. Those ranked with the highest MIP are indicative of being the strongest “true causal” candidates over those of lower rank. Table 1 also confirms corresponding model average causal effect (MACE) estimates, reflecting the average direct effect of each metabolite on AD, independent of contributary signal from any other metabolites included within the model. It is worth noting that the purpose of MR–BMA is to correctly detect (by way of ranking) true causal risk factors rather than to unbiasedly estimate the magnitude of the direct causal effect, as these will be biased toward the null due to shrinkage applied in variable selection (22). MACE can be used, however, to gain insight into the direction of effect and magnitude relative to other metabolites included within the model. GP was estimated as the highest ranked causal metabolite (MIP = 0.465, MACE = 0.09), followed by three XL.HDL particles (XL.HDL.C: MIP = 0.179, MACE = −0.02; XL.HDL.FC: MIP = 0.178, MACE = −0.02; and XL.HDL.CE MIP = 0.164, MACE = −0.02). When whole models with variations of metabolite combinations were assessed, these same four metabolites were present within the four highest ranked causal models, with model-based posterior probabilities (pps) of 0.287, 0.113, 0.112, and 0.102 for GP, XL.HDL.C, XL.HDL.FC, and XL.HDL.CE, respectively (Table 2).

**Table 1.** Metabolites ranked by their MIP and MACE in MR–BMA analyses

Metabolite	MIP	MACE
GP	0.465	0.088
XL-HDL-C	0.179	−0.022
XL-HDL-FC	0.178	−0.022
XL-HDL-CE	0.164	−0.017
S-HDL-TG	0.107	−0.015
L-HDL-C	0.098	−0.007
L-HDL-CE	0.096	−0.007
DHA	0.044	−0.003
PUFA	0.024	0.001

**Table 2.** Top nine causal models based on whole-model posterior probabilities estimated within MR–BMA analyses

Exposure combinations	Posterior probability
GP	0.287
XL-HDL-C	0.113
XL-HDL-FC	0.112
XL-HDL-CE	0.102
L-HDL-C	0.050
L-HDL-CE	0.049
Gp,XL-HDL-C	0.020
XL-HDL-CE,Gp	0.019
Gp,S-HDL-TG	0.019

### Sensitivity Analyses.

#### Univariable MR.

When causal relationships were re-estimated using MR–Egger and weighted median (conservative methods which are sensitive to pleiotropy and instrument invalidity), directionality of results were in agreement with all nominally significance metabolite exposures (*P* < 0.05) from primary analyses. CIs were, however, wider, resulting in a number of estimates crossing the null (Fig. 1). The intercept from MR–Egger estimates demonstrated no evidence of horizontal pleiotropy (Dataset S1). Funnel plots also demonstrated symmetrical distribution of SNP effects around the effect estimate for most tests, suggesting balanced pleiotropy, although this was not the case for metabolites with small SNP *n* (*SI Appendix*, Fig. S4 *A*–*S*). MR–pleiotropy residual sum and outlier (PRESSO)—a method for detecting and correcting for outliers within the data—demonstrated attenuated *P* values for all four metabolites which were strongly associated in primary analyses (*P* < 0.009: XL.HDL.FC, XL.HDL.L, XL.HDL.P, and XL.HDL.PL). Significance at the 5% level was, however, retained, and no significant outliers were detected (Dataset S1). The leave-one-out method, on the other hand, indicated two influential SNPs (rs1532085 and rs261291) for most HDL subfractions, and one influential SNP was also found for GP (rs77303550) (*SI Appendix*, Fig. S5 *A*–*S*). Removal of these SNPs resulted in wider CIs, with only XL.HDL.FC retaining significance at *P* < 0.05. Leave-one-out analyses, when AD was set as the exposure, indicated no notable outliers (*SI Appendix*, Fig. S6 *A*–*S*). MR–PRESSO, on the other hand, did detect outliers, but the corrected *P* value upon removal of these remained in agreement with primary tests (Dataset S3). As an additional sensitivity analysis, noninferable palindromic SNP instruments were dropped from analyses, and MR estimates were recomputed. This resulted in almost identical results across IVW, MR–Egger, and weighted median results (Dataset S6).

#### MR*–*BMA.

Sensitivity analyses consisted of 1) Q-statistic computation to identify heterogeneous/outlier instruments and 2) Cook’s distance (Cd) to identify influential points within the top models identified. Q-statistics indicated no deviant instruments (all Q < 10. *SI Appendix*, Fig. S7 *A*–*D*). The genetic variant with the largest Cd was rs1532085, near the *LIPC* gene (*SI Appendix*, Figs. S8 *A*–*C* and S9*A*). This had a Cd > 0.19 in all three XL-HDL models (XL.HDL.C: Cd = 1.095, XL.HDL.FC: Cd = 1.25, and XL.HDL.CE: Cd = 1.168). rs2575876 on the *ABCA1* gene (*SI Appendix*, Fig. S8 *A*–*C* and Fig. S9*B*) also demonstrated a high Cd in all three XL-HDL models (XL.HDL.C: Cd = 0.392, XL.HDL.FC: Cd = 0.247, and XL.HDL.CE: Cd = 0.302), and variant rs247617, near the *CETP* gene (*SI Appendix*, Figs. S8 *A* and *B* and S9*C*), also had high Cd in XL.HDL.C (Cd = 0.229) and XL.HDL.FC (Cd = 0.265). Finally, variant rs77303550 on the *TXNL4B* gene (*SI Appendix*, Figs. S8*D* and S9*D*) had a high Cd in the GP model (Cd = 0.518), though was <0.19 in all other models (*SI Appendix*, Fig. S8 *A*–*C*). A full overview of Q-statistics and Cds for the top four MR–BMA models are presented in Dataset S7. Removal of influential points reduced MIPs, particularly for HDLs, but did not substantially change results (Datasets S8 and S9). All MR–BMA results remained consistent when reran with noninferable palindromic SNPs removed (Dataset S10).

### Post Hoc Exploratory Analyses.

#### Linkage Disequilibrium (LD) overlap between influential points and AD.

Two core assumptions of MR are 1) the “exchangeability assumption”—that is, that the effect of an IV on the outcome does not occur because of confounding—and 2) the “exclusion restriction assumption,” which assumes that the association between an IV and outcome occurs only via the exposure of interest (23). Within the primary scope (*SI Appendix*, Info. S1), any IVs associated with the outcome at genome-wide significance were removed due to potential violations to either of these assumptions. However, violations may also occur if the IVs utilized represent the same locus as genes known to significantly associate with the outcome. To explore this further, we visually inspected Linkage Disequilibrium (LD) regions of each influential point and cross-checked whether any of these spanned gene regions were previously shown to associate with AD, using information from Kunkle et al. (21). Locus zoom plots are presented within *SI Appendix*, Fig. S9 *A*–*D*), and confirmation of Kunkle lead SNPs and related genomic regions are presented in Dataset S11. No overlap was observed between any of our influential point regions and genomic regions identified as being associated with AD SNPs. Influential point rs1532085 was, however, observed to be located within the LIPC gene, which is located <50 kb from ADAM10—a gene associated with the lead rs593742 SNP from Kunkle et al. (21). To inspect this further, an additional visualization was produced for rs593742 using data from Kunkle et al. (21) (*SI Appendix*, Fig. S9*E*). While rs593742 was found to overlap with the LIPC region, no evidence of LD between the HDL-related rs1532085 SNP nor the AD-related rs593742 specifically was observed, and there was no evidence of overlap between rs1532085 LD SNPs and ADAM10—indicating independence of this region (https://www.ncbi.nlm.nih.gov/gene).

#### One-sample univariable MR.

To further interrogate the validity of findings from MR analyses, baseline individual level data from the Alzheimer’s Disease Neuroimaging Initiative (ADNI) (24) were utilized to perform a small-scale replication using the two-stage least squares (2SLS) methodology. Here, we obtained NMR metabolite data for those metabolites demonstrating adjusted significance within primary univariable analyses (XL.HDL.FC, XL.HDL.L, XL.HDL.PL, and XL.HDL.P) (*n* = 878) and for the highest ranked causal metabolite identified by MR–BMA (GP) (*n* = 894). An adjusted significance threshold of *P* < 0.02—representing 2.45 independent tests, accounting for correlation structures among metabolites (*SI Appendix*, Info. S3)—was expected to demonstrate strong evidence of causality. In line with this criterion, GP was the only metabolite to successfully replicate at the adjusted level (*P* = 0.004). Directionality was in agreement with primary analyses, with an effect size of greater magnitude but a larger window of uncertainty (OR = 2.28, 95% CI = 1.3 to 4.0). No other metabolite reached adjusted significance. However, weighted F-statistics for each metabolite ranged from 5.85 to 8.55, indicating low instrument strength to detect causal estimates (Dataset S12).

## Discussion

The absence of disease-modifying therapeutics for AD continues, and an understanding of early, easily accessible biomarkers to inform treatment strategies remains sparse. Using knowledge of associations between preclinical risk factors and potential biomarkers and assessing how well such markers translate through to later clinical risk could therefore hold special utility in informing early treatment intervention, particularly if a causal relationship can be shown. This study uses blood metabolites previously associated with midlife cognition to systematically investigate causal associations with later AD status. Using summary data from the largest metabolomics and AD GWASs to date, causality was interrogated using a combination of both bidirectional univariable and MR–BMA, with results further scrutinized using a range of sensitivity and post hoc measures. Primary analyses indicated an inverse causal relationship between subfractions of extra-large HDL molecules—particularly XL.HDL.FC—and AD, indicating a protective effect. GP on the other hand, when modeled with consideration of other metabolites, demonstrated evidence of a direct casual effect in the positive direction, indicating that this metabolite may contribute to increased AD risk. GP’s risk-increasing effect was further supported in an independent small-scale replication using individual level data.

Within the medical literature, higher levels of HDLs are commonly referred to as being health promoting, demonstrating vascular protective properties and a consistent association with lowered cardiovascular and stroke risk (25–29). In line with this health-promoting hypothesis, our primary analyses found evidence for a causally protective effect of XL.HDLs on clinical AD diagnosis. Of these, free cholesterol in extra-large HDLs (XL.HDL.FC) demonstrated particular pertinence, representing the strongest univariable relationship with AD and showing the greatest consistency across both univariable and Bayesian methods. Three addition XL.HDLs (XL.HDL.P, XL.HDL.PL, and XL.HDL.L) demonstrated evidence of a protective effect in univariable analyses, significant at *P* < 0.009. These were, however, excluded from MR–BMA due to a genetic correlation >95% with other HDLs. This nonindependence of genetic signal could indicate that the univariable causal effect of these three metabolites captures signal across the HDL metabolite family as opposed to demonstrating specificity for the individual subfractions themselves. The benefit of MR–BMA is that it is able to disentangle these intertwined effects, and indeed, while XL.HDL-P, PL, and L were removed from BMA models, XL.HDLs remained implicated, with both XL.HDL-FC and -C ranking within the top three independent causal metabolites and effects remaining in the protective direction. Our exploratory post hoc analyses, on the other hand, failed to replicate XL.HDL associations. However, small sample (*n* < 900) and weak instrumental strength (F-statistics < 10) imply that this may simply reflect a lack of power in our replication cohort.

Evidence of a protective effect also extended to a number of large HDLs in univariable analyses. Though these did not reach adjusted significance, they demonstrated consistent negative directionality in both primary and sensitivity analyses and retained significance at the 5% level for IVW estimates. The protective effect observed for HDLs corroborate our previous observational study, which demonstrated positive associations of HDLs and midlife cognition, indicative of potential neurocognitive protective properties. HDLs have also been implicated more widely in age-related cognitive decline and dementia (30) with evidence from human studies, animal models, and bioengineered arteries of a cerebrovascular protective effect, which commonly show dysfunction in AD (31). Results are also supported by existing AD GWAS, with SNP associations found near genes encoding HDL protein components and biogenesis proteins such as *APOE*, *ABCA1*, *APOA1* and *2*, *CLU*, *LCAT*, and *CETPI* (31). Previous MR studies, including ours (32, 33), have failed, however, to show a causal link between HDL levels and AD. This is potentially due to insufficiently capturing HDL composition complexity. This study provides deeper granularity through inclusion of specific subfractions and sizes of HDL and accounts for the interrelated structure of such subfractions through use of Bayesian multivariable methodology.

GP—a marker of inflammation—demonstrated a causal association in the positive direction, both in univariable analyses and MR–BMA. As with large HDLs, univariable results remained significant at the *P* < 0.05 level only. However, when direct effects were measured using MR–BMA—accounting for interrelation among metabolites—GP was estimated to have the largest causal effect of all metabolites within the model and demonstrated the highest posterior probability of existing within the true causal model. Furthermore, GP was the only metabolite to successfully replicate within a small-scale independent cohort, though instrument power was low (F < 10). This risk-increasing relationship aligns with our previous study (16), which observed an association between GP and lower cognitive ability in late midlife, consistent with findings from a large independent cohort (14). Additionally, alpha-1-acid glycoprotein has been shown to be a strong predictor of 10 y mortality (34) as well as all-cause mortality in a recent large meta-analysis of >40K individuals (35). Changes in the level of several glycoproteins have also been observed in the hippocampus and inferior parietal lobe in human AD (36). Some of these glycoproteins interact with neurofibrillary tangles, leading to speculation that changes in their glycosylation may be associated with the pathogenesis of this disease (36).

Interestingly, while our previous observational study found the strongest associations to be between FAs and late midlife cognition, the present study found no evidence for causal associations between these and AD. This may in part be due to only a low number of instruments available for FAs (five SNPs available for both omega-3 and DHA, and six available for monounsaturated FAs), resulting in a lack of statistical power to detect a causal relationship between these metabolites and AD. Alternatively, this inconsistency could be attributable to the different outcome phenotypes (cognition verses AD), with FAs potentially being associated with non-AD–related cognitive decline but not AD specifically. Finally, observed associations between FAs and cognition may simply reflect confounding, highlighting the importance of methods such as MR for disentangling such scenarios. Future research on larger, independent samples will be an important endeavor to better understand the discrepant findings observed here.

Strengths of this study include the use of the largest and most up to date GWASs available for both NMR metabolomics and AD. Being the first of its kind to utilize knowledge from preclinical associations between metabolites and midlife cognition also allows a window of insight into causally relevant metabolites, which may hold utility preclinically. Moreover, through use of bidirectional MR, relationships were interrogated in both directions as opposed to relying on a priori (potentially erroneous) assumptions about directionality. Employment of MR–BMA also allowed for correlations between metabolites to be accounted for and for multivariable models of combined metabolites to be proposed. Furthermore, the inclusion of sensitivity analyses across univariable and multivariable models allowed for further interrogation of MR assumptions, ensuring that any notable changes in results could be investigated. This was further extended through the addition of a small-scale post hoc replication using independent, individual level data.

There remain, however, some limitations. First, power. For several metabolites, less than 10 genetic variants were available at genome-wide significance, with two having only five variants available at this level. While steps were taken to ensure individual SNPs did not suffer from weak instrument bias through calculation of per-instrument F-statistics, we cannot exclude the possibility of false negative errors because of insufficient statistical power. Power was also a notable drawback within replication analyses, with a sample *n* of up to 894 in comparison to ∼25,000 and ∼95,000 for metabolite and AD summary data, respectively, in a priori analyses. This was reflected in instrument strength, with no metabolite reaching an F-statistic >10. While replication proceeded as an exploratory step with the view that internal validation, when possible, is important to assess the consistency of findings, such post hoc results should be considered with caution until further replications of greater sample size can be considered. Second, due to the absence of available stratified GWA data, the present study was unable to stratify on key variables such as sex—something which our previous observational study indicated may modify many metabolite–cognition associations and may plausibly also modify metabolite–AD associations (16).

A third limitation lies with exclusion of ApoE-related instrumental variables. This was necessary due to known associations between ApoE and non-AD traits, such as coronary artery disease (37), violating the MR exchangeability assumption. However, as ApoE is directly implicated in the production of lipoproteins and lipid metabolism (38), its removal likely attenuated observed causal associations. This is of particular relevance to large HDLs given that, for those models where ApoE instruments were removed, evidence of a negative causal relationship was observed at the nominal level but failed to reach adjusted significance. It remains plausible—particularly given the opposing direction of ApoE-related effect sizes between HDLs and AD, equating to a negative association (Dataset S13)—that this reflects attenuated power which would otherwise have been recovered with the addition of ApoE instruments. Finally, while several IVW causal associations were observed, sensitivity analyses revealed a number of influential points and wider CIs, resulting in a loss of significance. Influential points may arise for a number of reasons, one of which being due to violations of MR exchangeability and exclusion restriction assumptions. While instrument validity can never be concluded with certainty, steps were taken to mitigate violations, such as the removal of instruments with known pleiotropy and exclusion of SNPs demonstrating genome-wide significance with the outcome of interest. Moreover, post hoc visual analyses indicated no LD between influential points within this study and gene regions associated with lead AD SNPs from the latest GWAS conducted by Kunkle and colleagues (21). Together, these add weight to assumptions of instrument validity. Both MR–Egger and weighted median were introduced as a means for re-estimating causal estimates in the presence of potential pleiotropy. Failure of these to detect a causal effect could therefore indicate violation to MR assumptions. Robust method estimates do, however, have greater imprecision than that of IVW estimates. As such, they commonly present with larger windows of uncertainty and lower power to detect causal estimates (39). MR–Egger also provides a test of pleiotropy via its intercept, and this indicated no significant pleiotropy across any of our IVW estimates. Moreover, no significant heterogeneity was observed, and consistent directionality for point estimates were maintained across different univariable methodologies. Additionally, MR–BMA—a method able to account for measured pleiotropy—largely corroborated univariable findings, ranking XL.HDLs and GP as the most likely causal metabolites of those included. Taken together, the weight of evidence supports IVW conclusions, with no indication that core model assumptions have been violated. Instead, a loss of significance in sensitivity measures are likely a reflection of higher imprecision and low statistical power.

As the pathological changes underpinning AD are thought to develop at least a decade prior to the onset of symptoms, it is important to identify modifiable targets for intervention at an early stage, before AD pathology has caused major irreversible damage. This study utilizes knowledge of preclinical associations between metabolites and midlife cognition to investigate causal associations between early candidate biomarkers and later AD risk. Our findings highlight GP as a particularly promising risk-increasing metabolite, and XL.HDLs—particularly XL.HDL.FC—warrant further follow-up as protective candidates on the AD causal pathway. Progressing these findings could hold special value in informing future risk reduction strategies.

## Methods

**Fig. 2.**
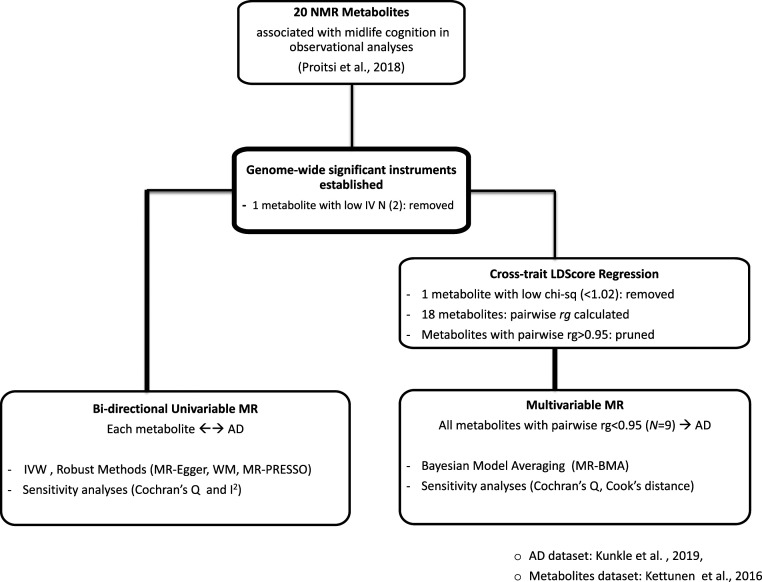
Study design. Flowchart describing sequence of analytical steps in line with core study scope.

A flow diagram summarizing the methodology is detailed in Fig. 2. A document containing further details on motivation and scope in line with MR-reporting guidelines outlined by Burgess et al. (39) is provided in *SI Appendix*, Info. S1.

### Data Sources.

Summary statistics from the latest and largest metabolite GWAS were used for all MR analyses (17) (data: computationalmedicine.fi/data#NMR_GWAS). This GWAS investigated the genetic component of 123 blood metabolites on nearly 25,000 individuals using NMR spectroscopy. This platform provides a detailed characterization of metabolite measures and ratios representing a broad molecular signature of systemic metabolism. Multiple metabolic pathways were covered, including the following: lipoprotein lipids and lipid subclasses, FAs and FA compositions, and amino acids and glycolysis precursors. Specific details are described elsewhere (40–42).

Of the 20 metabolites previously associated with cognition, all had at least one SNP association at genome-wide significance (GWS) (*P* < 5 × 10^−8^). However, as only two GWS SNPs were available for pyruvate, this metabolite was removed due to power concerns, leaving 19 metabolites for MR. To avoid weak instrument bias, a computed F-statistic of at least 10 was also required for all SNP instruments.

For AD, summary statistics from the latest GWAS of clinically diagnosed late onset AD by Kunkle and colleagues were utilized (21). This study consisted of three stages: 1) a discovery phase of 63,926 samples, 2) a replication phase of 18,845 samples, and 3) a post-replication phase of 11,666 samples. For MR with AD as an outcome, stage one summary data were utilized, and for MR with AD as an exposure, stages one and two data were employed.

A proportion of data used in preparation of this article was obtained from the ADNI database (http://adni.loni.usc.edu/). As such, the investigators within ADNI contributed to the design and implementation of ADNI and/or provided data but did not participate in the analysis or writing of this report. A complete listing of ADNI investigators can be found at http://adni.loni.ucla.edu/research/active-investigators/. Data used in preparation for a proportion of this article were also generated by the Alzheimer's Disease Metabolomics Consortium (ADMC). As such, the investigators within the ADMC provided data but did not participate in the analysis or writing of this report. A complete listing of ADMC investigators can be found at https://sites.duke.edu/adnimetab/team/.

### MR.

#### Univariable analyses investigating metabolites as causal risk factors for AD.

##### SNP selection.

All data extraction, preprocessing, and analyses were performed within R.3.6.1. using the MR-Base package (v.0.4.25) (43). SNP instruments selected for each metabolite were those available within the metabolomic quantitative trait loci (mQTL) catalog within MR-Base. All mQTLs available within this catalog were precurated using the data from Kettunen et al. (17), and only independent instruments were made available for selection. For each metabolite, summary statistics consisting of effect sizes, SEs, and *P* values for all GWS SNPs were extracted from each of the GWAS datasets (17). SNPs associated with AD at GWS were excluded due to potential violation of the MR exchangeability assumption (39), which assumes SNP instruments are not associated with confounding risk factors. Any SNPs within the ApoE genomic region (chromosome 19, base pairs 4,500,000 to 4,580,000) were also excluded for this reason, as ApoE is an established risk factor for traits additional to AD, such as coronary artery disease (37). This resulted in SNP exclusions from large HDL subclasses only (Datasets S2 and S13). Data were harmonized between AD and metabolite datasets, and SNPs with Minor Allel Frequency (MAF) < 0.01 were excluded. All GWAS were assumed to be coded on the forward strand, and thus, no palindromic SNPs were excluded from analyses. However, additional sensitivity analyses were performed excluding noninferable palindromic SNPs (MAF > 0.40), with metabolite MAFs used to infer AD allele frequencies because of MAF nonavailability within the AD dataset.

##### Primary analyses.

Total causal estimates were computed using IVW two-sample MR, setting each metabolite as the exposure in turn and AD as the outcome. Briefly, IVW–MR uses a univariable model to regress SNP instrument associations with an outcome on SNP instrument associations with an exposure weighted by the inverse of the variance in SNP outcome associations (44). To reflect MR’s exclusion restriction assumption, which states that SNP instrument(s) must only be associated with the outcome via the exposure (44), the IVW intercept is constrained to zero. Results are presented in OR per 1 SD unit to enable a comparison of the magnitude of effect across all exposures.

##### Sensitivity analyses.

Two robust methods—MR–Egger and weighted median—were utilized to re-estimate casual associations with IVW assumptions relaxed. Briefly, MR–Egger re-estimates IVW causal estimates while removing the intercept constraint. Large deviations from zero are taken as evidence of violation to MR’s exclusion restriction and exchangeability assumptions (45), and large discrepancies between Egger and IVW estimates are indicative of pleiotropy. Weighted median provided an alternative estimate which remains valid provided 50% of instruments are valid (46). Briefly, causal estimates for each instrument are ordered and weighted by their association strength. The final estimate is then taken as the 50th weighted percentile of the ordered estimate. Influential points were investigated using leave-one-out analyses, and Cochran’s Q was calculated to test for heterogeneity among instruments (Q—*P* < 0.05 indicating significant heterogeneity). An MR–PRESSO test was further utilized to identify and correct for potential bias in estimates due to pleiotropy (47). Briefly, this test consists of up to three parts, with 1) the “global test” providing an estimate for the degree of horizontal pleiotropy (significant pleiotropy indicated by *P* < 0.05), 2) the “outlier corrected causal estimate” providing a corrected estimate for any significant pleiotropy detected, and 3) the “distortion test” providing an estimate for the degree to which the original and corrected estimates differ (*P* < 0.05 indicating a significant difference following corrections for pleiotropy). Tests two and three are implemented only in cases where *P* < 0.05 for global test estimates.

#### Univariable analyses investigating AD as a causal risk factor for metabolite levels.

To explore causality in the opposite direction, AD was set as the exposure with each metabolite in turn set as the outcome. The same analysis pipeline was followed as above, testing the association of GWS SNPs from stages one and two of Kunkle et al. (21). Following clumping (using an R^2^ threshold of 0.001) and the removal of ApoE SNPs or those with MAF < 0.01, 24 SNPs were utilized as instrumental variables in causal analyses (Dataset S14).

#### BMA.

##### Data preparation.

MR–BMA adopts a multivariable framework whereby multiple exposures can be included within the model, provided a) they are each robustly associated with a least one SNP instrument used within the model, and b) they do not induce multicollinearity (22). As with univariable models, criterion a was met through inclusion of only GWS instruments, which also had a computed F-statistic of ≥10. To meet criterion b, pairwise genetic correlations (*rg*) across metabolites were computed using linkage disequilibrium score regression (LDSC) (48). In preparation for this, all GWAS summary statistics underwent a process of data munging. During this, if data were reported with a mean χ^2^ statistic <1.02, that dataset was dropped from LDSC analyses (Dataset S15) due to nonsuitability as advised by the software authors (48). Any metabolites with *rg* > 0.95 were assumed nonindependent and pruned according to the stepwise criteria outlined in *SI Appendix*, Info. S2. This resulted in nine metabolites being taken forward to MR–BMA (Dataset S4).

##### Primary analysis.

Following LDSC pruning, precurated independent mQTLs made available within the MR-Base database were extracted for each of the metabolites for use as instruments. Following removal of ApoE SNPs and removal of a SNP for which a suitable proxy (*R*^2^ > 0.8) could not be obtained, 21 instruments remained. As with univariable analyses, all SNPs were assumed to be on the positive strand, and sensitivity analyses were performed excluding palindromic SNPs.

Full details of the MR–BMA methodology can be found elsewhere (22). Briefly, with consideration of all exposures specified, MR–BMA iterates over many potentially “true” causal models, with variations of exposure subgroups included within each of these (with exposure inclusion determined by binary parameter γ). For each exposure, an MIP was computed, representing the pp of metabolite *x* appearing within the true causal model given *z* iterations. Metabolites ranked highest and with an MIP > 0.1 were interpreted as being the strongest “true causal” candidates of all those provided within the model. A MACE was also estimated, representing the estimated direct (independent) effect of metabolite *x* on outcome *y*, averaged across each pp. It is worth noting that MACE will be biased toward the null due to shrinkage applied in variable selection (22). This metric can, however, be used to gain insight into the direction of effect and magnitude relative to other metabolites included within the model. Finally, computed models were ranked by their posterior probabilities to provide best model-fit estimates for metabolite combinations and their combined association with AD. As with MIP, the highest ranked metabolite combinations, with pp > 0.1, were interpreted as showing the strongest evidence as the true causal models for metabolite combinations. For all BMA analyses, we set *z* to 10,000, the prior probability to 0.1, and prior variance (σ2) to 0.25.

##### Sensitivity analyses.

Q-statistics quantified potential instrument outliers, and Cd was used to identify influential points in the top four MR–BMA models (with pp > 0.1). Diagnostic plots were generated to investigate the predicted versus observed associations for each of the top four models. Any SNPs with Q-statistic > 10 or Cd > 0.19 (4/total SNP *N*) were flagged, and MR–BMA was repeated with the SNP(s) omitted. Metabolite–AD associations remaining after the removal of potential outliers were considered to be more reliably associated with AD.

### Post hoc Exploratory Analyses.

#### LD overlap between influential points and AD.

Any IV which demonstrates evidence of overlap with genomic regions associated with an outcome in MR analyses risks violating core MR assumptions and, in turn, calls into question IV validity. Steps were taken within primary analyses to avoid such scenarios, such as excluding any IVs associated with AD at GWS. However, influential points signpost unusually large associations, which, while could be due to particularly strong and biologically relevant associations with the exposure, may also reflect spurious factors such as shared LD with an outcome-specific genomic region. To further explore the validity of influential points, we therefore visually inspected regions of LD and cross-checked these with genes closest to top AD-related SNPs, as reported within the latest AD GWAS by Kunkle et al. (21). Briefly, summary statistics for each metabolite showing evidence of an influential point was uploaded to the publicly available visualization tool Locus Zoom (http://locuszoom.org/). LD regions were specified using the influential SNP as the reference, together with a flanking region of 400 kb. Genomic regions located below any SNP in LD with the reference point at *R*^2^ > 0.2 were cross-checked against Kunkle related genomic regions.

#### One-sample univariable MR.

Baseline NMR metabolite and AD case-control data from ADNI were obtained to allow for a small-scale, exploratory replication of significant associations observed within primary analyses. Full details regarding ADNI can be found elsewhere (24). Briefly, ADNI is a longitudinal initiative, beginning in 2003 and following participants through multiple study phases, collecting multiomic, cognitive, and phenotyping information relevant to AD risk. At baseline, metabolite information across 241 metabolite subfractions were available for almost 1,700 individuals. Metabolites demonstrating evidence of a causal association with AD within primary analyses were extracted from the wider dataset of ADNI metabolites. Genotype information were also extracted for all individuals at baseline (distinct sample *n* = 1,674). This underwent full quality control (QC) and was subsequently imputed (QC and imputation details can be found within *SI Appendix*, Fig. S10 and Dataset S16). Samples retained following QC were then merged with available metabolite data, extracting only genetic instruments utilized within primary univariable analyses and excluding samples for which metabolite information were missing (missing GP = 1 and missing HDLs = 17). Following data cleaning and merging, metabolite, genetic, and diagnostic information was available for up to 894 individuals (515 AD cases and 379 controls). Metabolite data were standardized to a mean of 0 and SD of 1, and data square root was transformed to achieve normality.

For each metabolite separately, one-sample univariable MR was performed using 2SLS. Briefly, instrumental variables were first flipped such that each represented the risk-increasing allele for the metabolite exposure of interest. Each metabolite was then regressed on all of its represented IVs, weighted by the relative strength of the genetic instrument. Predicted values from stage one were then regressed on the case/control outcome to obtain a final causal estimate. To avoid estimates being biased by selection or reverse causation (due to calculating with single-person data), stage one estimates were restricted to controls only (49). Overall IV strength for each metabolite was assessed through computation of a weighted F-statistic (IVs combined and weighted by their per-IV instrumental strength). As with primary analyses, an F-statistic < 10 was considered evidence of weak instrument bias—indicating low statistical power.

#### Association analyses for top causal metabolites.

Subsequently to performing our one-sample MR using the ADNI cohort, an additional exploratory observational analysis was performed using ADNI data for each of the metabolites identified as causal candidates within primary analyses. This was to assess whether evidence of an observational relationship between metabolites of interest and AD status could be found within the ADNI cohort. As the scope of this study was to interrogate causal relationships, we refrain from discussing the details of these observational analyses here. However, further information can be found in *SI Appendix*, Info. S4.

## Supplementary Material

Supplementary File

Supplementary File

Supplementary File

Supplementary File

Supplementary File

Supplementary File

Supplementary File

Supplementary File

Supplementary File

Supplementary File

Supplementary File

Supplementary File

Supplementary File

Supplementary File

Supplementary File

Supplementary File

Supplementary File

## Data Availability

Metabolite data used within primary analyses are publicly available within the MR-Base catalog (https://www.mrbase.org/). AD GWAS data used within primary analyses are publicly available for download at https://www.niagads.org/datasets/ng00075. Metabolite and genomic data used within post hoc analyses can be found in the ADNI database (http://adni.loni.usc.edu).

## Change History

April 23, 2021: The article text has been updated.
