# Supplementary material for: Mendelian randomization identifies blood metabolites previously linked to midlife cognition as causal candidates in Alzheimer’s disease
Source: Proc Natl Acad Sci U S A. 2021 Apr 16;118(16):e2009808118. doi: 10.1073/pnas.2009808118 (PMC8072203; doi:10.1073/pnas.2009808118)
Supplement: Supplementary File [file pnas.2009808118.sapp.pdf]

PNAS
www.pnas.org

Supplementary Information for

Mendelian randomization identifies blood metabolites previously linked to midlife cognition as causal candidates in Alzheimer's Disease

Jodie Lord[a], Bradley Jermy[b,c], Rebecca Green[a,c], Andrew Wong[d], Jin Xu[a,e], Cristina Legido-Quigley[e,f], Richard Dobson[g,h,i,j,k], Marcus Richards[d]*, Petroula Proitsi[a]*

[a] Department of Basic and Clinical Neuroscience, Maurice Wohl Clinical Neuroscience Institute, Institute of Psychiatry, Psychology & Neuroscience, King's College London, London, SE5 5AF, United Kingdom;
[b] Social, Genetic and Developmental Psychiatry Centre, Institute of Psychiatry, Psychology & Neuroscience, King's College London, London, SE5 8AF, United Kingdom;
[c] National Institute for Health Research Maudsley Biomedical Research Centre, South London and Maudsley National Health Service Foundation Trust, London, SE5 8AF, United Kingdom;
[d] MRC Unit for Lifelong Health and Ageing at UCL, University College London, London, WC1E 7HB, United Kingdom;
[e] Institute of Pharmaceutical Science, King's College London, London, SE1 9NH, United Kingdom;
[f] Systems Medicine, Steno Diabetes Centre Copenhagen, 2820 Gentofte, Denmark;
[g] Department of Biostatistics and Health Informatics, Institute of Psychiatry, Psychology and Neuroscience, King's College London, London, SE5 8AF, United Kingdom;
[h] National Institute for Health Research Biomedical Research at South London and Maudsley NHS Foundation Trust and King's College London, London, SE5 8AF, United Kingdom;
[i] Health Data Research UK London, University College London, 222 Euston Road, London, NW1 2DA, United Kingdom;
[j] Institute of Health Informatics, University College London, 222 Euston Road, London, NW1 2DA, United Kingdom;
[k] National Institute for Health Research Biomedical Research Centre at University College London Hospitals NHS Foundation Trust, 222 Euston Road, London, NW1 2DA, United Kingdom.

*Correspondence to: Petroula Proitsi or Marcus Richards
Email: petroula.proitsi@kcl.ac.uk; m.richards@ucl.ac.uk

This PDF file includes:

Supplementary Information Text S1 to S3
Figures S1 to S10
SI References

Supplementary Information Text

Info. S1. Research scope: Summarised in-line with the core checklist outlined by Burgess et al. (2)

1. **Motivation and Scope**

   a. **The primary hypothesis of interest**

   Metabolite sub-fractions previously shown to be associated with midlife cognitive functioning lie on the causal pathway to later Alzheimer's Disease diagnosis. As such, intervening on levels of these metabolites prior to disease onset will go some way to reducing Alzheimer's Disease risk.

   b. **Motivation for the study**

   The absence of disease modifying therapeutics for Alzheimer's Disease (AD) continues, and an understanding of early, easily accessible biomarkers to inform treatment strategies remains sparse. Using knowledge of associations between pre-clinical risk factors and potential biomarkers and assessing how well such markers translate through to later clinical risk could therefore hold special utility in informing early treatment intervention, particularly if a causal relationship can be shown. Midlife cognitive factors have consistently been shown to predict later AD risk; thus selecting candidate AD biomarkers based on associations with these earlier factors could hold particular promise. More specifically, associations with blood metabolites are of particular interest due to their ease of accessibility via a simple blood sample, making both routine measurement and intervention possible. Past epidemiological studies have implicated metabolites – particularly lipids – in AD, but causal relationships have yet to be established. Using knowledge from previously observed associations between a number of metabolites and mid-life cognition in our 2014 and 2018 studies (3)(4), we therefore wanted to investigate how well such metabolic markers translate through to later AD diagnosis, and as such investigate their utility as AD-relevant biomarkers.

   c. **Motivation for using Mendelian Randomization**

   Mendelian Randomization (MR) is an accessible method for assessing causality in instances where controlled randomized trial data are not available. Unlike alternative genetically inspired methods, such as LD-score regression (1) and genetic co-localisation (5), which uncover potentially shared genetic aetiology between two traits, MR is unique in its ability to provide information on the direction of the causal effect, and the likely magnitude of impact upon exposure intervention specifically.

   Given that this study is interested in investigating whether metabolites which demonstrate association with cognitive processes prior to AD onset translate to causally impact later AD risk, MR offers an appropriate method which can utilise readily available genome-wide association data to test such a causal hypothesis.

d. The study scope

Conduct a preliminary causal analysis using MR methodology, to:

i. Investigate whether any one of the metabolite subfractions previously found to be associated with mid-life cognition show evidence of having a causal effect on clinically diagnosed AD.

ii. Investigate whether sub-groups of metabolites, of those previously found to be associated with mid-life cognition, together show evidence of having a causal effect on clinically diagnosed AD.

iii. Investigate the possibility of reverse causation. That is, whether clinically diagnosed AD shows evidence of causally impacting levels of metabolites, rather than vice versa.

e. The primary analysis (what and how many)

i. Univariable bidirectional Mendelian randomization to assess the causal relationship between 19 metabolite subfractions – one at a time – and clinically diagnosed Alzheimer's Disease (AD).
38 univariable analyses conducted in total:
19 * metabolite → AD
19 * AD → metabolite.

ii. Bayesian model averaging MR to assess potential groups of metabolites which may together be on the causal pathway to AD and, again, to assess per-metabolite-AD causal relationships using a method better equipped to handle high correlation amongst risk factors. 1 analysis conducted in total, consisting of 9 metabolite risk factors, each genetically correlated <95%.

2. Data Sources

For both sets of primary analyses outlined in section 1d, a two-sample MR approach was adopted, selecting publicly available genome-wide summary statistics for each metabolite, and separately for clinically diagnosed AD. To the best of our knowledge, no sample overlap existed between the metabolite and AD datasets. Populations were also comparable across each of the datasets – being of white, European ancestry.

For metabolites and AD, the latest and largest peer-reviewed GWAS datasets were utilised in an attempt to obtain the greatest statistical power. More specifically, Kettunen et al (6) was selected due to the relevance of metabolite subfractions and their quantification method (Nuclear Magnetic Resonance spectroscopy) which matched that of the observational data for which metabolites were selected on the basis of (4). In this way, it allowed for the direct comparison between those metabolites previously shown to associate with midlife cognition, and how they may translate across to causally associate with clinically relevant AD.

3. Selection of genetic variants

a. GWAS selection (including p-value thresholding / clumping)

GWAS summary statistics were utilised to select instrumental variables using a genome-wide approach. This was favoured over that of a candidate gene-region(s)

strategy due to the polygenic nature of the phenotypes of interest. For both metabolites and AD, multiple SNP-phenotype associations have been shown to exist, spanning a number of regions across the genome (6)(7). With the exception of APOE – a genomic region located on chromosome 19 with an unusually large effect size for its association with AD (7) – per-SNP effect sizes also remain small, making a pooled IV approach which exploits the large power gains of GWAS most appropriate in this instance.

To ensure robustness of instrumental variables and to avoid introduction of pleiotropy, only SNPs which were significantly associated with each exposure at the level of genome-wide significance ($5*10^{-08}$) were considered as instruments.

Clumping procedures differed slightly between metabolite exposures and AD. For metabolites, instruments were selected using a list of pre-curated metabolite quantitative trait loci (mQTLs) extracted from Kettunen et al (6) and made available within the MR-Base catalogue. Pre-curated instruments were not available for AD data, and thus genome-wide significant instruments were clumped using an r2 threshold of 0.001.

b. Exclusion criteria

i. SNPs with a computed F statistic <10.
ii. Any exposure with only a single instrumental variable
iii. SNPs significant with exposure at $p>5*10^{-08}$
iv. SNPs in linkage disequilibrium >r2=0.001
v. SNPs associated with the outcome at $p<5*10^{-08}$
vi. SNPs with known pleiotropy (e.g. ApoE)

c. Assessment of instrumental validity

i. Computation of per-instrument F statistic
ii. Sensitivity analyses → leave-one-out, MR-Egger, MR-PRESSO, Weighted median, Cochran's Q, Cooks Distance.

4. **Harmonization procedure**

Data were harmonized prior to all MR analyses, where all inferable SNPs were aligned across the exposure and outcome dataset. Palindromic SNPs were retained and assumed to be on the forward strand, and additional sensitivity analyses were conducted which removed all palindromic SNPs during harmonization and re-computed causal estimates.

5. **Primary analysis and multiple testing**

As stated in section 1d, the primary analyses conducted in this study were:

a. Univariable bidirectional Mendelian randomization to assess the causal relationship between 19 metabolite subfractions – one at a time – and clinically diagnosed Alzheimer's Disease (AD).
38 univariable analyses conducted in total:
19 x metabolite → AD
19 x AD → metabolite.

These analyses identified four metabolites to be significantly causally associated with AD at the adjusted level of p<0.009: XL.HDL.FC, XL.HDL.PL, XL.HDL.P, XL.HDL.P. A number of additional metabolites were also associated at the 5% level, including GP, a number of large HDLs, and XL.HDL.C.

b. Bayesian model averaging MR to assess potential groups of metabolites which may together be on the causal pathway to AD and to again assess per-metabolite-AD causal relationships using a method better equipped to handle high correlation amongst risk factors. 1 analysis conducted in total, consisting of 9 metabolite risk factors, each genetically correlated <95%.

Of the four metabolites demonstrating adjusted significance in univariable analyses, only 1 – XL.HDL.FC – was taken forward to Bayesian analyses, as the remaining 3 were pruned out due to high correlation. XL.HDL.FC was identified as the third highest ranked "true causal" metabolite by Bayesian analyses, with GP identified as the most strongly ranked, followed by XL.HDL.C.

Multiple testing was corrected for using an adjusted alpha of 0.009. This was calculated using an independent tests package within Python (https://github.com/hagax8/independent_tests) which computes an adjusted p-value threshold whilst accounting for correlations amongst metabolites (see supplementary information (SI3)).

6. **Sensitivity analyses**

A number of sensitivity analyses were conducted to assess the validity of primary analyses and instrumental variables.

a. For univariable analyses:

i. Robust methods: MR-Egger and Weighted Median

ii. MR Pleiotropy RESidual Sum and Outlier (MR-PRESSO)

iii. Cochran's Q-statistic

iv. Leave-one-out analyses

v. (post-hoc) Small scale 1-sample replication using individual level data from an independent cohort

b. For Bayesian model averaging MR:

i. Cook's Distance

ii. Q-statistics

7. **Data presentation**

All data output is provided either within the main manuscript or supplementary material for the scrutiny of readers. Results which are statistically significant as well as those which do not reach significance are reported across all results in order to provide complete transparency and avoid reporting bias. Charting visualisations are provided in the format recommended within the MR literature to ensure maximum transparency and interpretability of results. For example, scatter plots have been made available for all IVW, Egger and Weighted Median

analyses (see supplementary figures F1a-F1s). From these, each figure confirms at a glance (1) the extent of instruments available for univariable analyses, (2) the precision of per-instrument estimates, (3) the level of agreement between IVW and robust method estimates, (4) any obvious influential points, and (5) the extent of pleiotropy as indicated by the Mr-Egger intercept. All results are also available within tabular format for use in further analyses and for the interrogation of the reader where required.

Interpretation

Primary univariable analyses indicated four XL.HDLs to be on the causal pathway to AD (XL.HDL.FC, XL.HDL.PL, XL.HDL.P, XL.HDL.L), significant at the adjusted significance threshold of $p<0.009$. These demonstrated an effect in the negative direction, indicating a protective effect, though their individual magnitude of effect was small (OR range: 0.86-0.89). Bayesian model averaging largely corroborated univariable analyses, placing XL.HDL.FC, together with XL.HDL.C within the top three causal metabolites, with agreed direction to that of univariable MR. GP was identified by Bayesian model averaging as showing the strongest evidence of a causal association with AD, with the association being in the positive direction, indicating a risk increasing effect of GP levels on AD. GP also demonstrated a nominally significant positive association with AD in primary univariable analysis, with $p=0.0099$, and was the only metabolite to replicate in a small scale replication using individual level data from an independent cohort. Like XL.HDLs, the magnitude of effect was however small (95% CI=1.045-1.375), indicating that this metabolite may explain a piece of the causal puzzle, but that it alone does not explain the whole story. Sensitivity analyses – which are largely conservative in comparison to primary analyses - demonstrated consistent direction of effect for significant results but failed to retain significance. However, specific tests of pleiotropy indicated that this was due to lack of power rather than notable violations to instrumental assumptions. Our study therefore offers a number of interesting causal candidates – namely XL.HDLs and GP which may hold value as early indicators of AD risk, and possible early targets of intervention. Future studies with greater statistical power and which incorporate a wider network of risk factors will, however, be important for building upon the foundations within this study.

Info. S2. Metabolite Pruning in preparation for Bayesian Model Averaging Mendelian Randomization.

Bayesian Model Averaging MR (BMA-MR) adopts a multivariable framework, whereby multiple exposures can be included within the model, provided a) they are each robustly associated with a least one SNP-instrument used within the model, and b) they do not induce multi-collinearity. As with univariable models outlined within the main text of the present paper, criterion a) was met through the inclusion of only those exposures which had at least five GWS SNPs available, each with a minimum F statistic of 10. To meet criterion b), pairwise genetic correlations (rg) across metabolites were computed using linkage-disequilibrium score regression (LDSC)(1), and any metabolites observed with rg>0.95 assumed non-independent and pruned according to the following stepwise procedure:

1) For metabolite pairs with *rg*>0.95, if one metabolite demonstrated a greater number of *rg*>0.95 with other metabolites, that metabolite was removed.
2) For metabolite pairs with *rg*>0.95 *and* an equal number of *rg*>0.95 with other metabolites, the metabolite with the greater number of correlations at adjusted significance (*p*<0.002 – see 2.4) was removed.
3) For *rg*>0.95 metabolite pairs which also had an equal number of wider metabolite pairwise *rg*>0.95 at adjusted significance, the metabolite with the greatest number of nominally significant *rg*s (*p*<0.05) was removed.

During data preparation, MUFA was dropped from further analyses due to a low mean chi-square statistic (χ =1.01) computed during LDSC data munging, making it unsuitable for cross-trait LDSC analyses.

Info. S3. Multiple test corrections

An adjusted p-value, correcting for multiple independent tests while accounting for correlations amongst metabolites, was computed using an “independent_tests” package in Python (https://github.com/hagax8/independent_tests). An adjusted significance of p<0.009 was calculated for primary analyses, as per the following:

1. The number of principal components explaining 99.5% of the variance(N) within the squared correlated matrix was computed (N=8).
2. N of individual tests (T) within the squared correlation matrix was then calculated using formula: T=(N*N-N)/2 (T=28).
3. The square root of T was then used to establish the final number of individual metabolites (m) to correct for (m=√* (m=5.29)).
4. A final Bonferroni corrected alpha (α) was then computed by dividing m by 0.05 (α=0.009).

For post-hoc MR analyses, in which univariable 2-stage least squares MR was performed across five metabolites, an adjusted significance of p<0.02 was computed, as per the following:

1. N=4
2. T=6
3. m=2.45
4. α=0.02

Info. S4. Post-hoc observational analysis

Table S1 outlines the results of five logistic regression analyses performed using baseline metabolite and sample information from the Alzheimer's Disease Neuroimaging Initiative (ADNI) (8). Each model was adjusted for age, sex, and the APOE4 genotype (dummy coded, with 0 as the reference category). Samples were restricted to those which (i) had available metabolite data at baseline, and (ii) were classified as either AD cases or clinically healthy controls. As genotype information was not required within this phase of analyses, retained sample sizes were larger than those available within the same study cohort utilised for two-stage least squares Mendelian randomization (glycoprotein acetyls (GP) N=1,140, high-density lipoproteins (HDLs) N=1,116. See table S2 for confirmation of pre-processing steps).

As with our exploratory 2-stage least squares MR performed on the same sample, GP was the only metabolite to demonstrate a statistically significant association with AD status in observational analyses ($p=8x10^{-05}$). This relationship was in the expected, positive direction, with an odds ratio of 1.3. No HDLs demonstrated a statistically significant association, with confidence intervals spanning both a positive and negative direction of effect. Authors advise caution in the interpretation of observational results when assessing causality, however, as possible unmeasured confounding and reverse causation can result in spurious associations, misdirection of effect, and diluted associations due to lack of precision.

Table S1. Results across five logistic regression analyses, one for each metabolite which demonstrated evidence of a causal association with AD status in primary MR analyses. Each model is adjusted for age, sex, and APOE4 carrier status (0,1 or 2), and all metabolites are square root transformed and standardised to a mean of 0 and SD of 1.

**Metabolite**	**OR**	**p-val**	**ci_95_lower**	**ci_95_upper**
GP**	1.299	8.13E-05**	1.169	1.429
xl.hdl.l	1.040	0.635	0.877	1.203
xl.hdl.p	1.040	0.641	0.876	1.203
xl.hdl.fc	1.023	0.778	0.865	1.180
xl.hdl.pl	0.992	0.917	0.831	1.152

**p<0.01

Table S2. Pre-processing steps and resulting sample retention

**Pre-processing step**	**Removed**	**Remaining N**	**Additional notes**
Starting values	NA	1697	
Missing covariate info	2	1695	
MCIs / non-AD dementia	506	1189	678 cases. 511 controls
Missing metabolite values	26	1163	HDLs only. 666 cases. 497 controls

Fig. S1a.

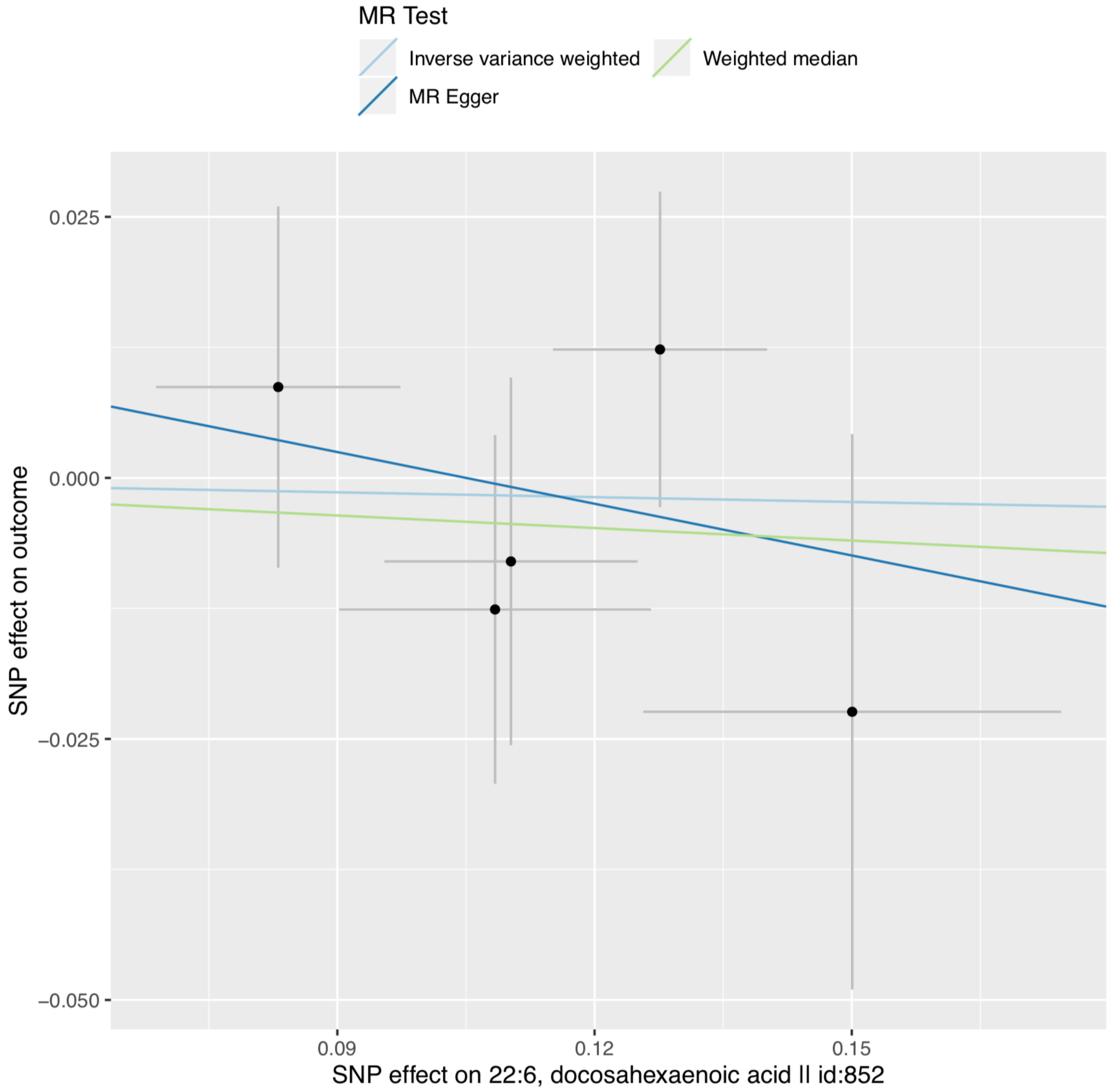
MR Test
Inverse variance weighted
Weighted median
MR Egger
0.025
0.000
−0.025
−0.050
SNP effect on outcome
0.09
0.12
0.15
SNP effect on 22:6, docosahexaenoic acid || id:852

Fig. S1b.

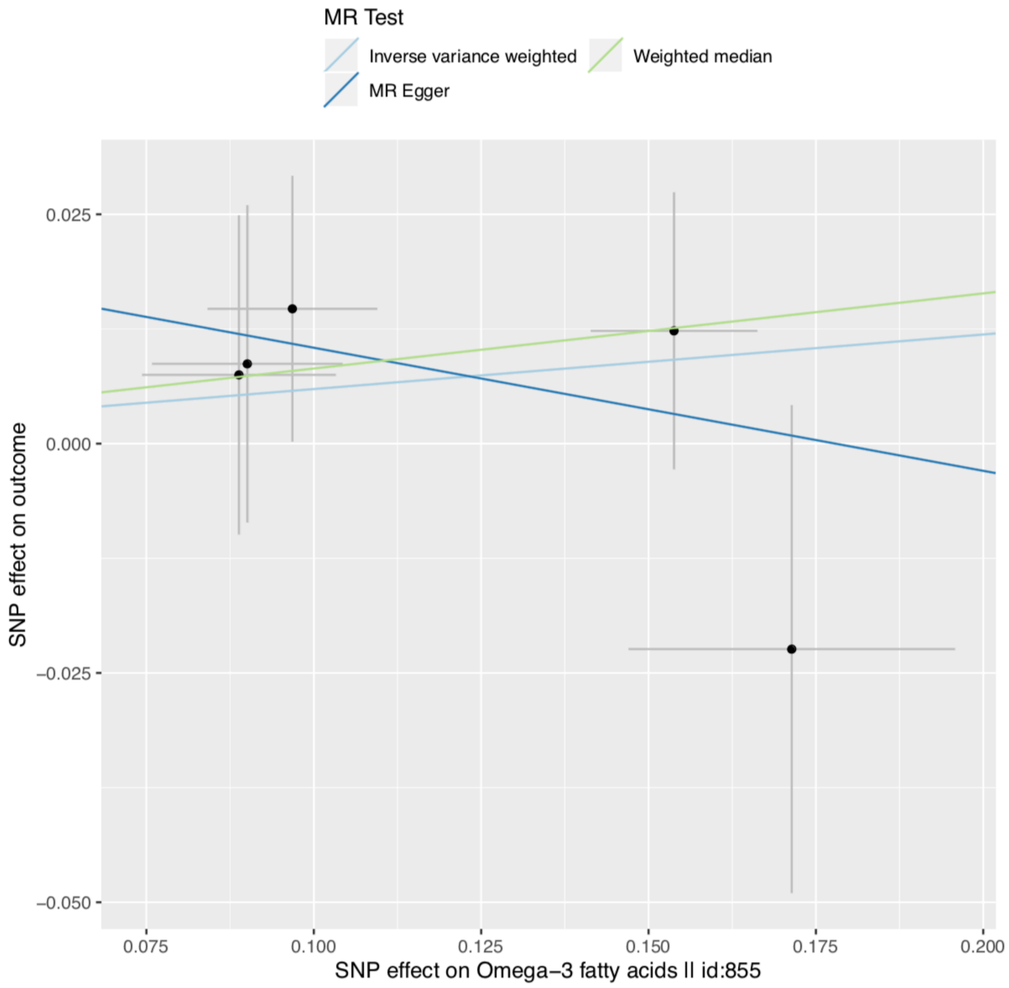

Fig. S1c.

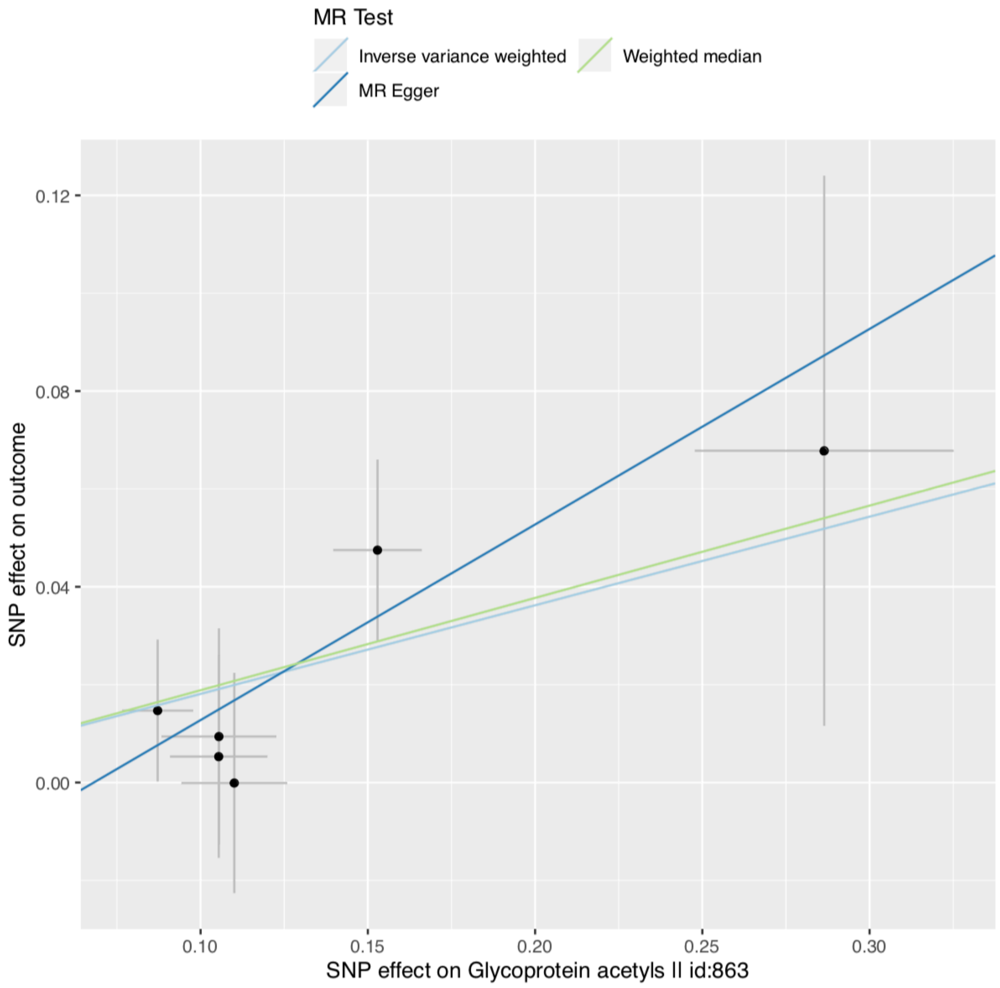

Fig. S1d.

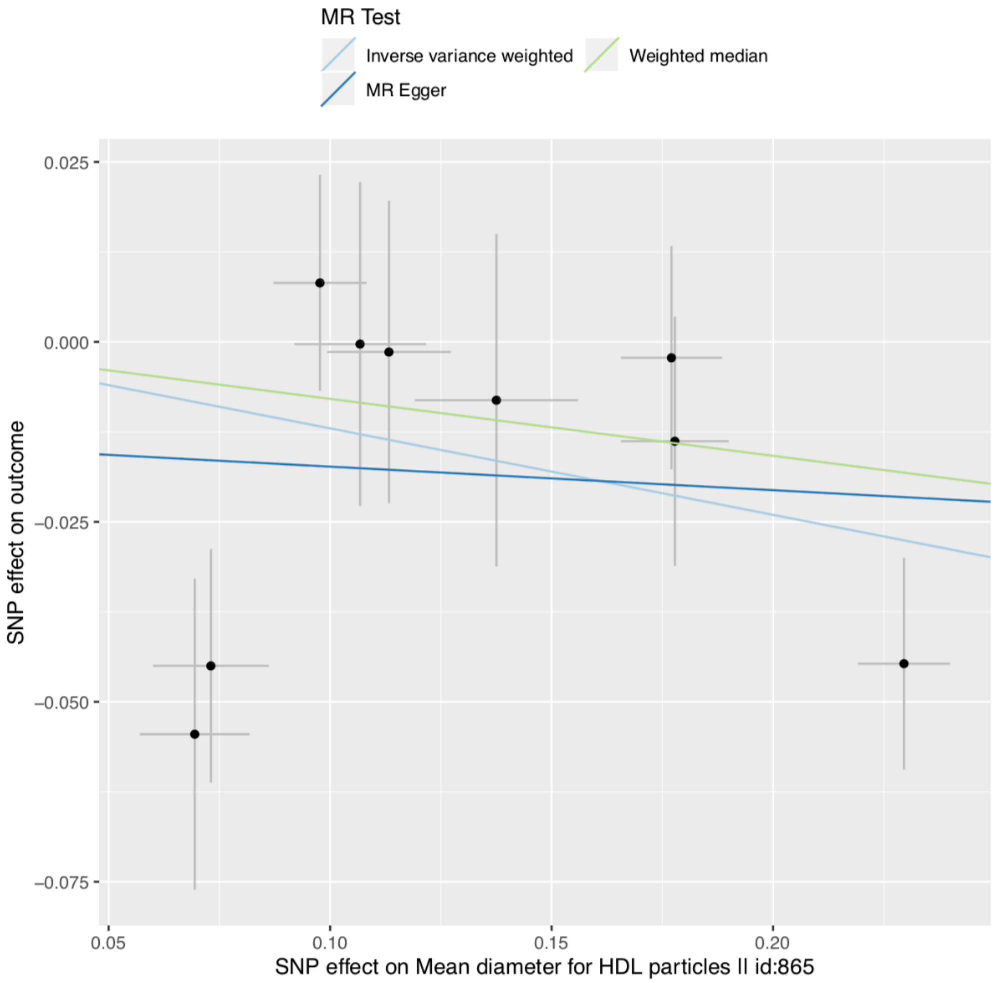

Fig. S1e.

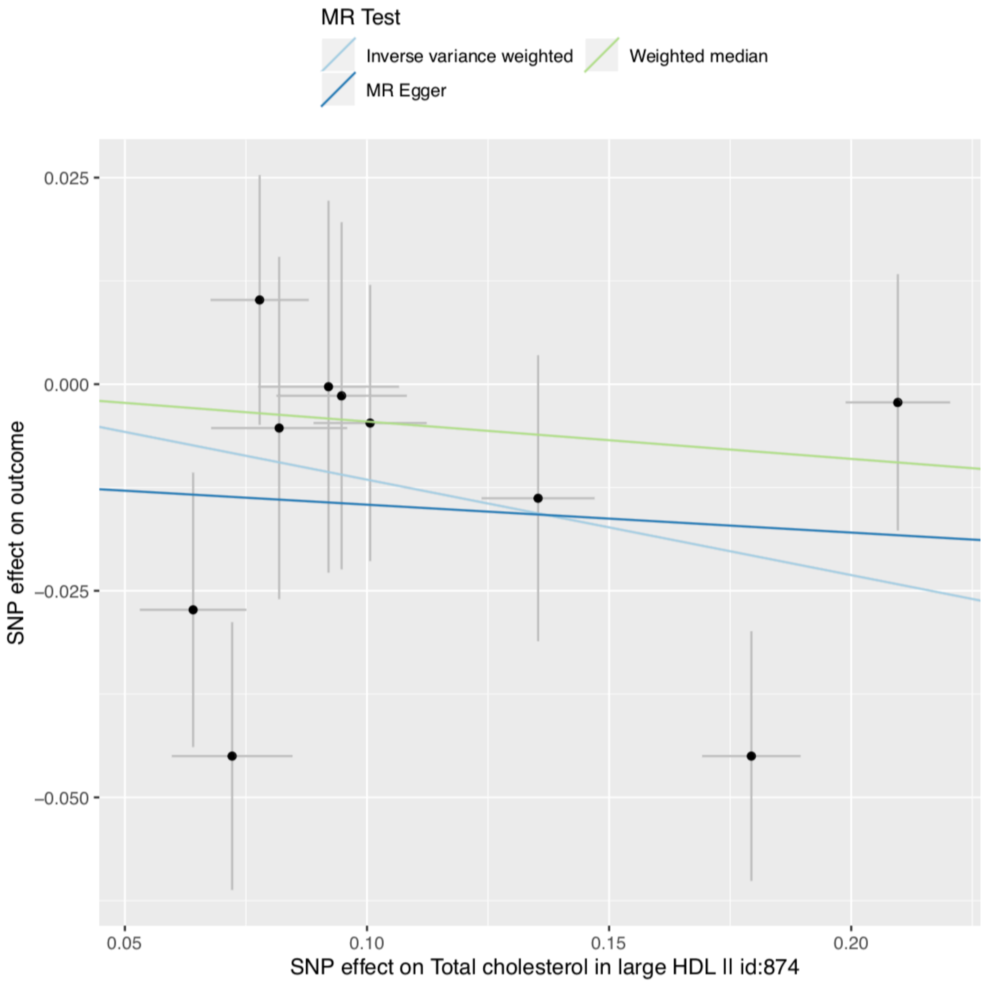

Fig. S1f.

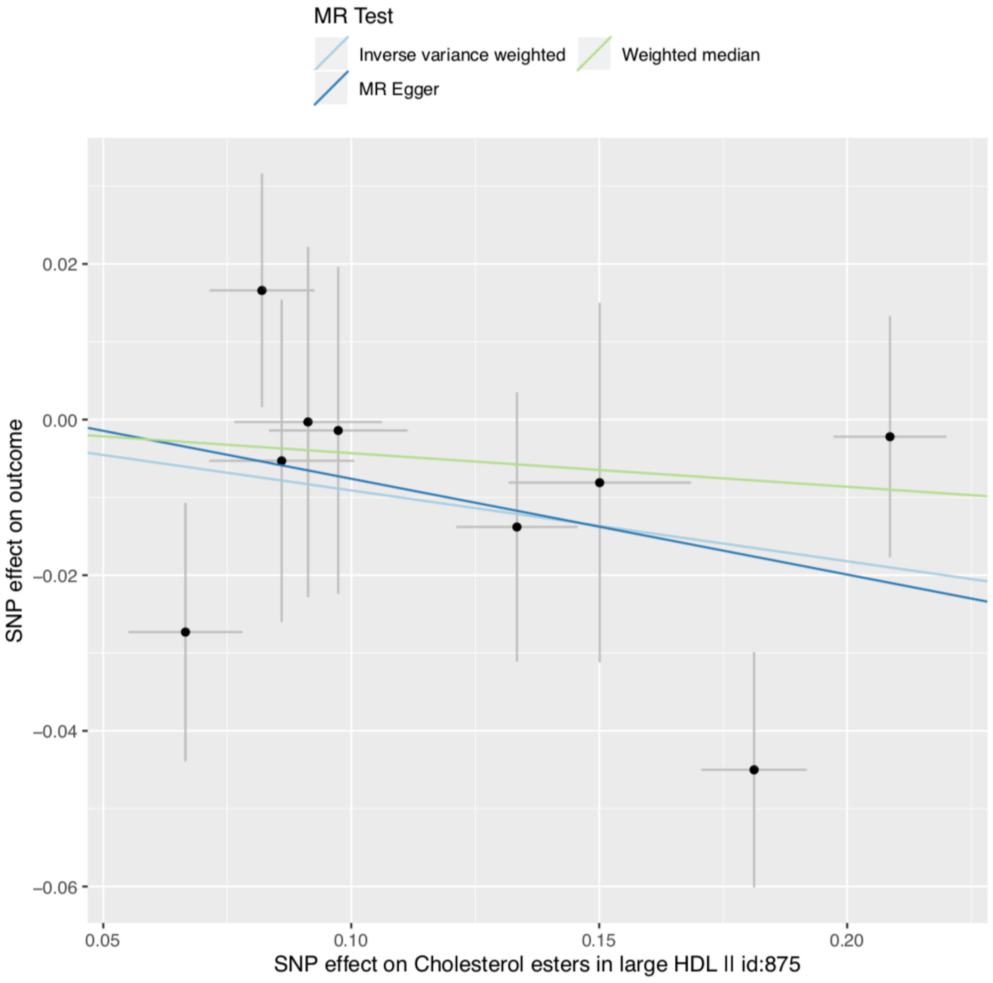

Fig. S1g.

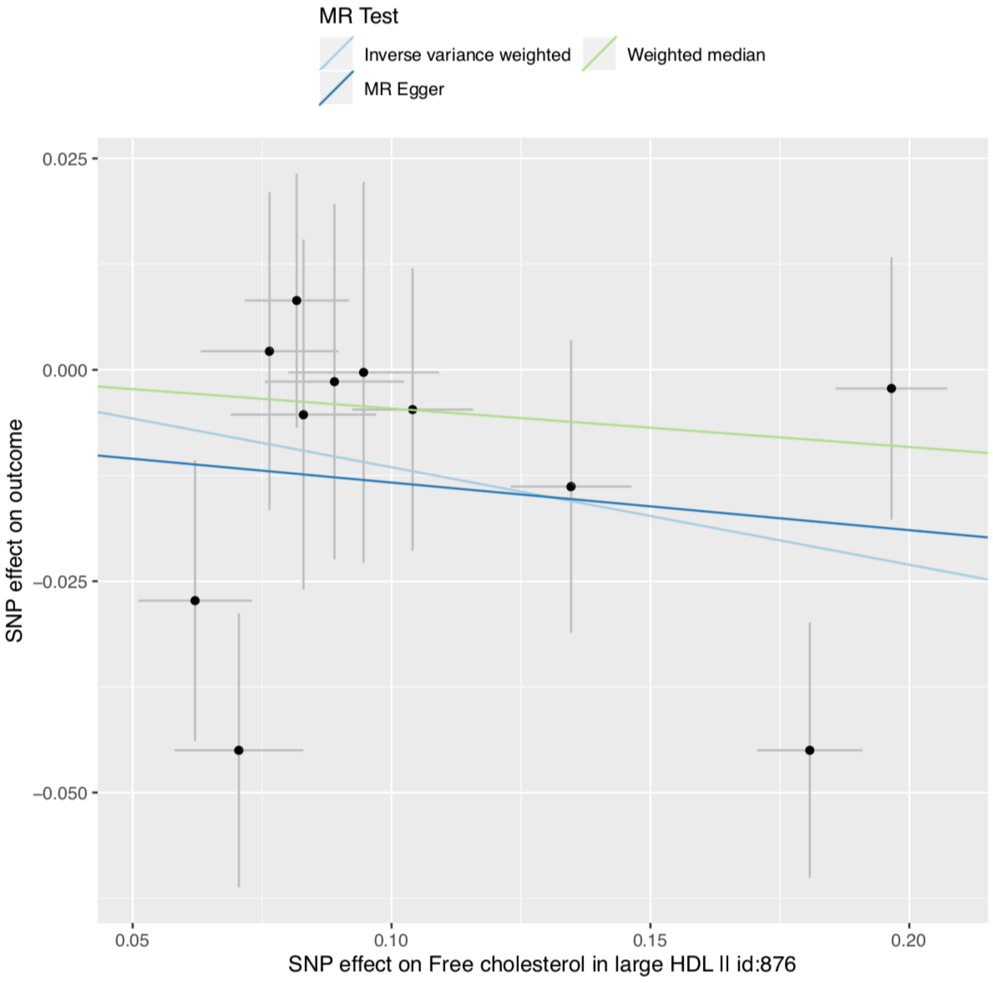

Fig. S1h.

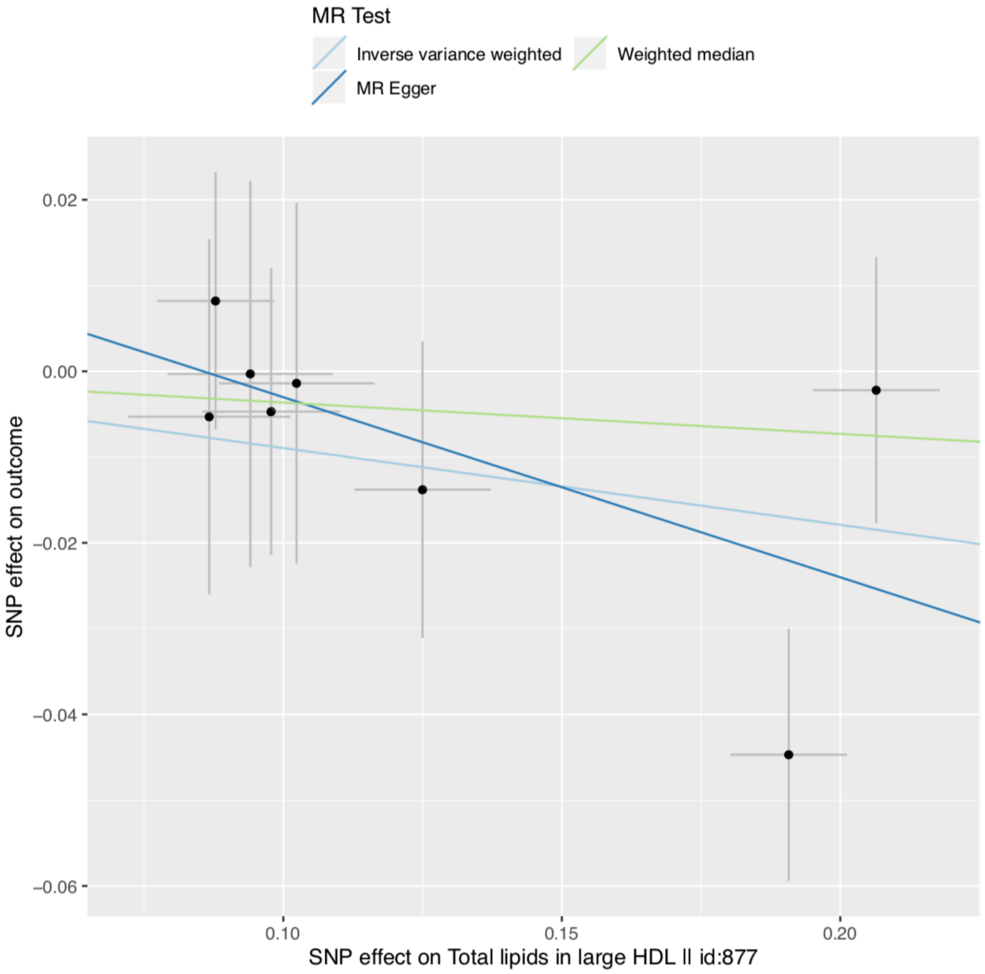

Fig. S1i.

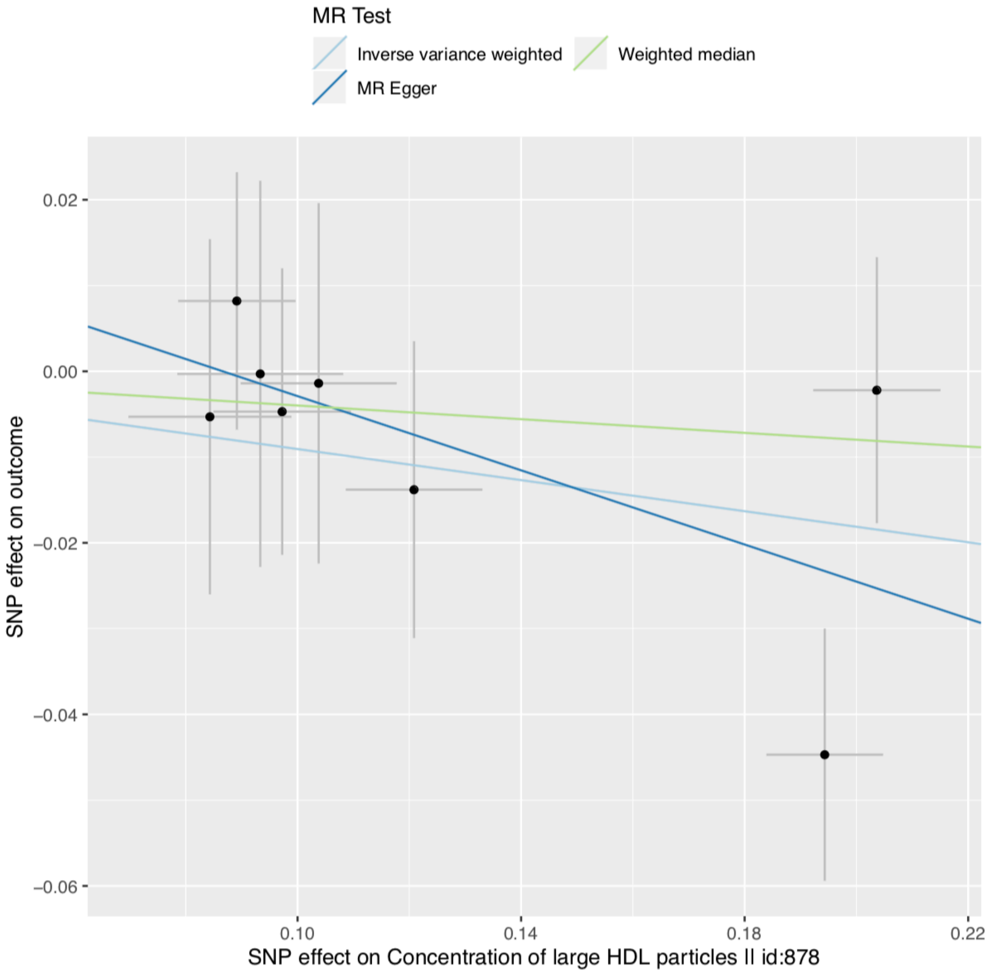

Fig. S1j.

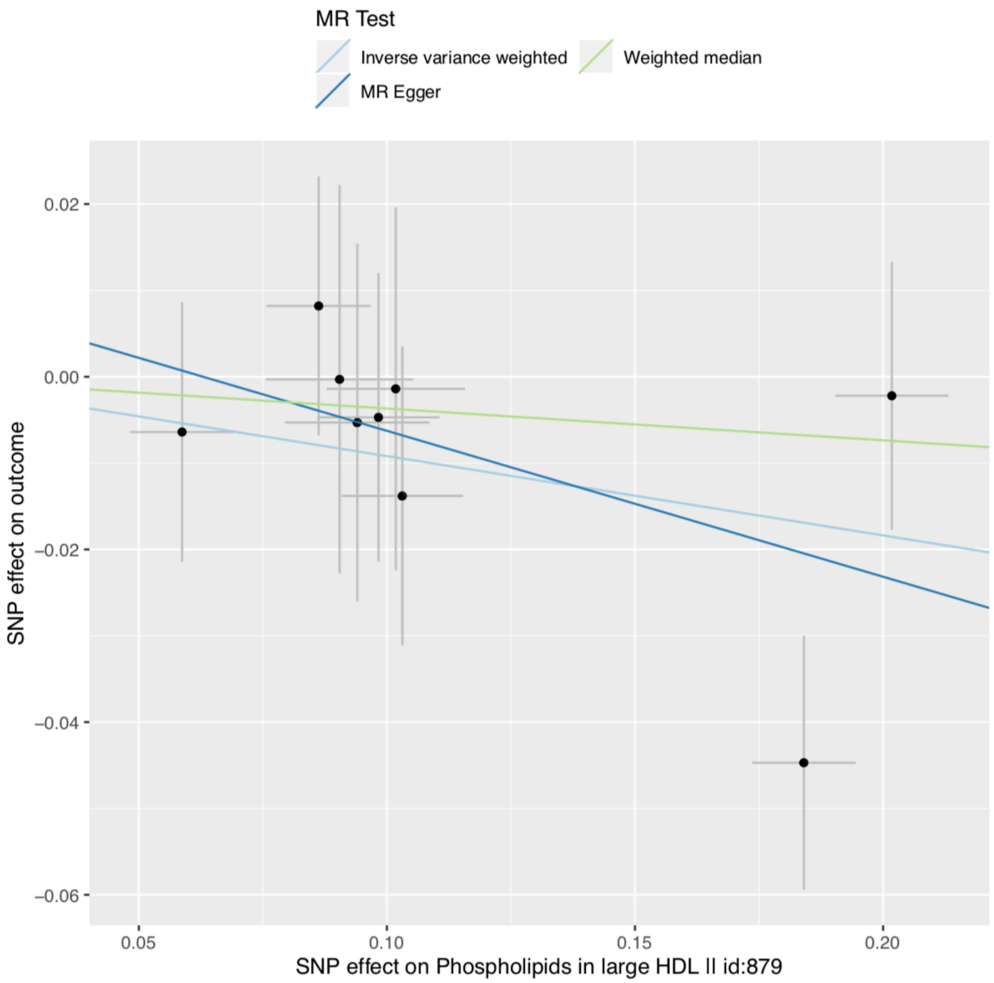

Fig. S1k.

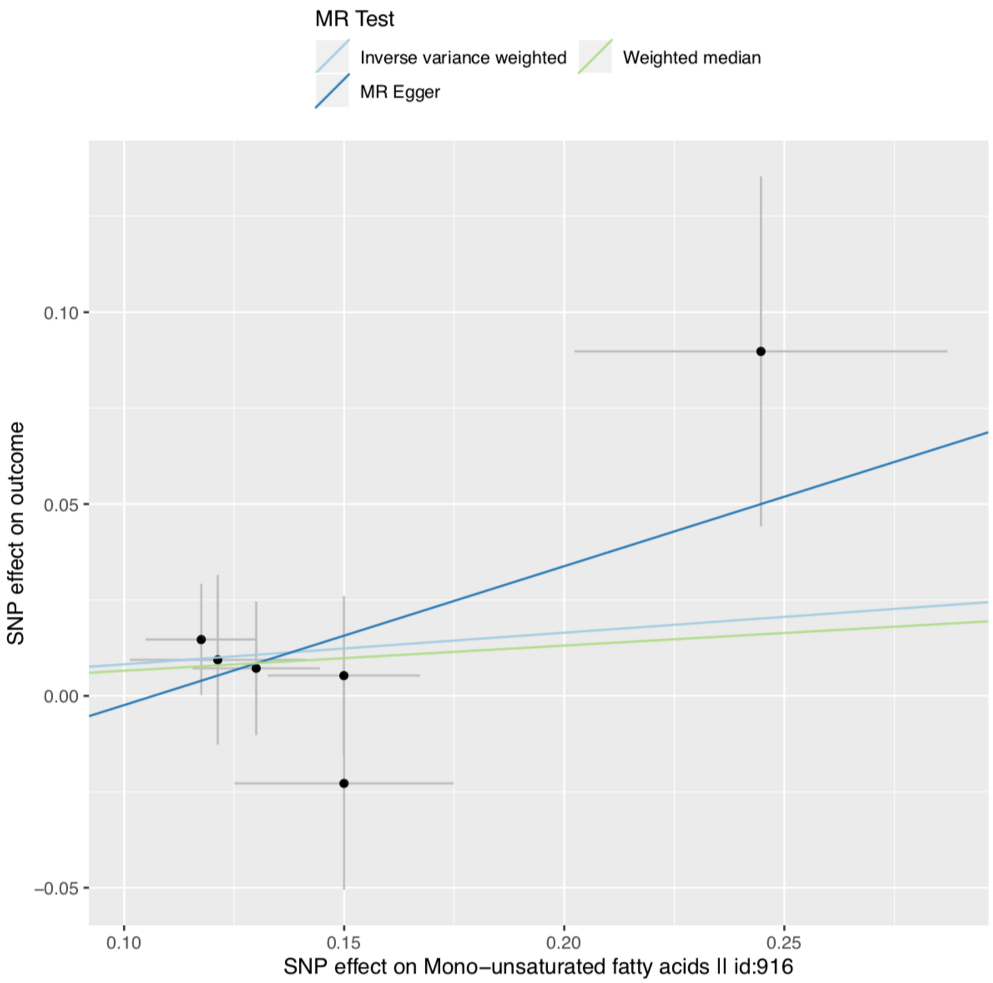

Fig. S1l.

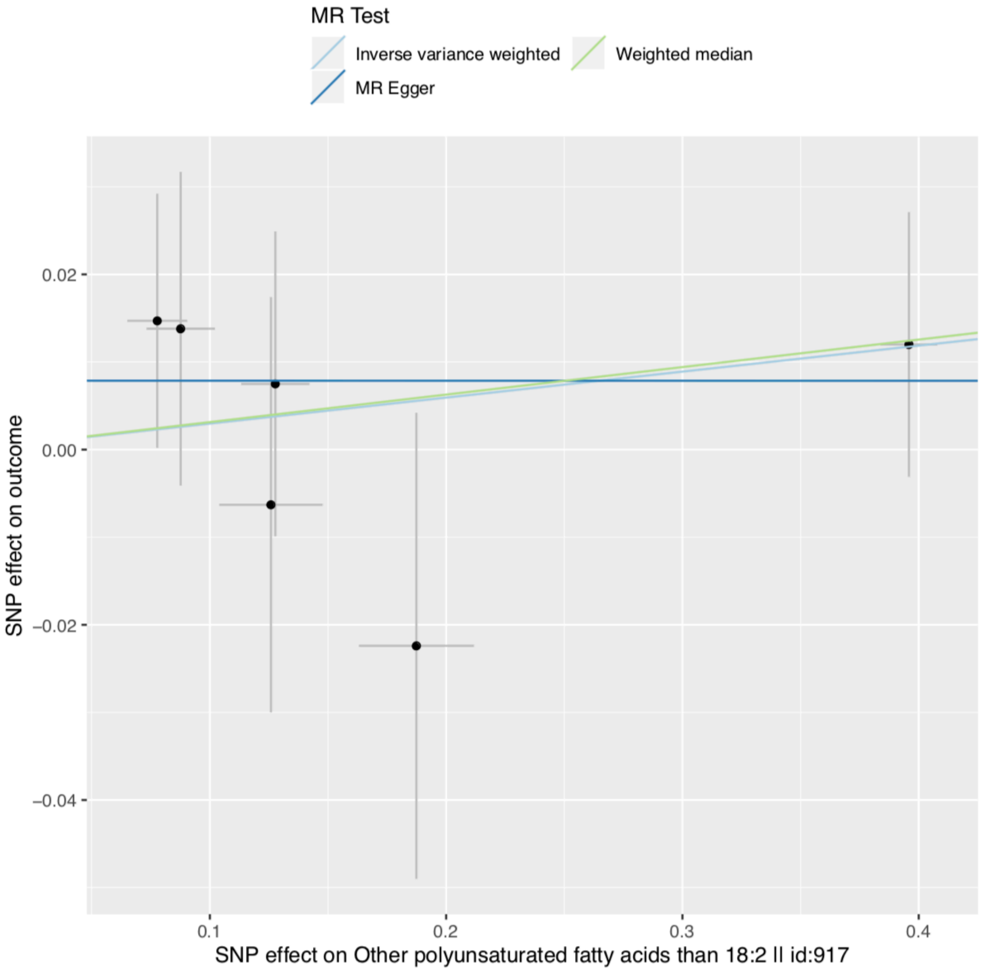

Fig. S1m.

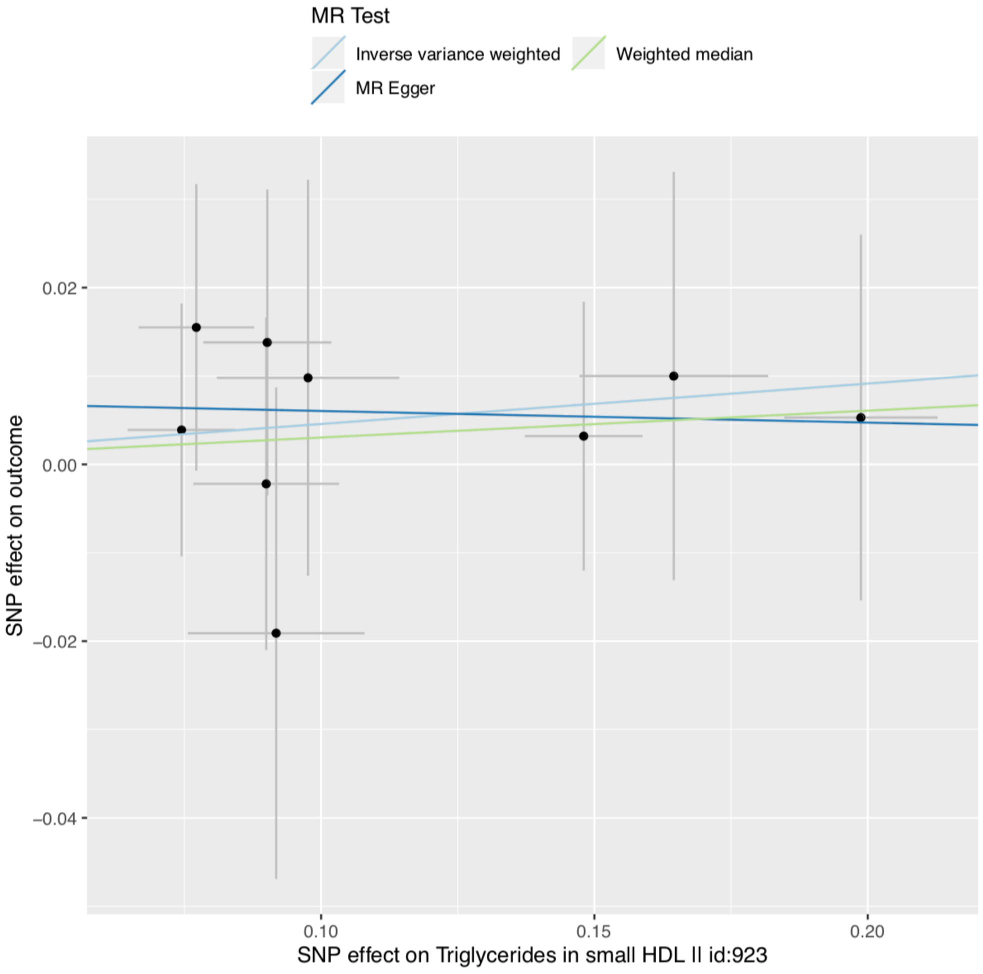

Fig. S1n.

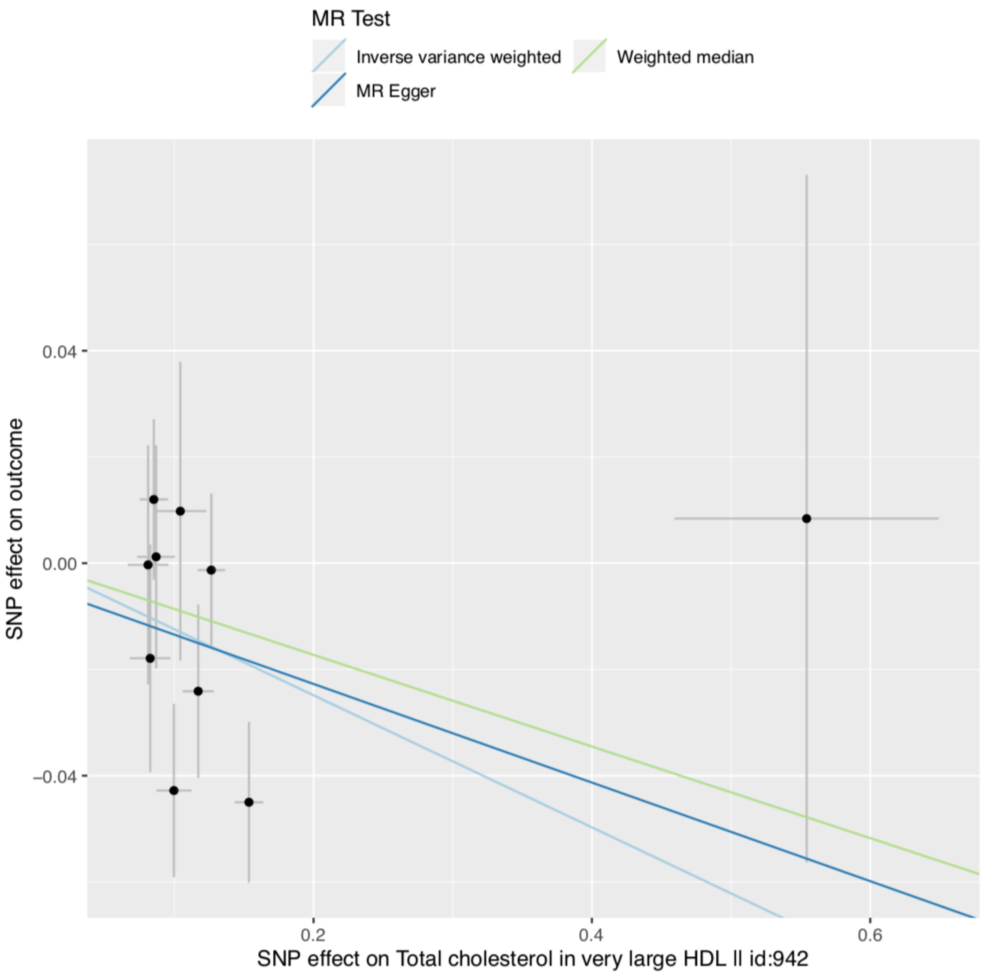

Fig. S1o.

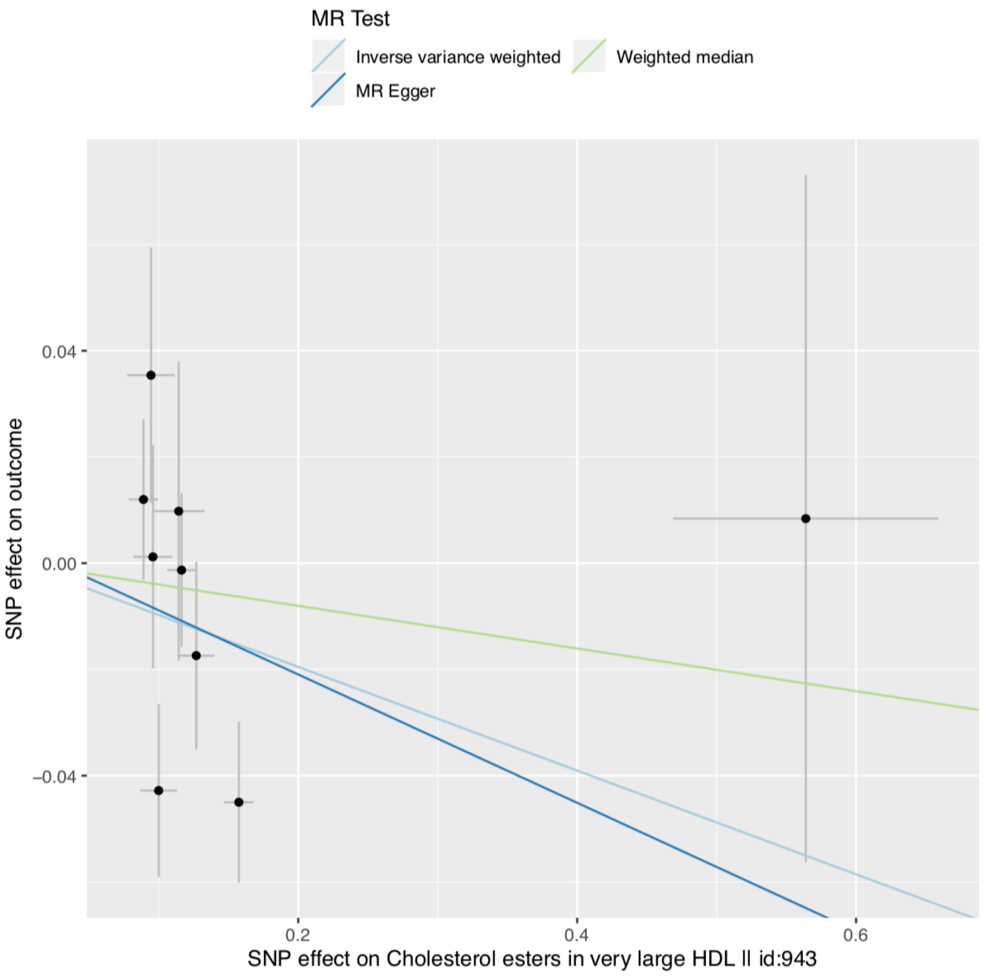

Fig. S1p.

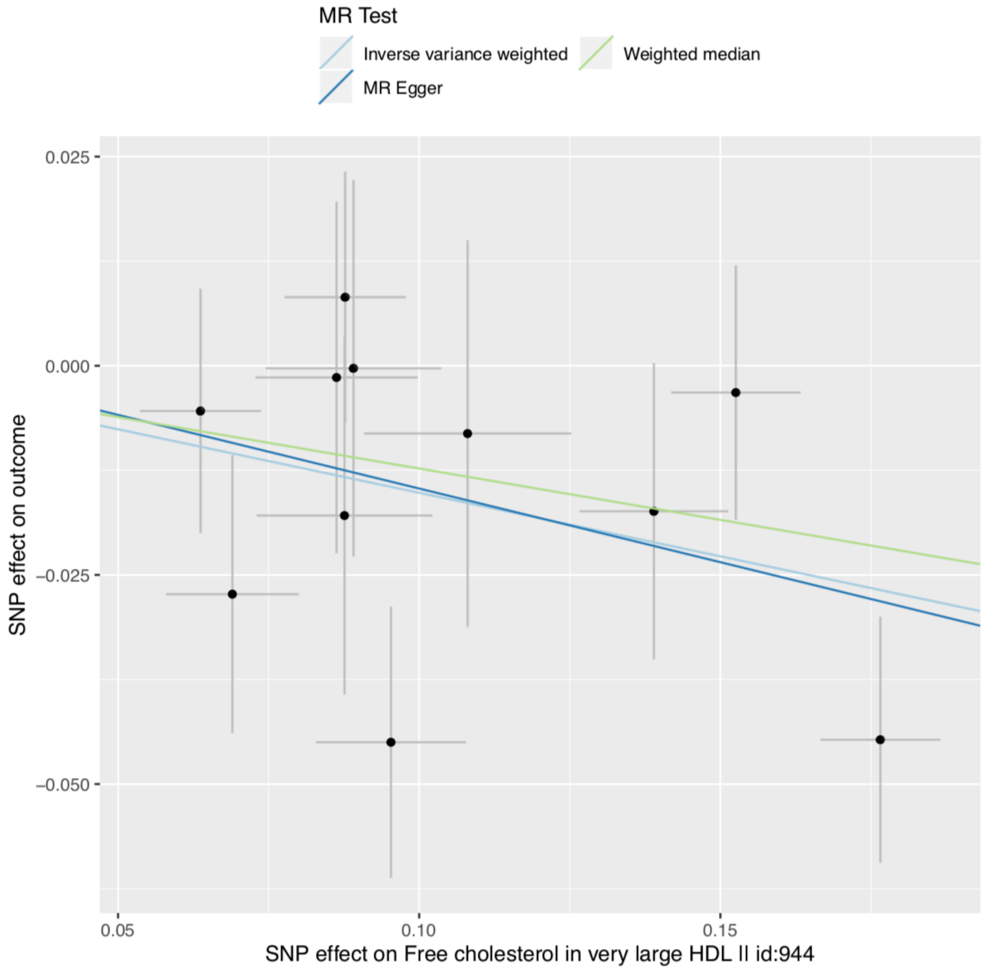

Fig. S1q.

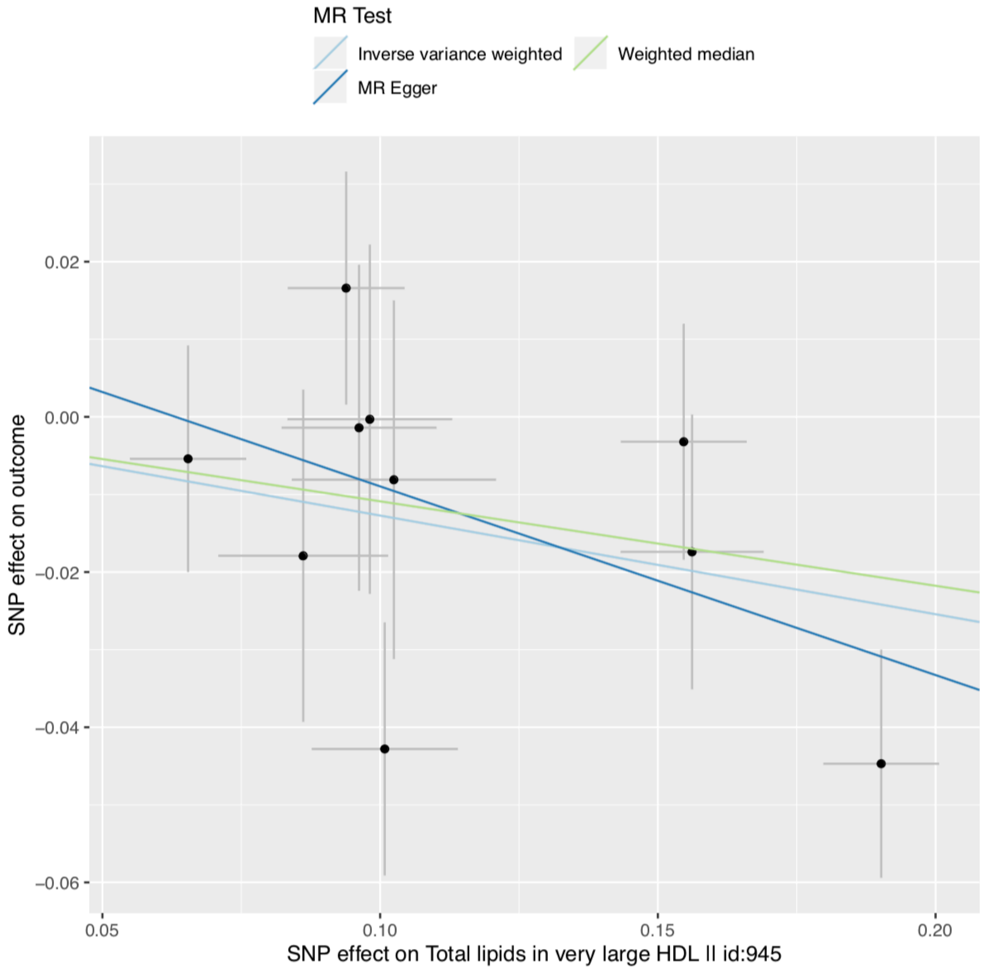

Fig. S1r.

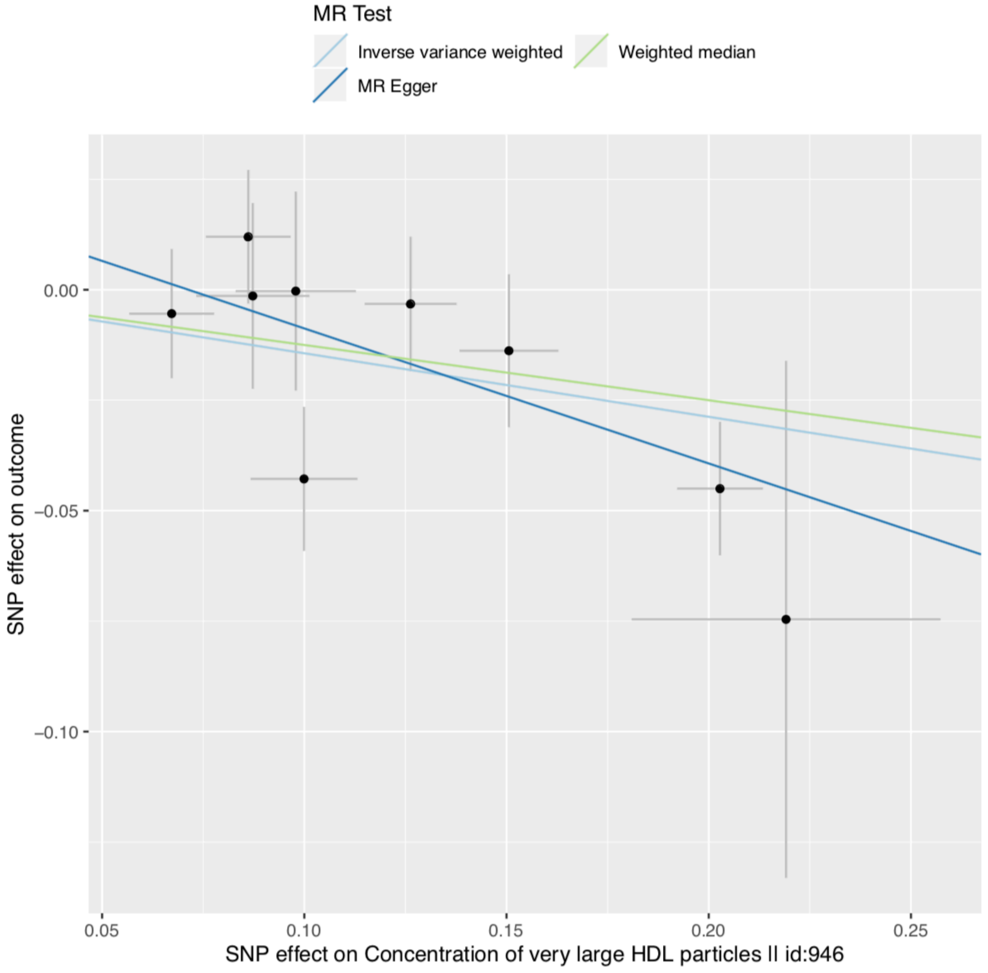

Fig. S1s.

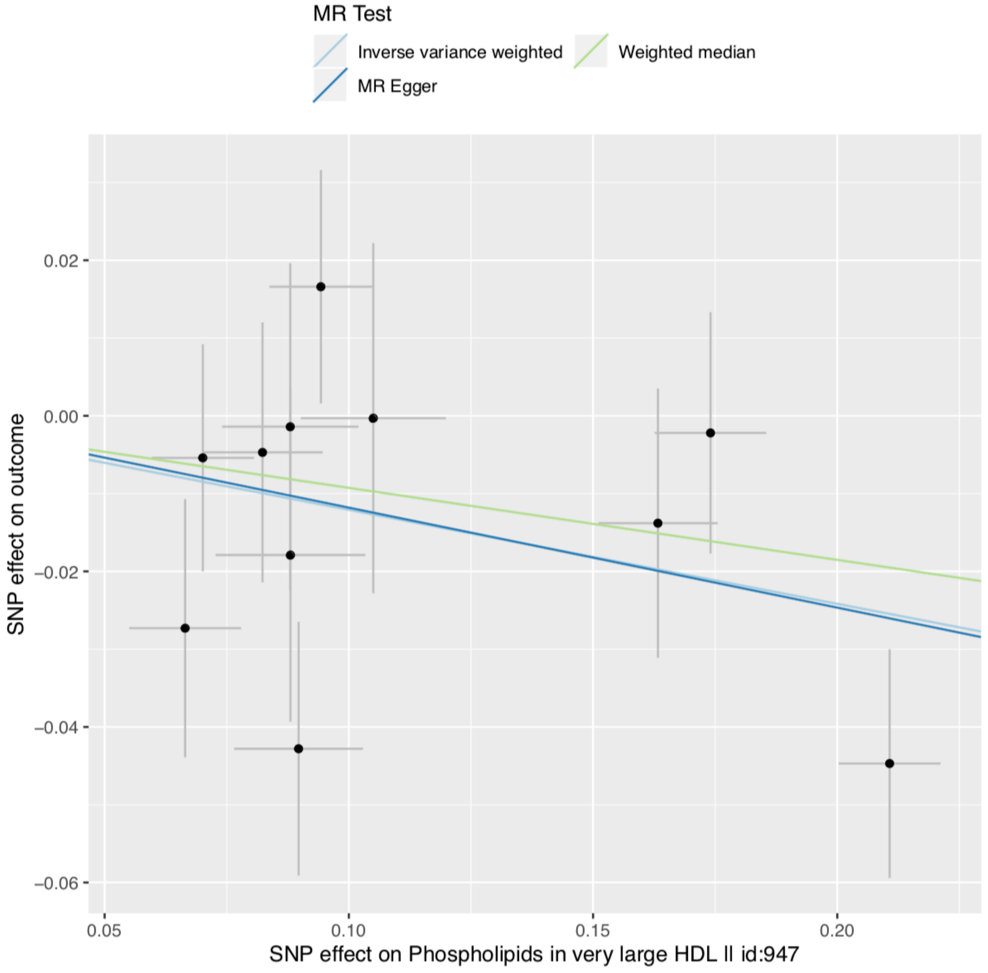

Fig. S1a-S1s. Scatterplots of the gene-AD versus gene-metabolite associations for each metabolite-AD pair when metabolite is the exposure and AD the outcome following univariable MR analyses.

Each point in the scatter plot represents an instrumental SNP and different regression lines represents the different MR methods used.

Fig. S2

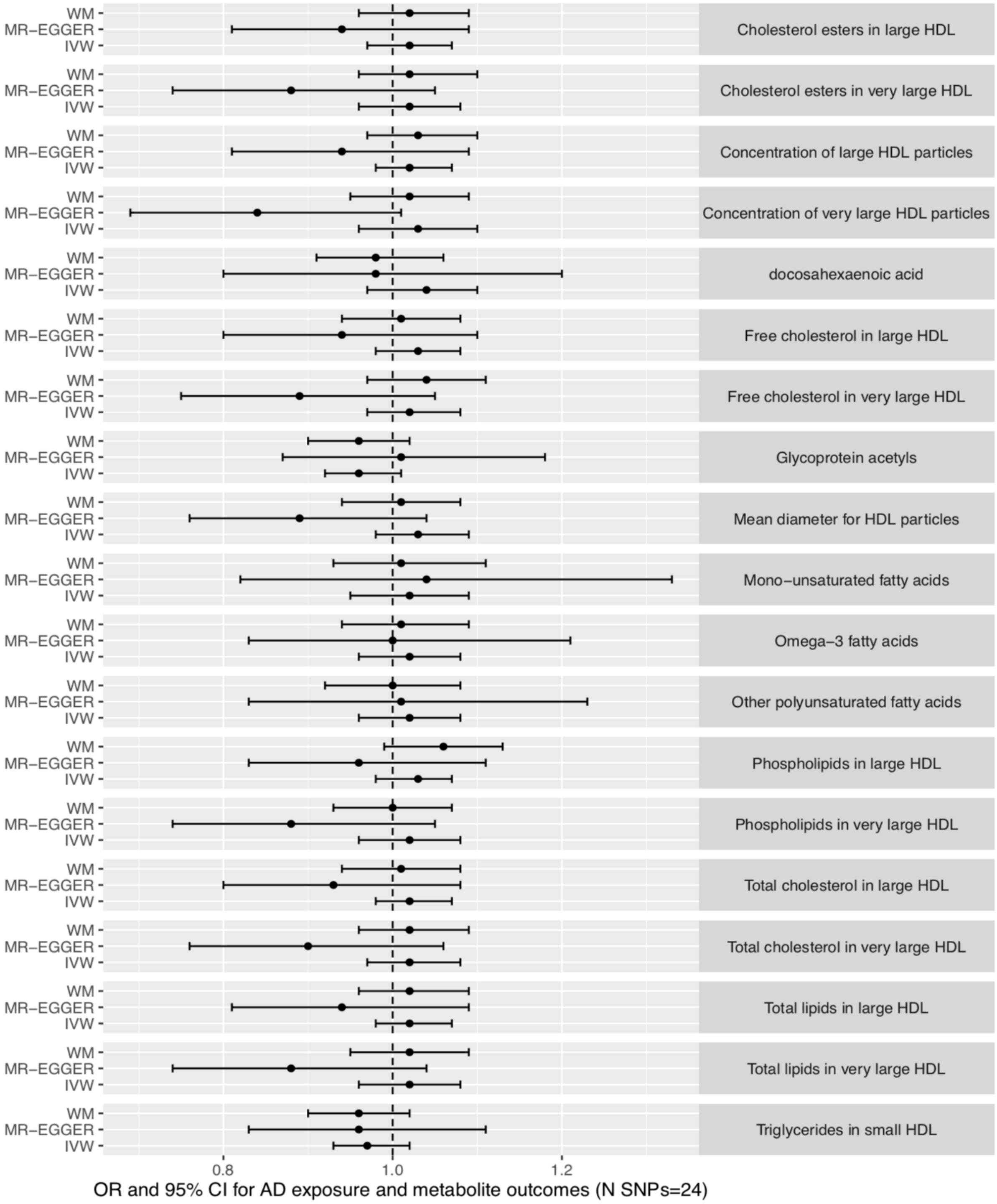

Fig. S2. Results of univariable MR analyses investigating the causal effect of AD on metabolites. Results for inverse variance weighted (IVW), MR-Egger and Weighted Median (WM) for each metabolite are displayed. All results are presented in SD units. Metabolite exposures are confirmed on the right hand side of the chart.

All estimates cross the null, indicating no causal effect in the direction of AD to metabolites

Fig. S3a.

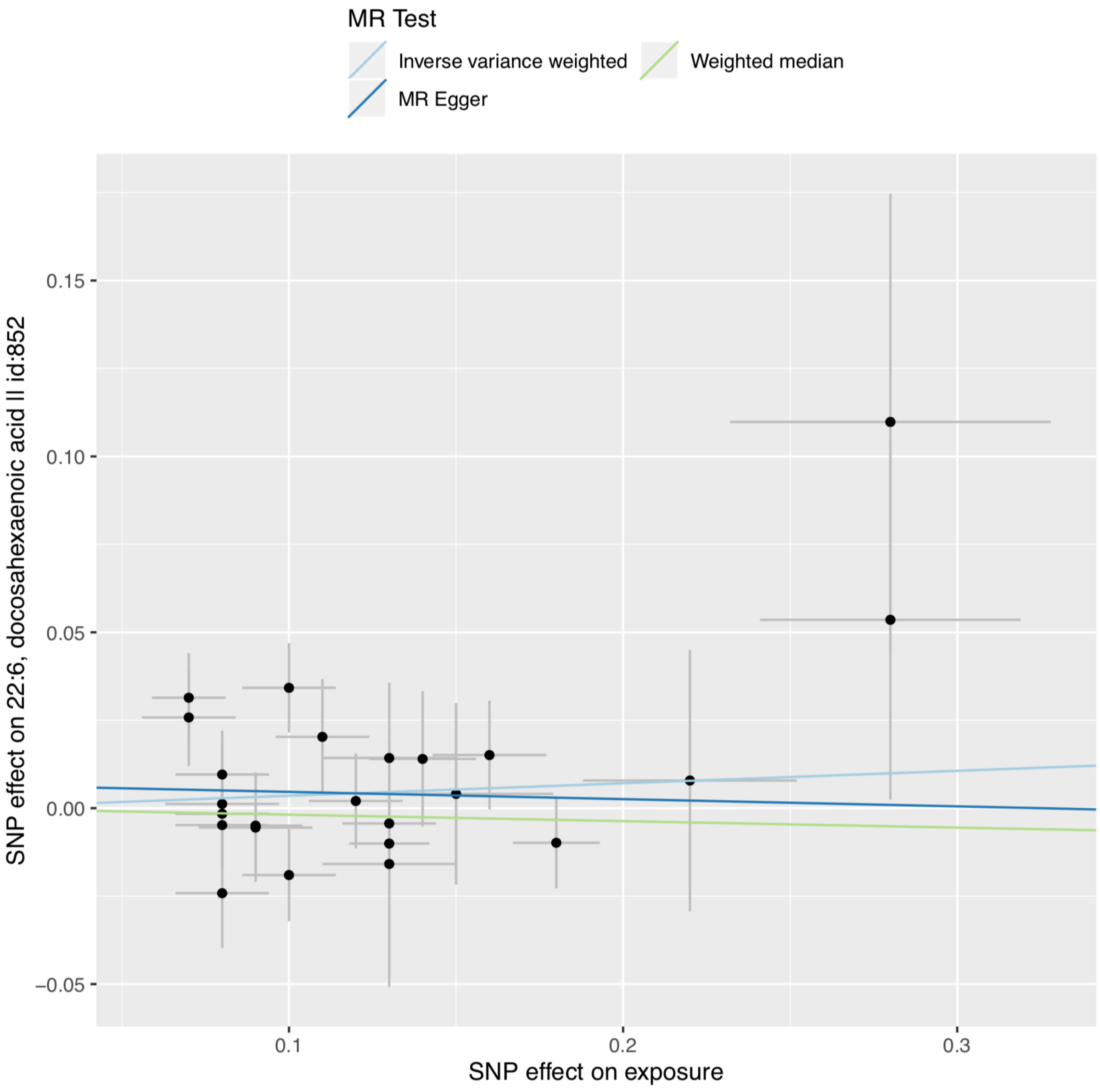

Fig. S3b.

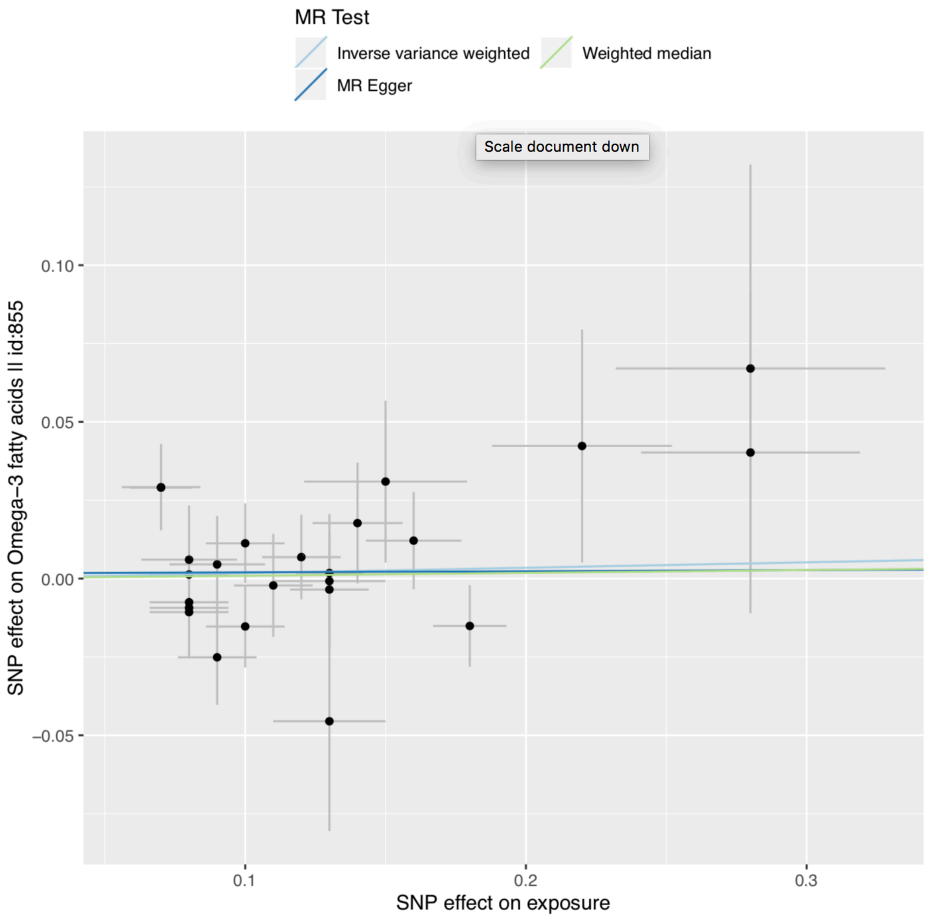

Fig. S3c.

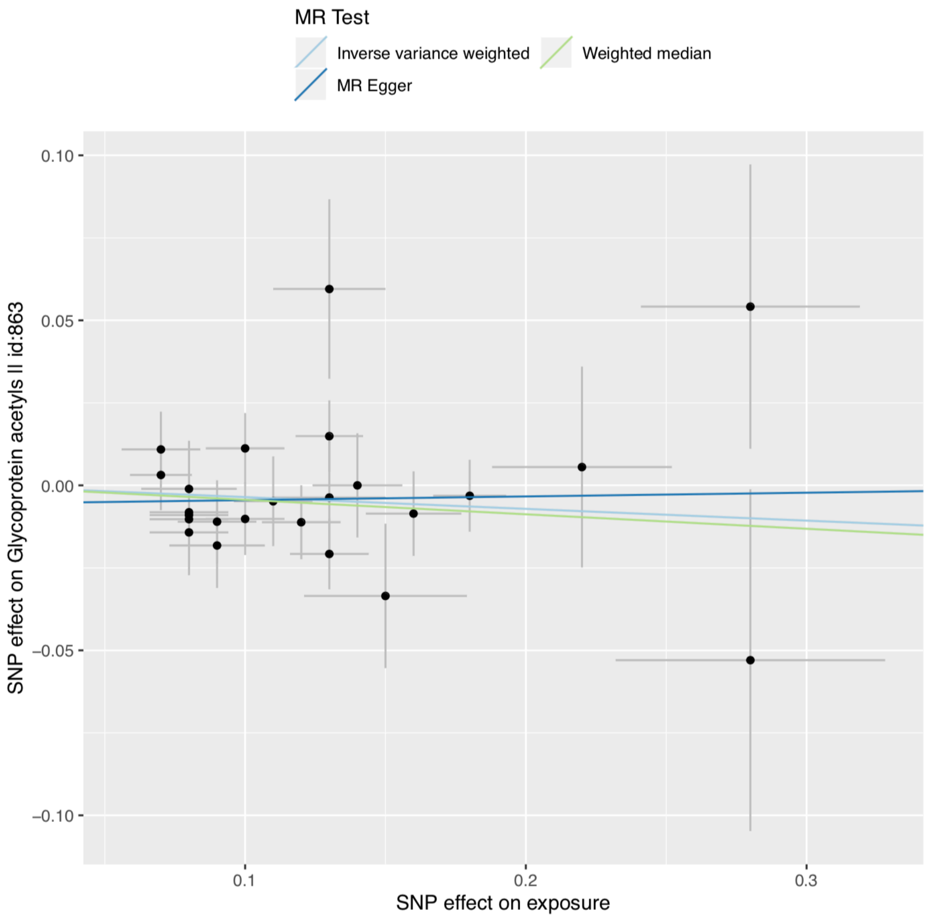

Fig. S3d.

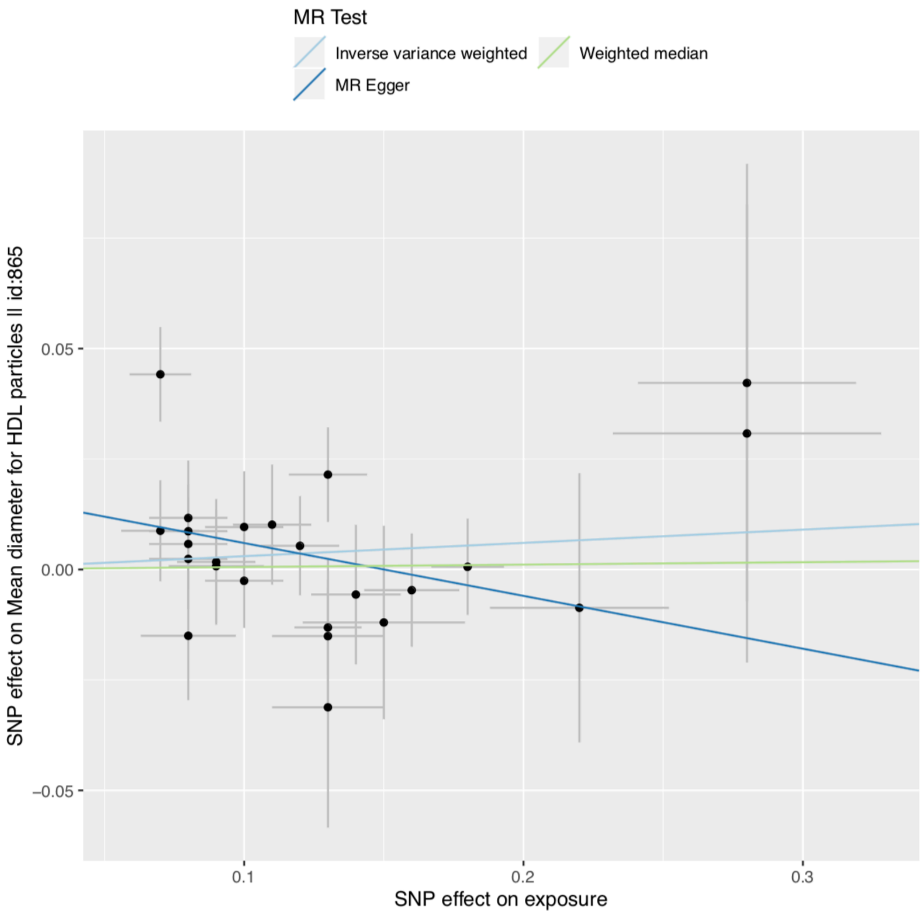

Fig. S3e.

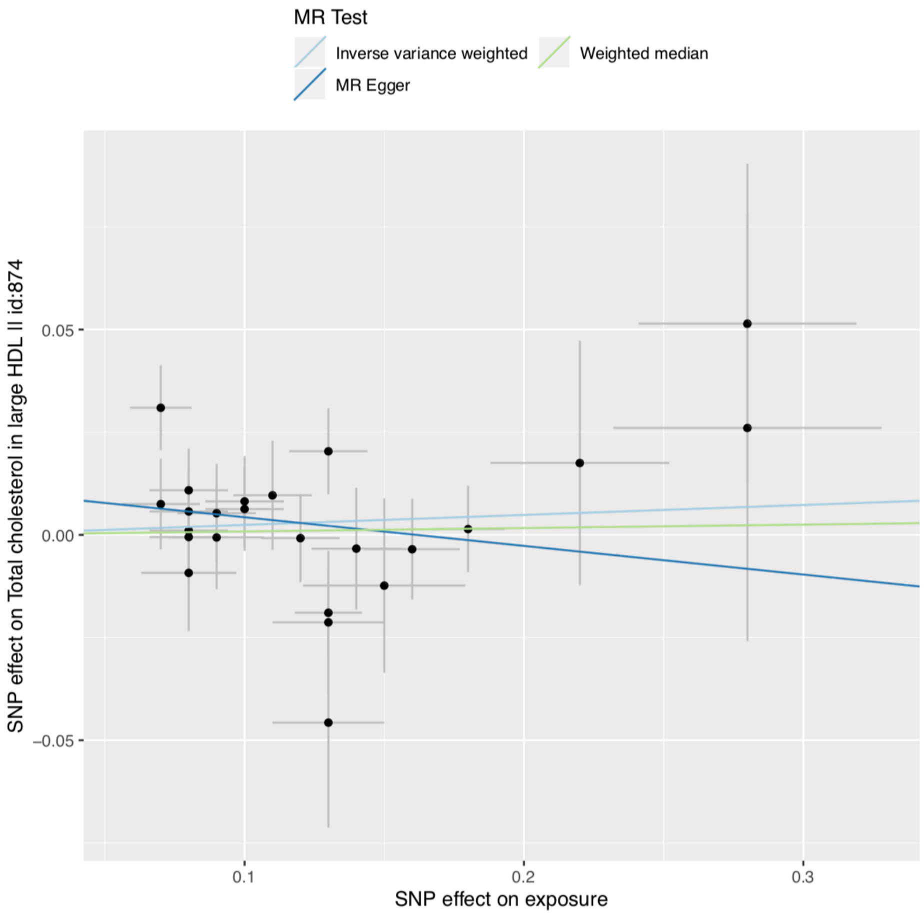

Fig. S3f.

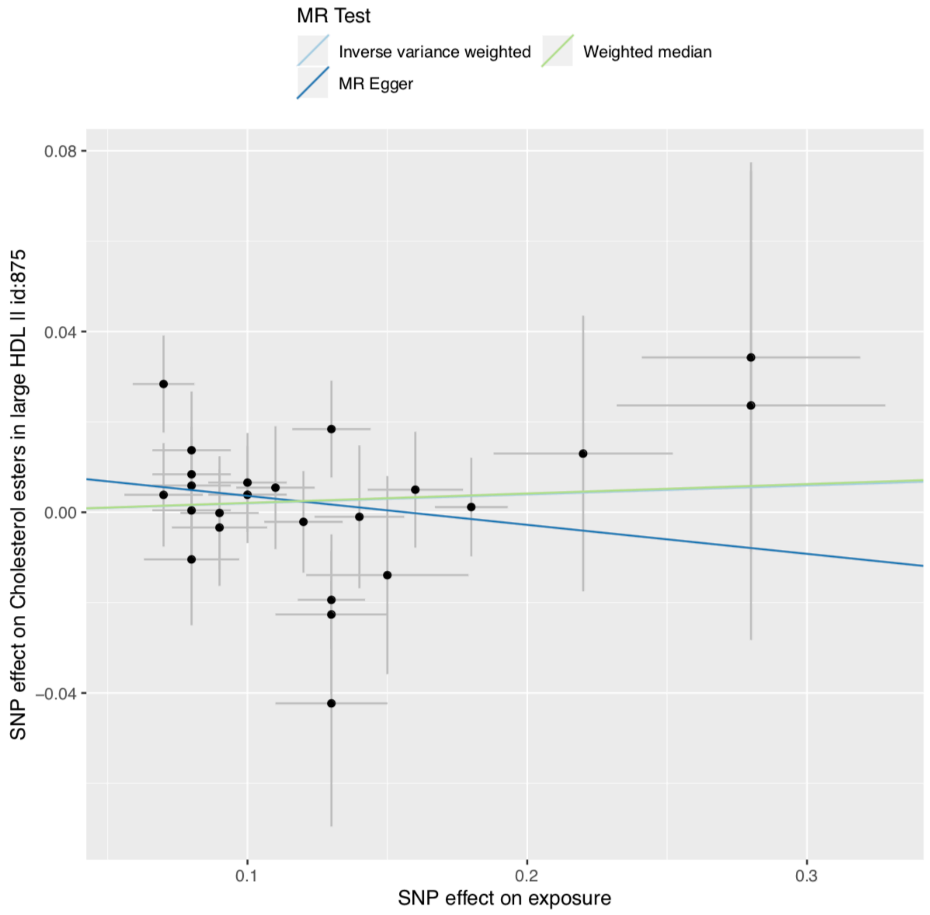

Fig. S3g.

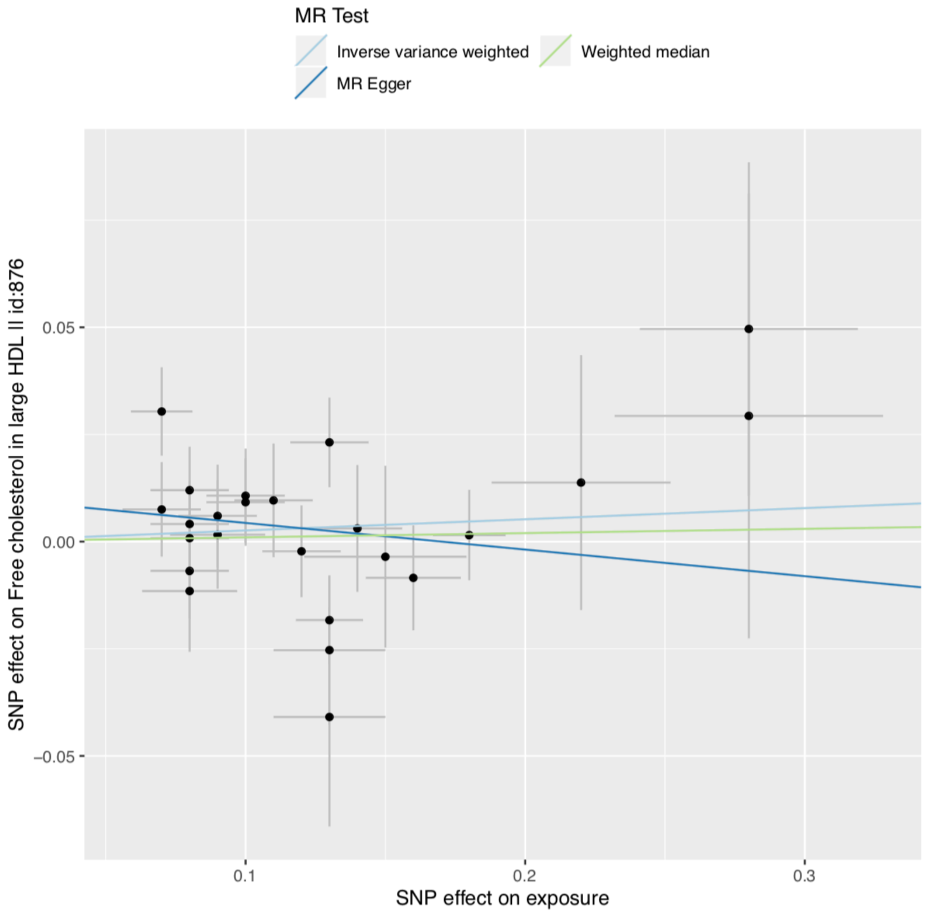

Fig. S3h.

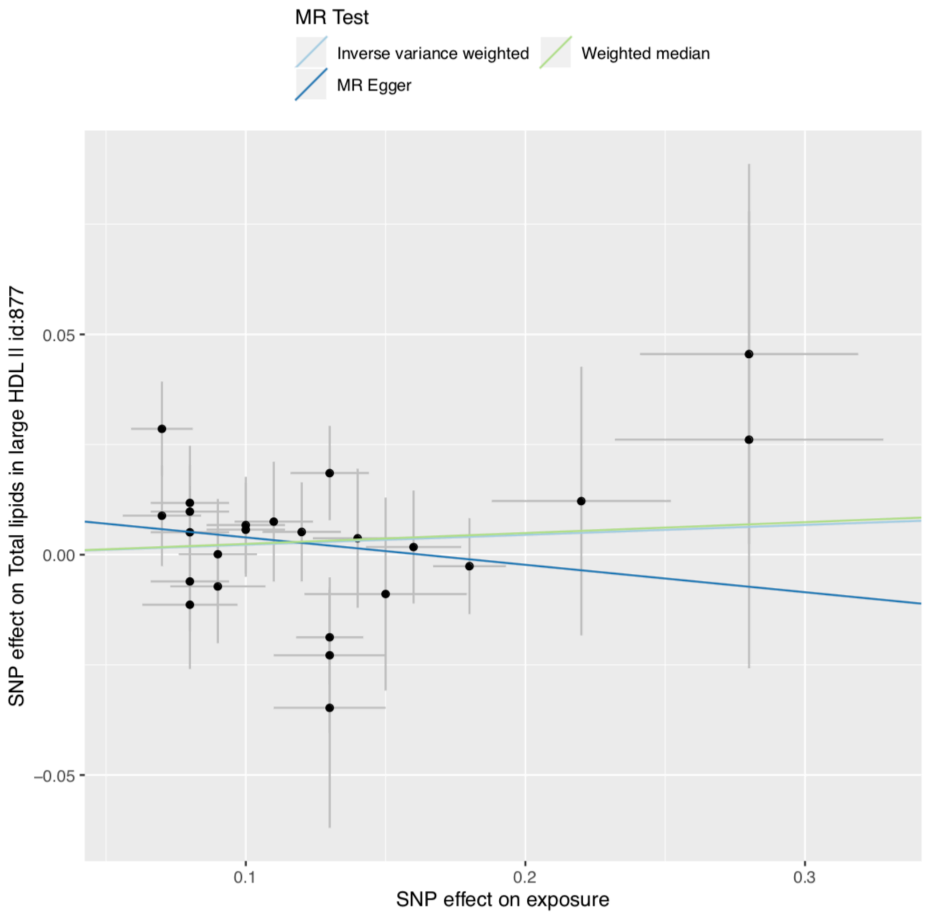

Fig. S3i.

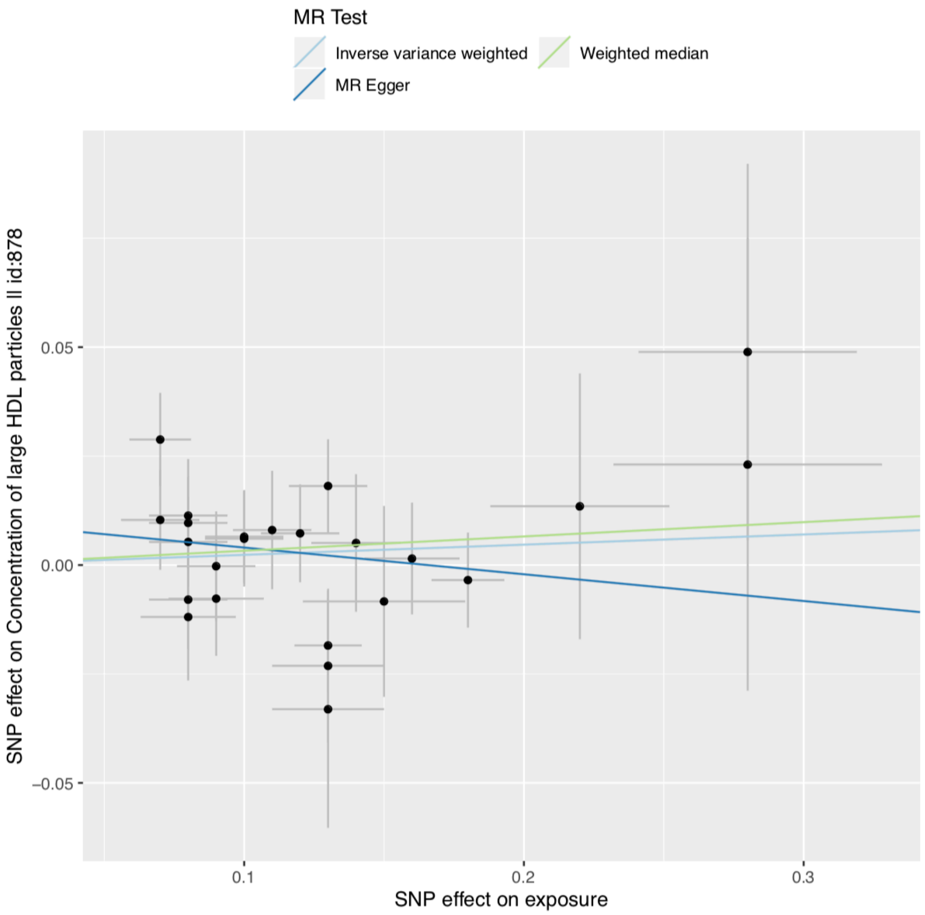

Fig. S3j.

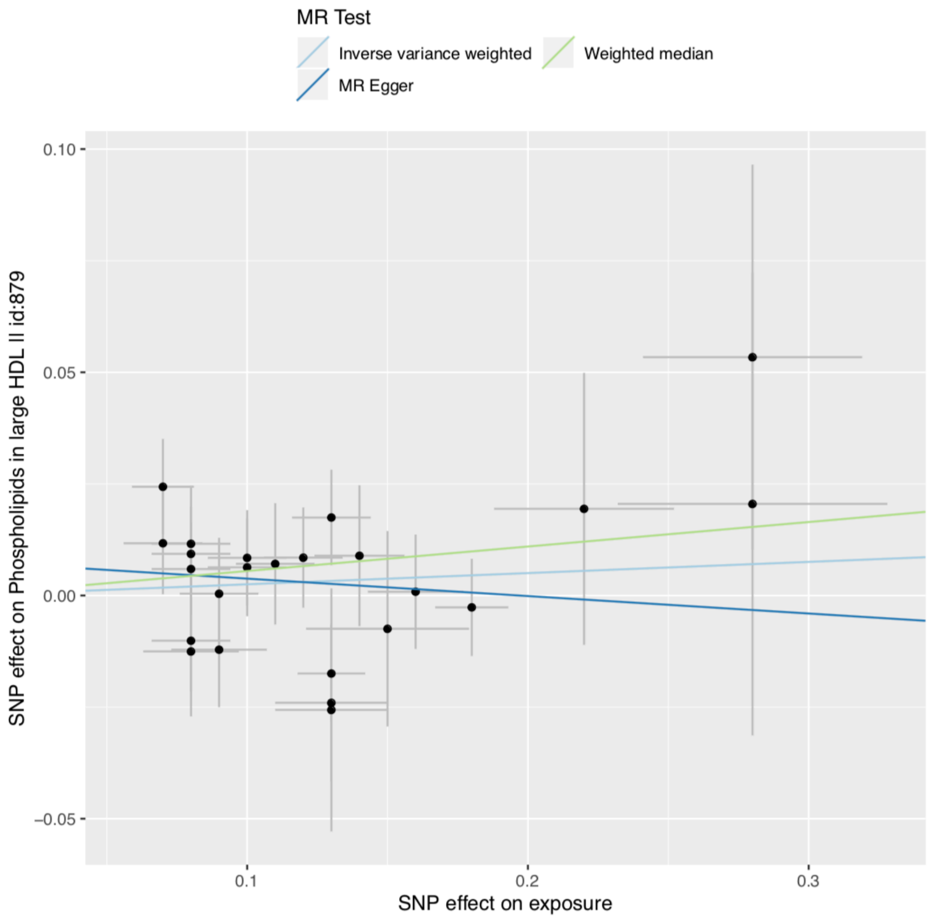

Fig. S3k.

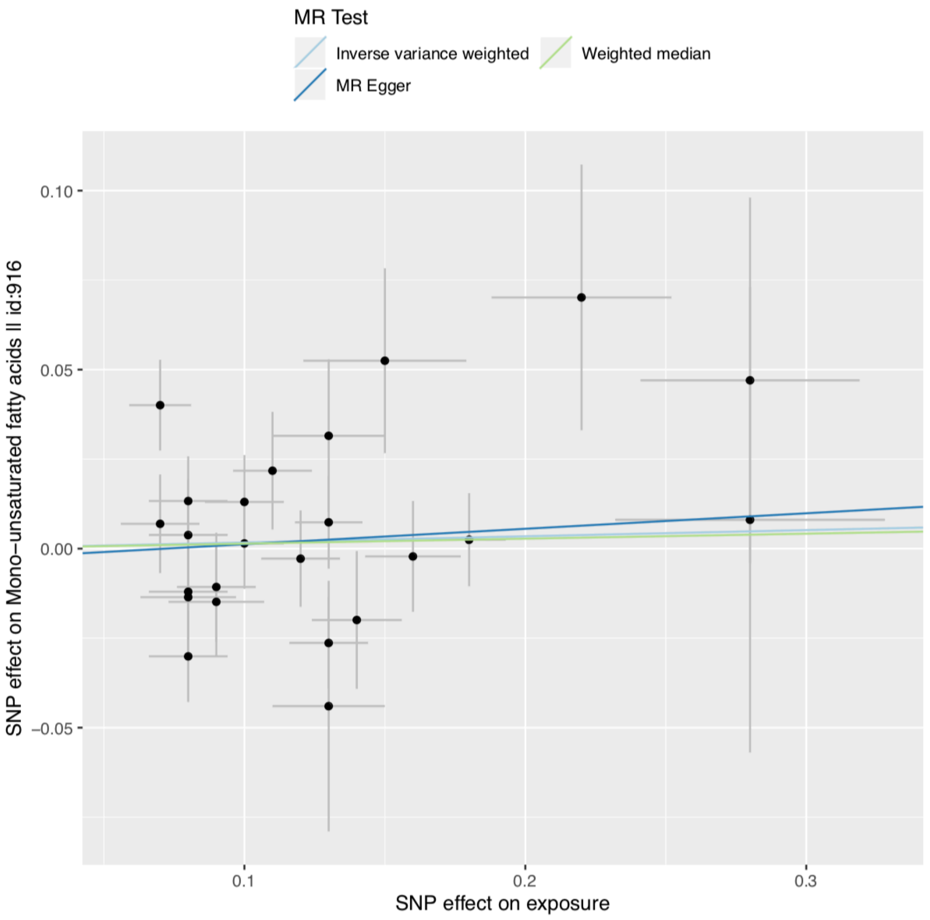

Fig. S3l.

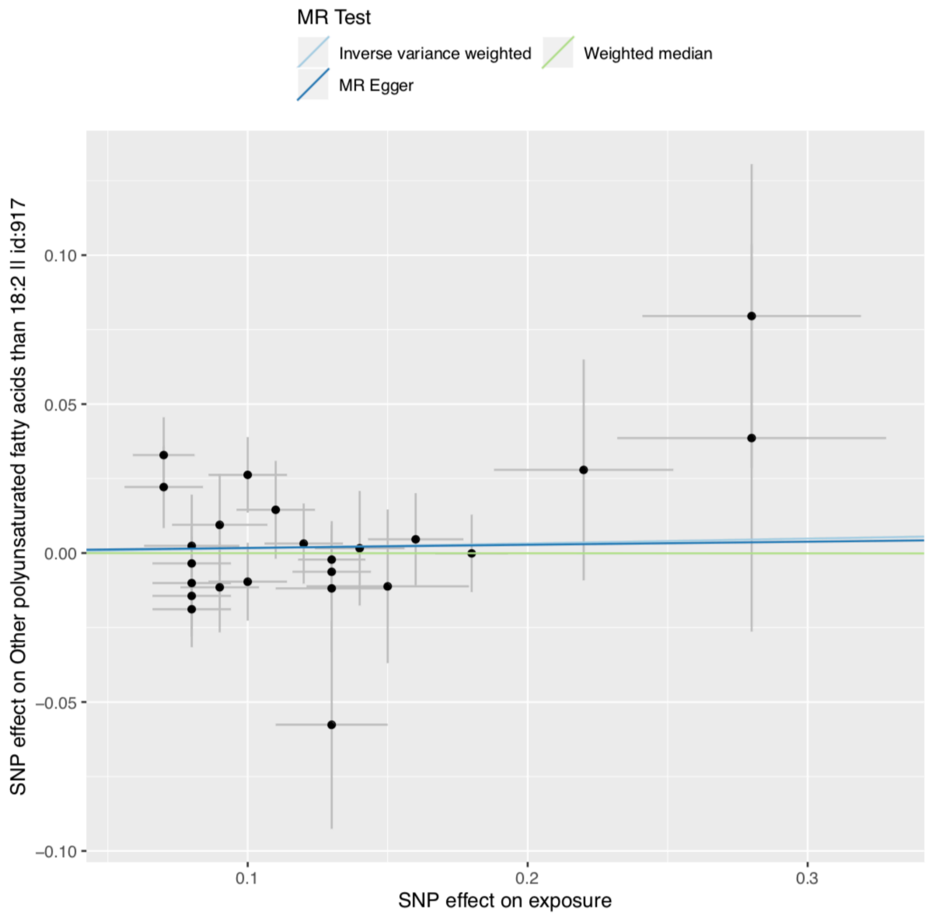

Fig. S3m.

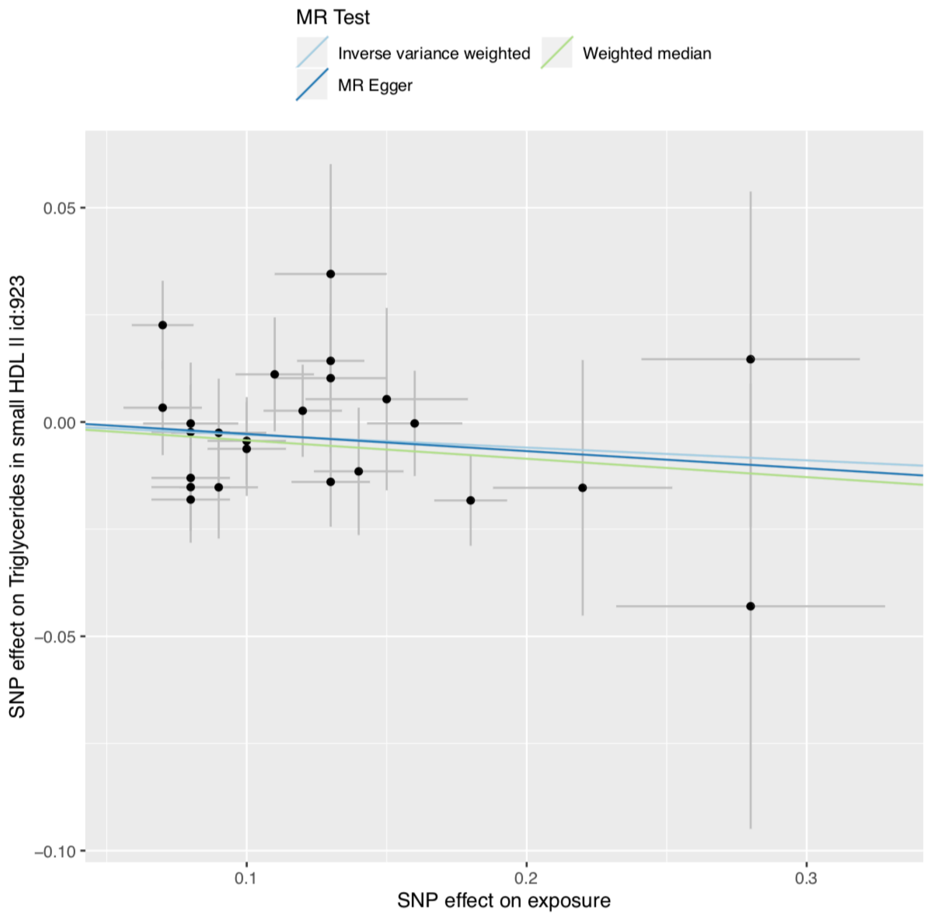

Fig. S3n.

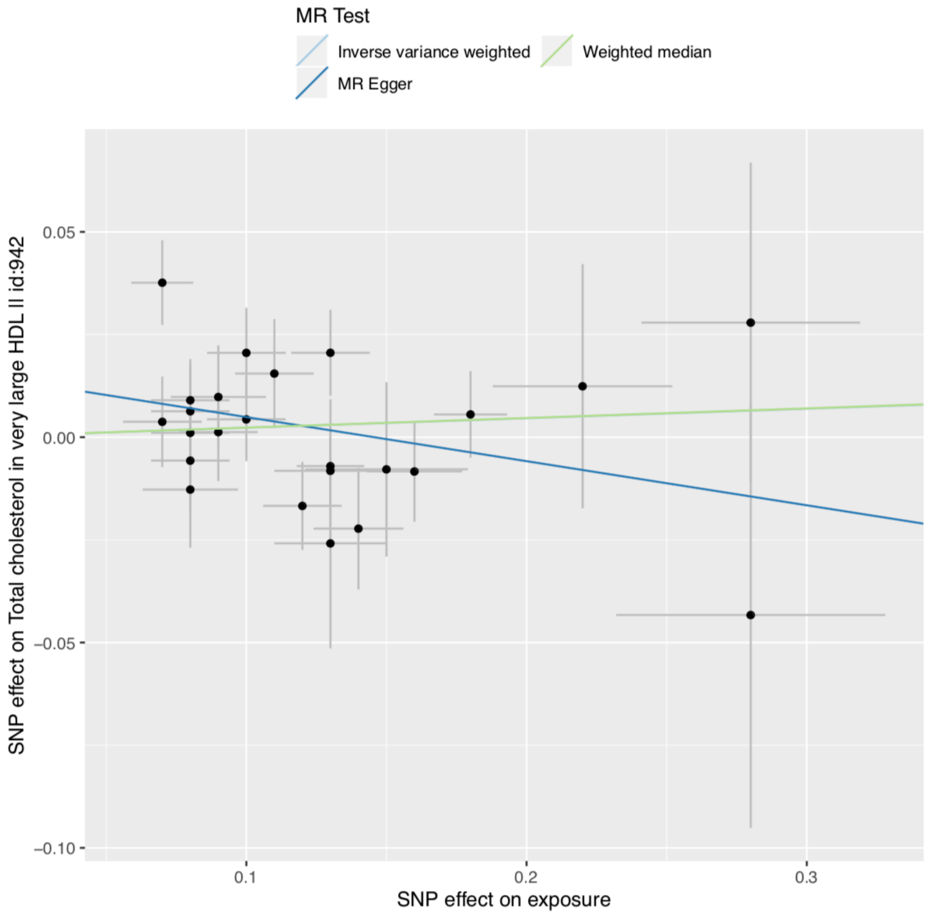

Fig. S3o.

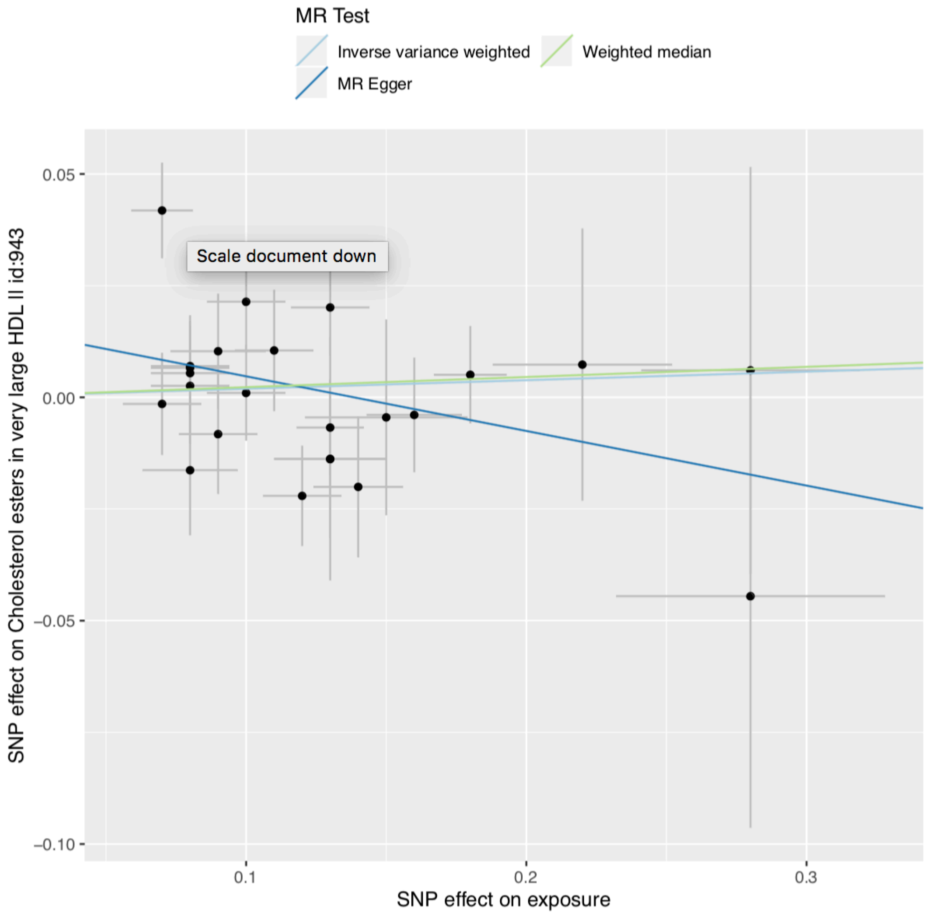

Fig. S3p.

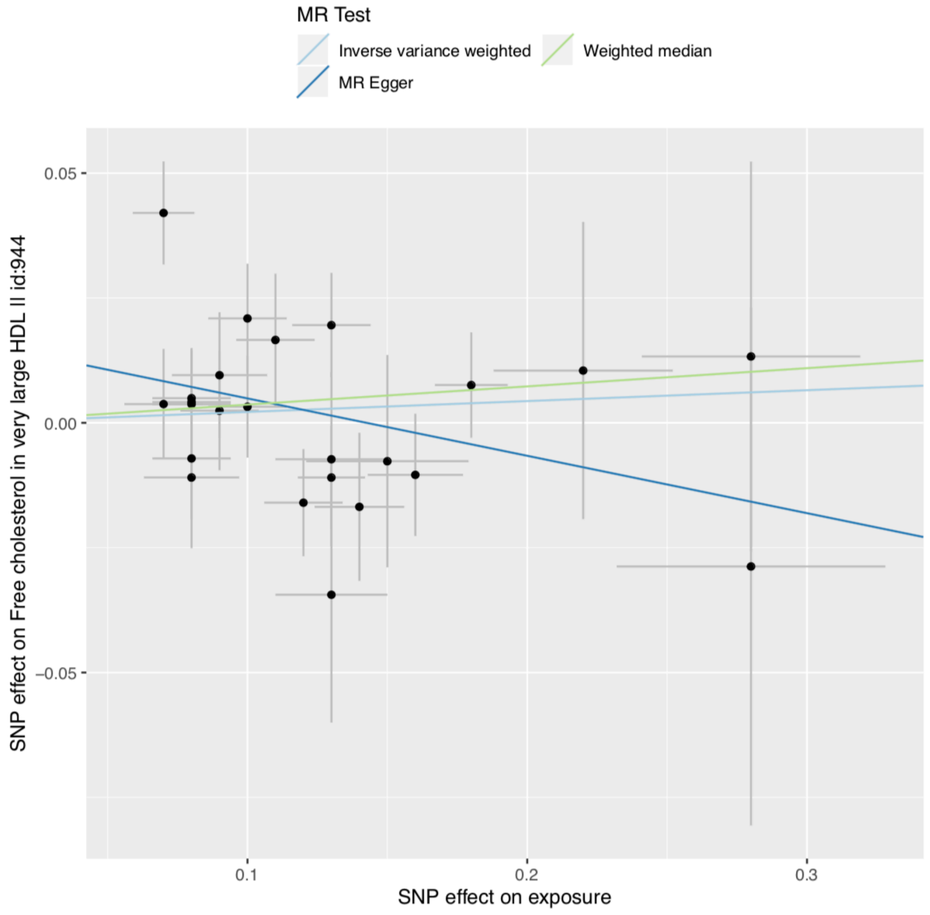

Fig. S3q.

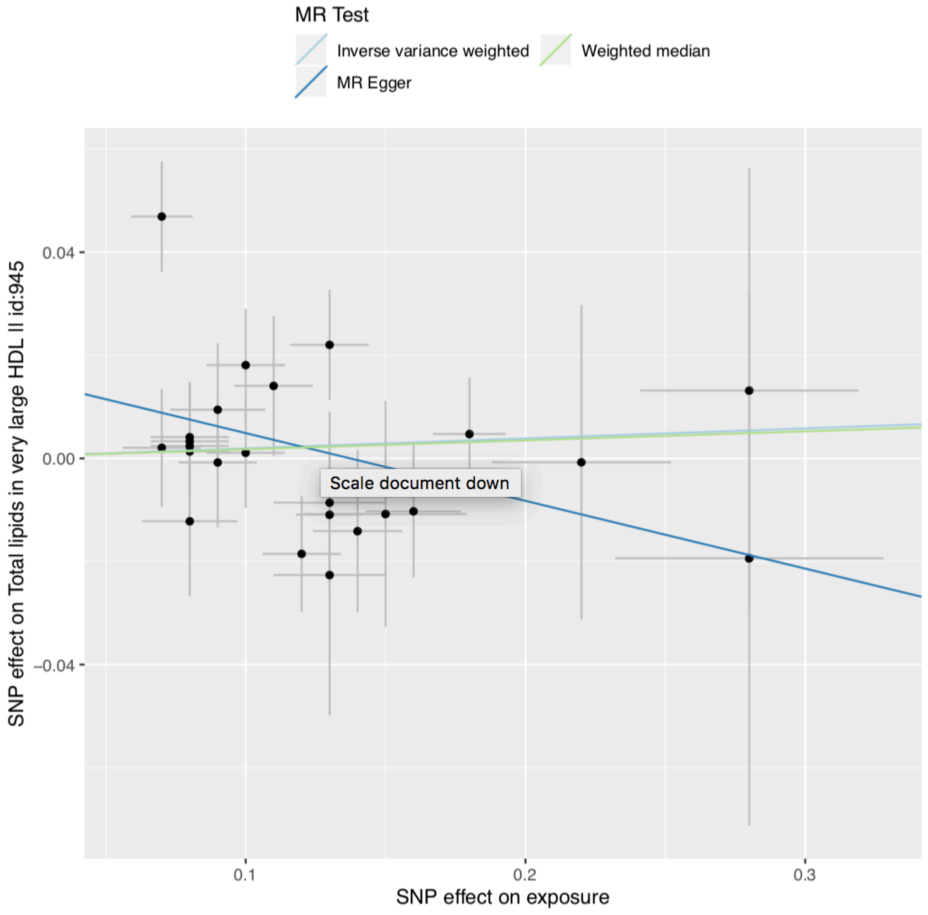

Fig. S3r.

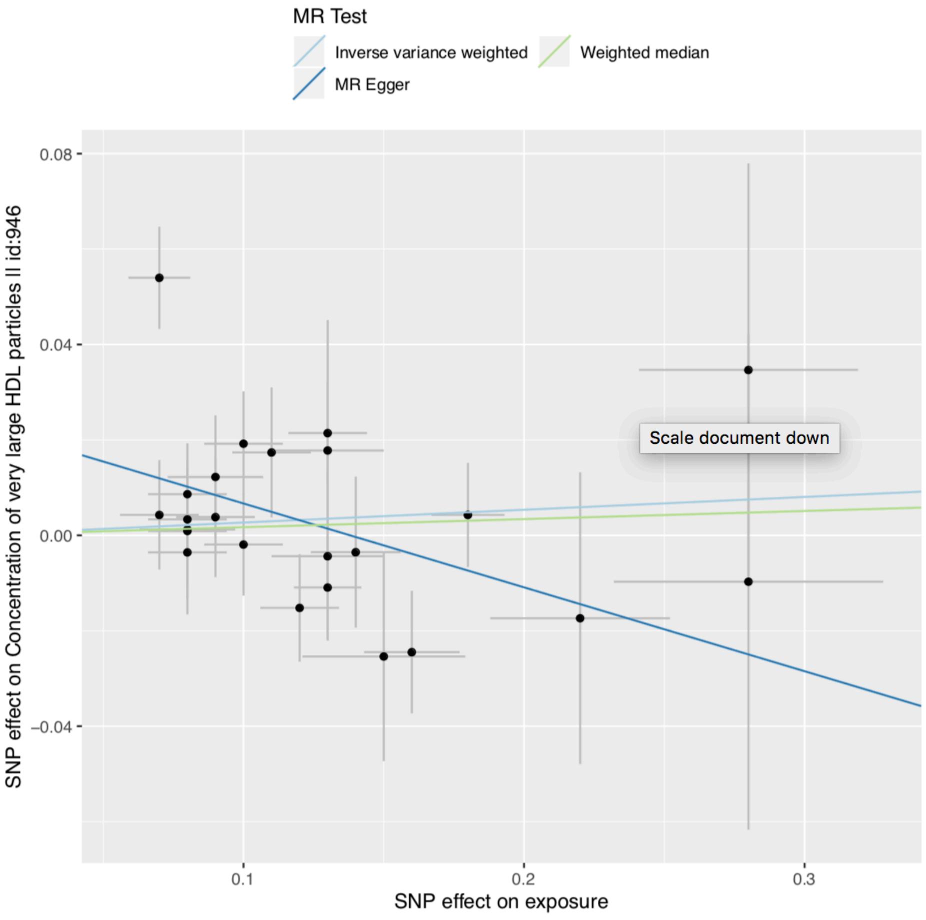

Fig. S3s.

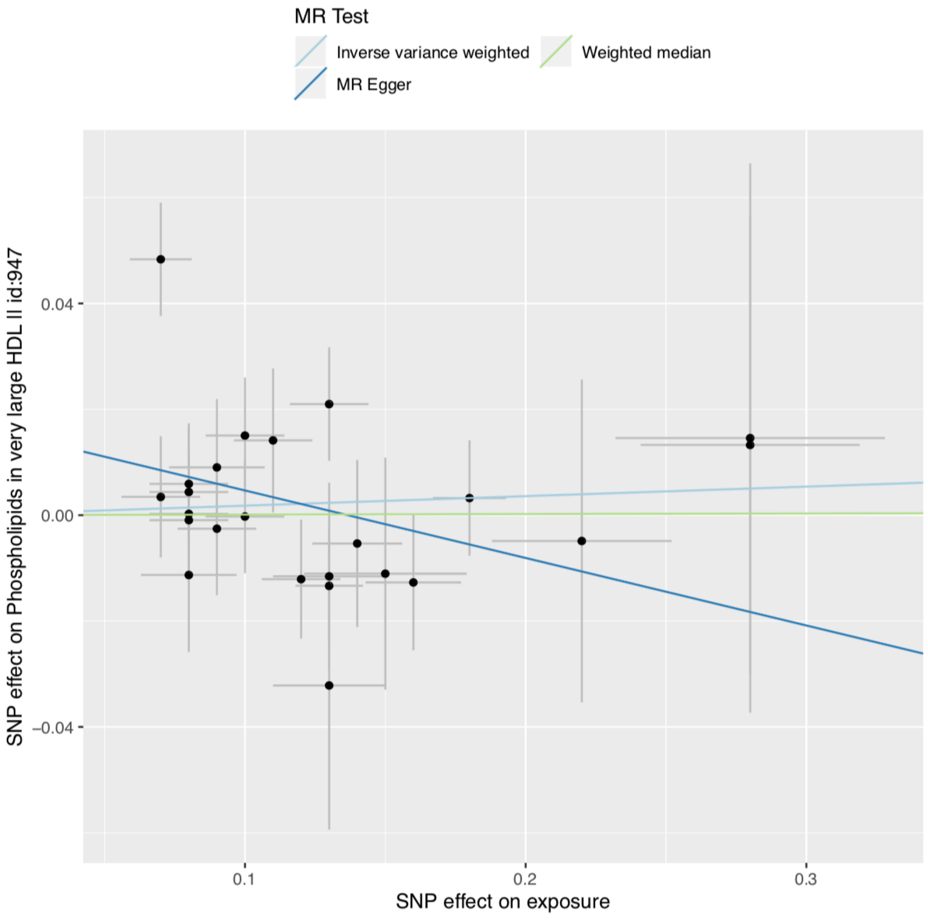

Fig. S3a-S3s. Scatterplots of the gene-metabolite versus gene-AD associations for each AD-metabolite pair when AD is the exposure and each metabolite the outcome following univariable MR analyses.

Each point in the scatter plot represents an instrumental SNP and different regression lines represents the different MR methods used.

Fig. S4a.

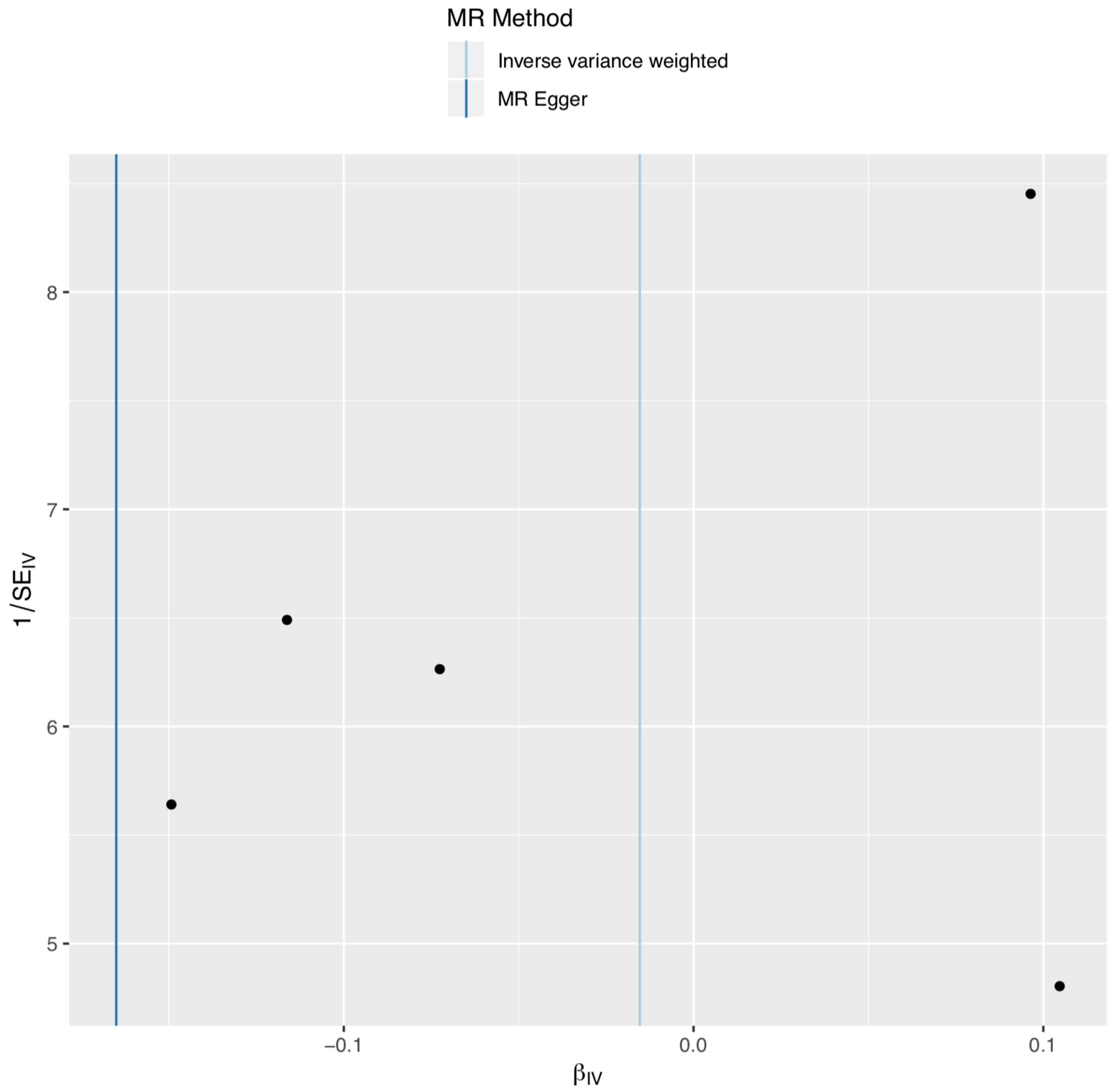
MR Method
Inverse variance weighted
MR Egger
8
7
6
5
1/SE$_{IV}$
−0.1
0.0
0.1
β_{IV}

Fig. S4b.

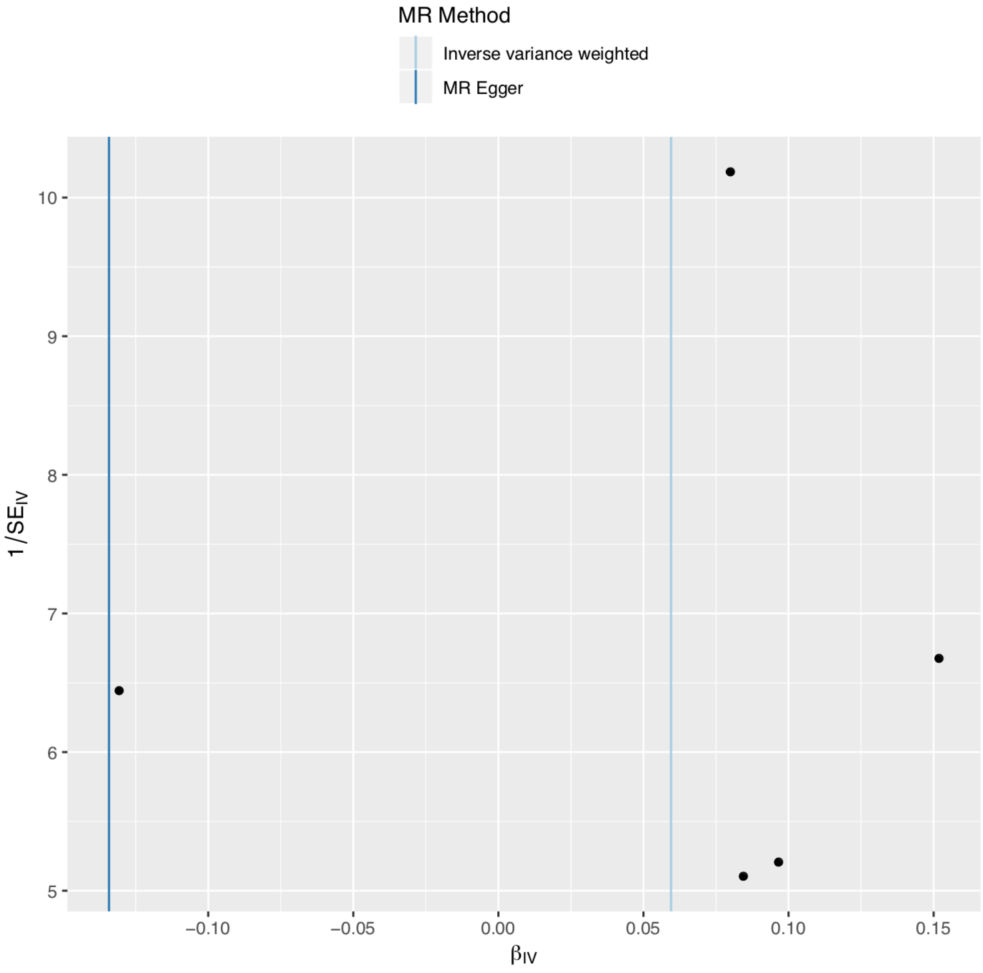

Fig. S4c.

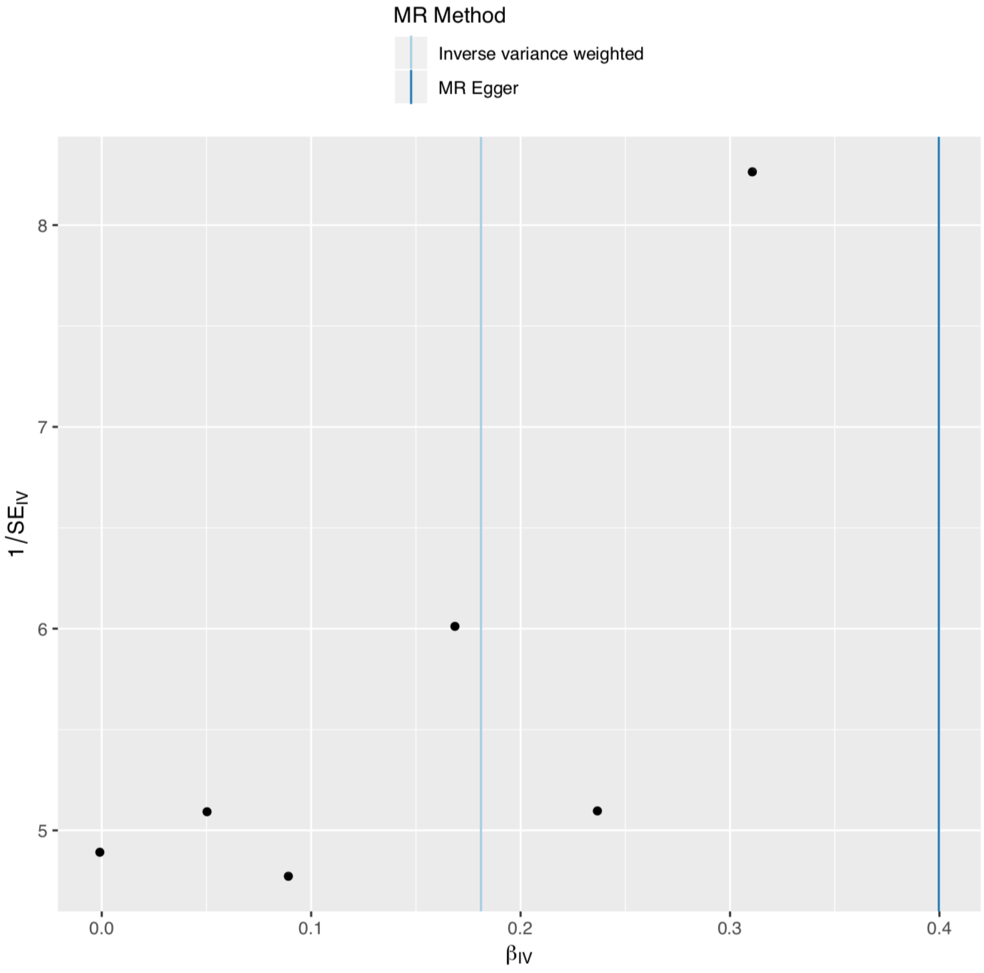

Fig. S4d.

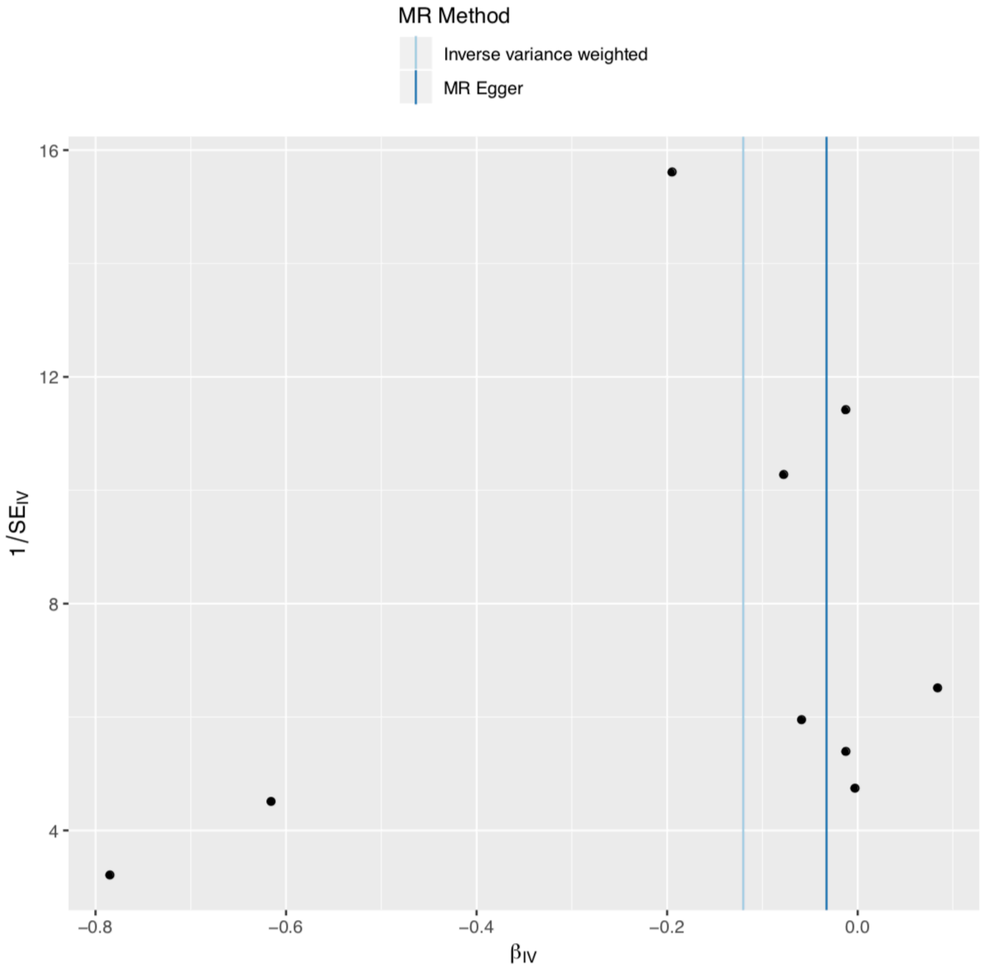

Fig. S4e.

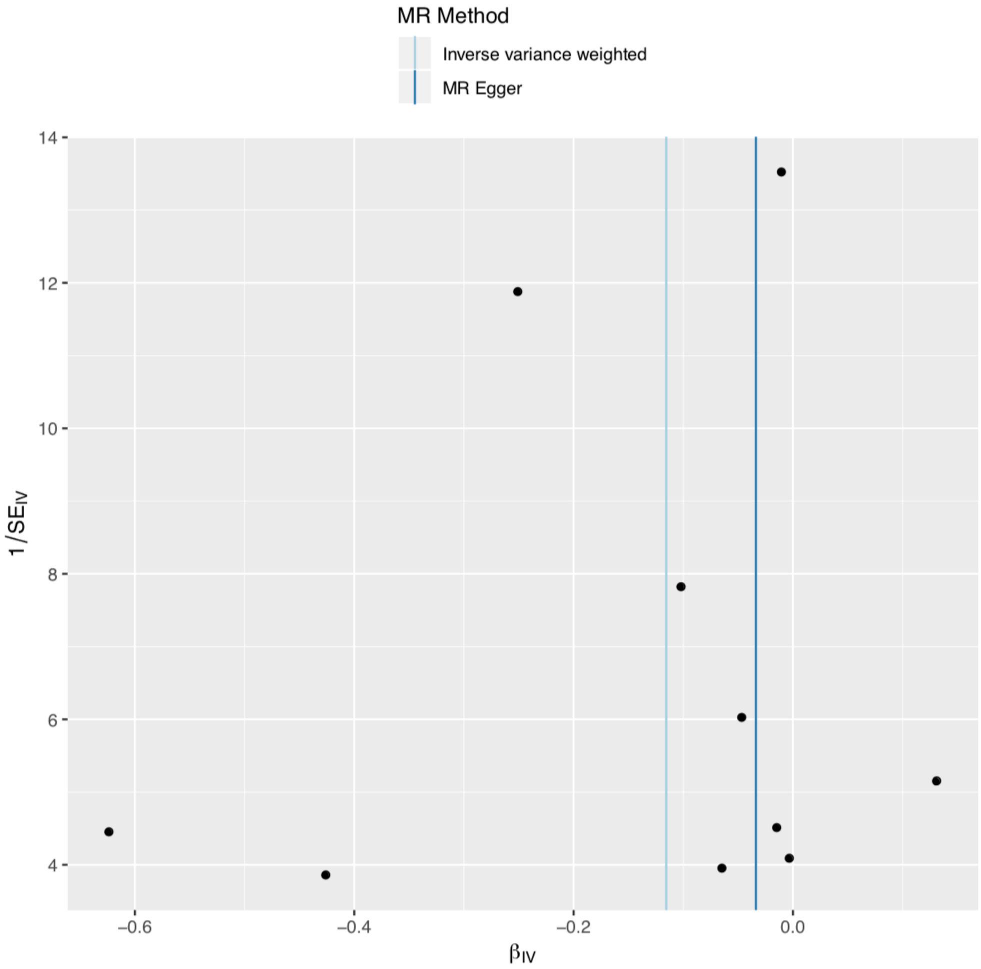

Fig. S4f.

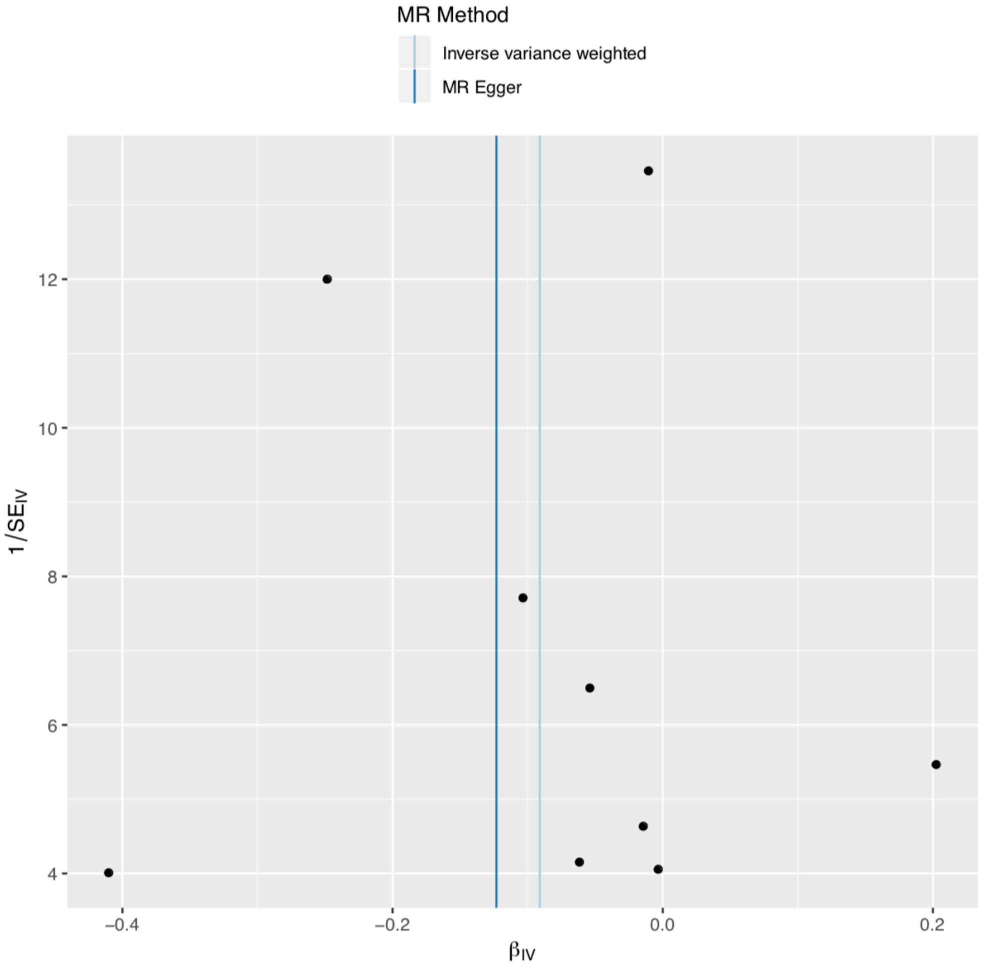

Fig. S4g.

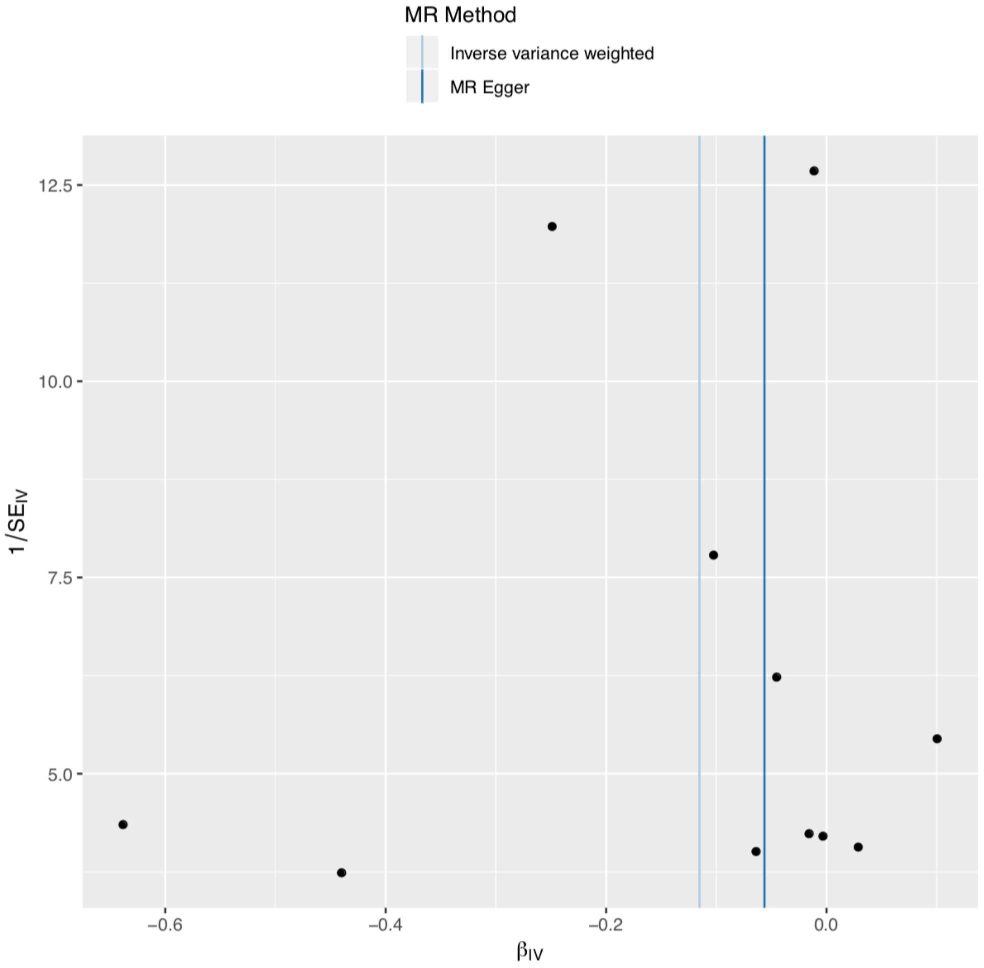

Fig. S4h.

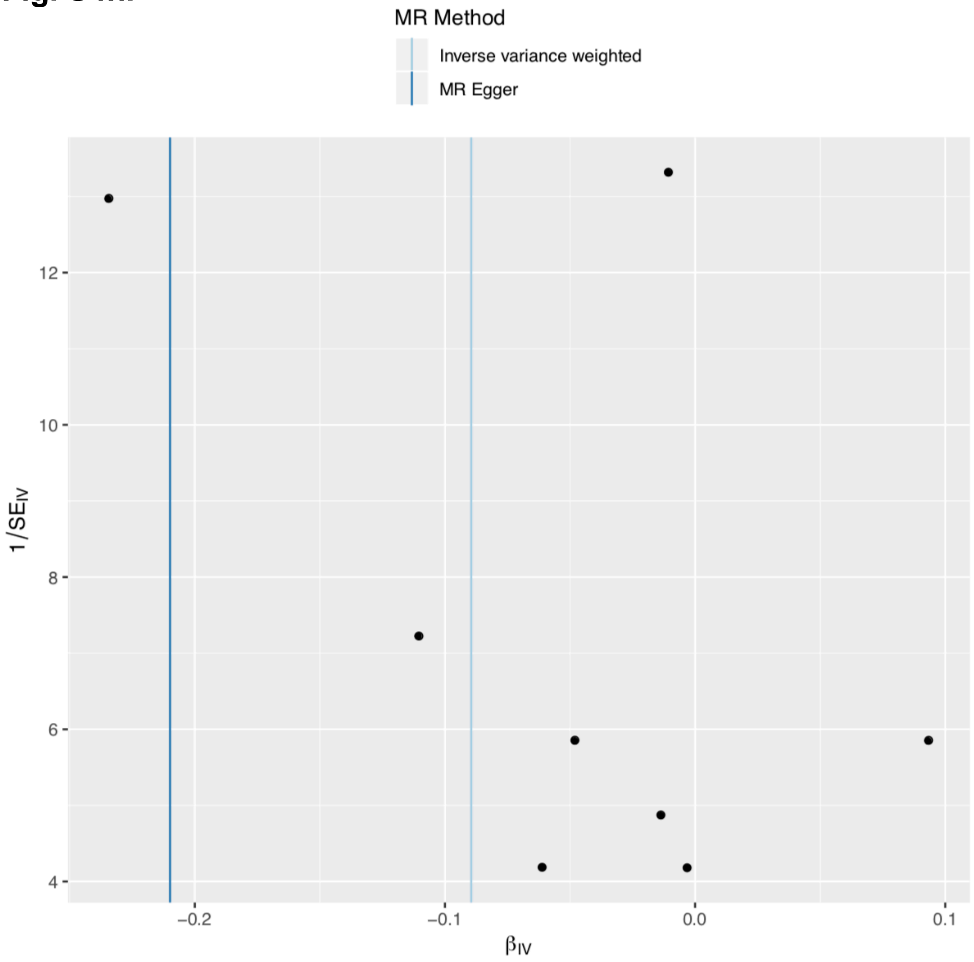

Fig. S4i.

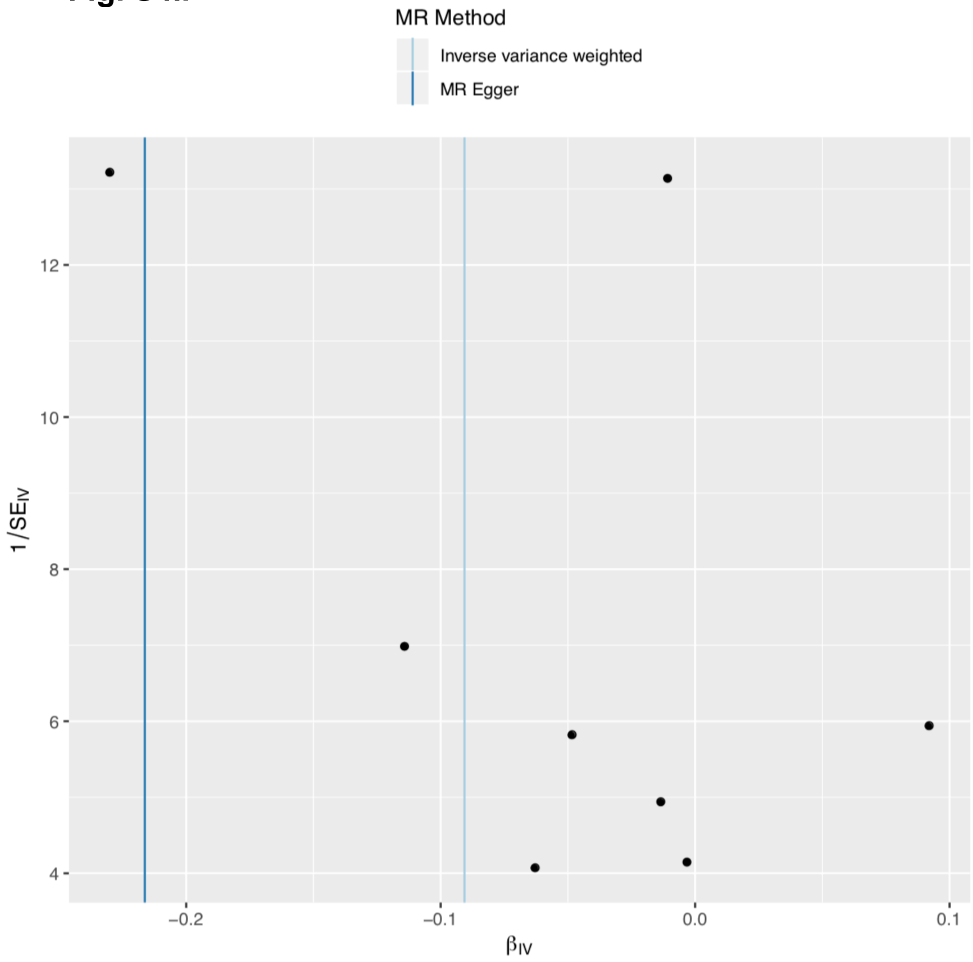

Fig. S4j.

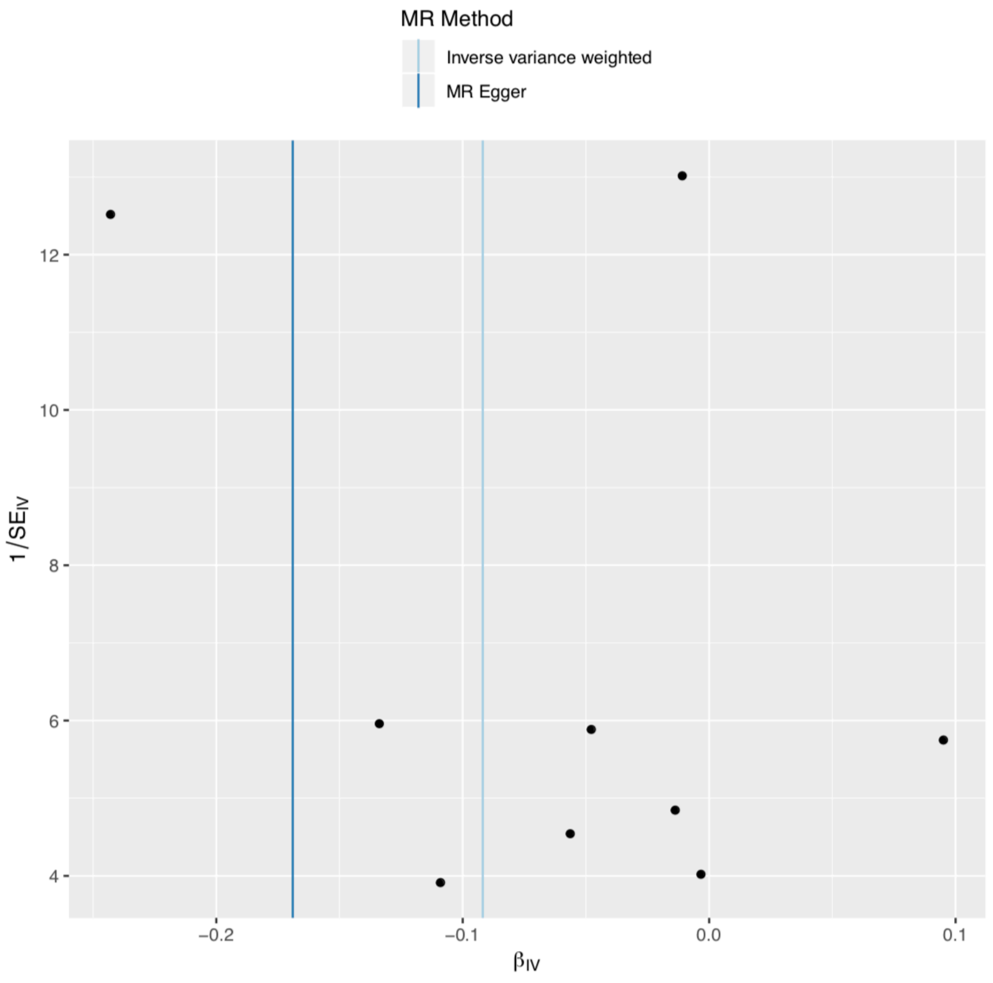

Fig. S4k.

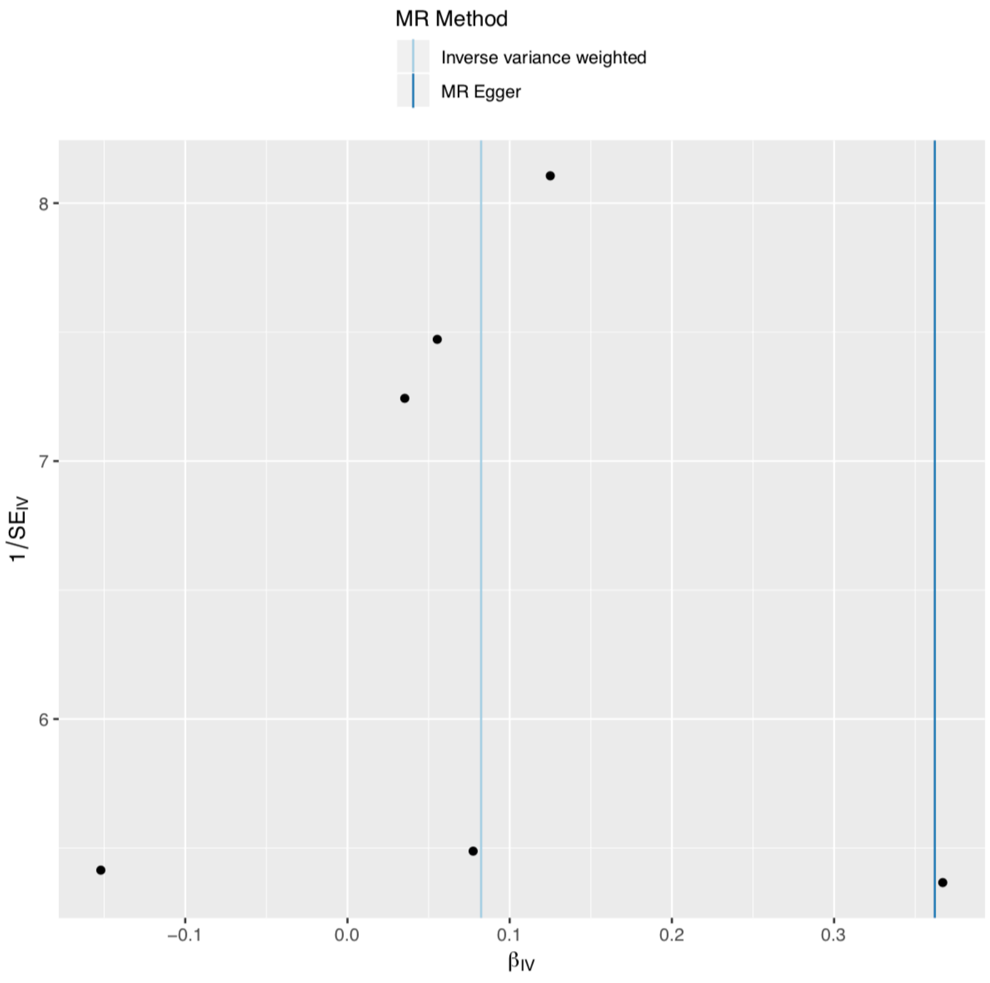

Fig. S4l.

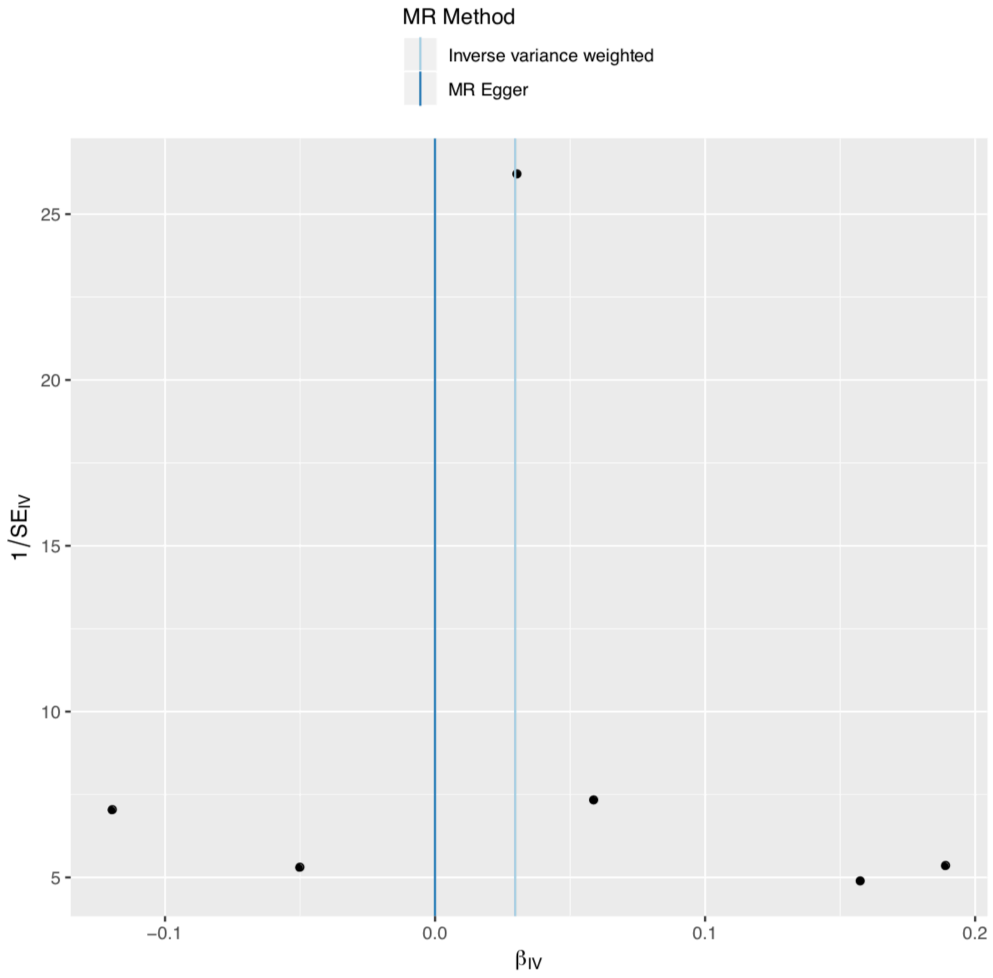

Fig. S4m.

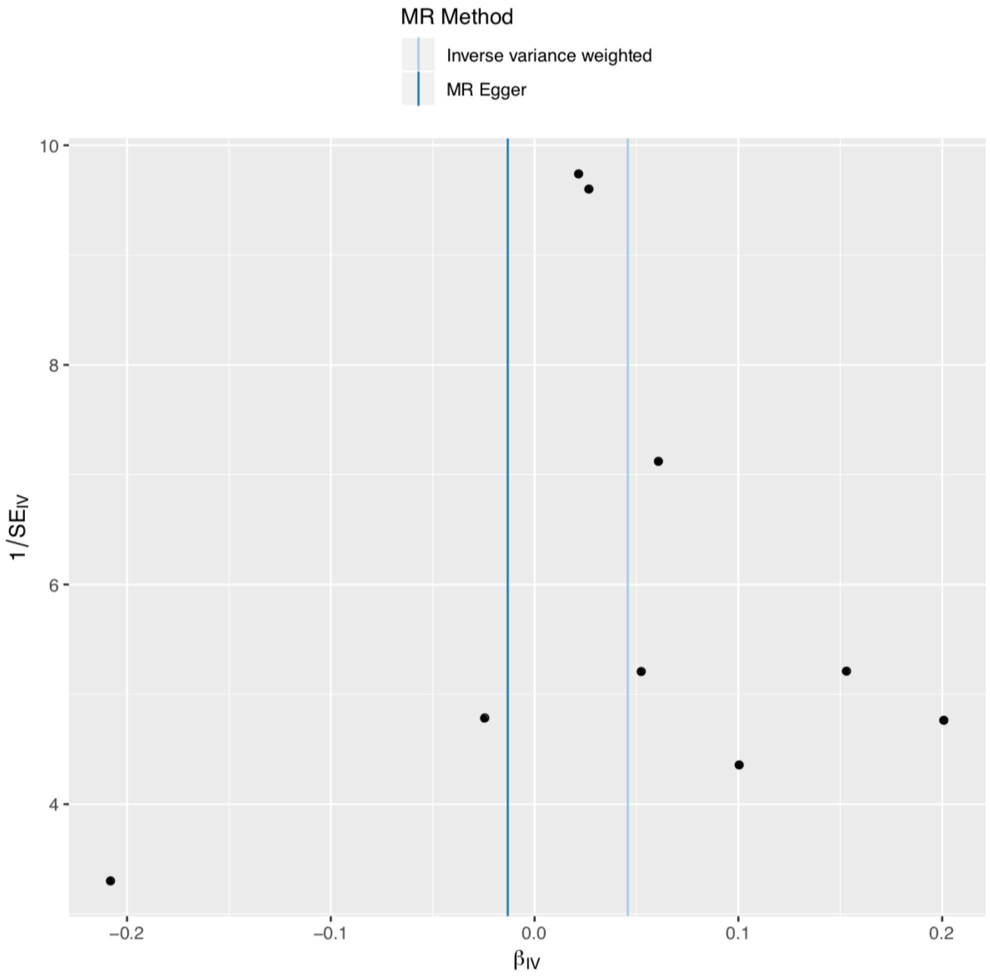

Fig. S4n.

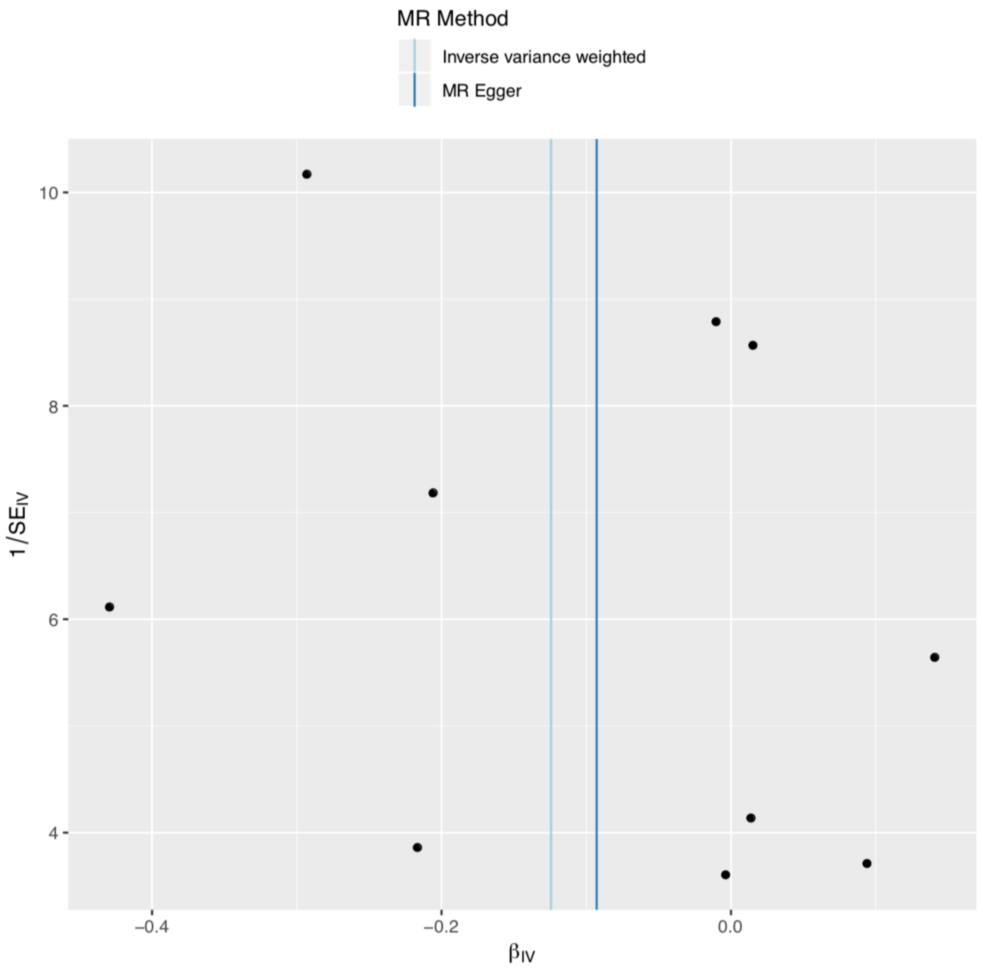

Fig. S4o.

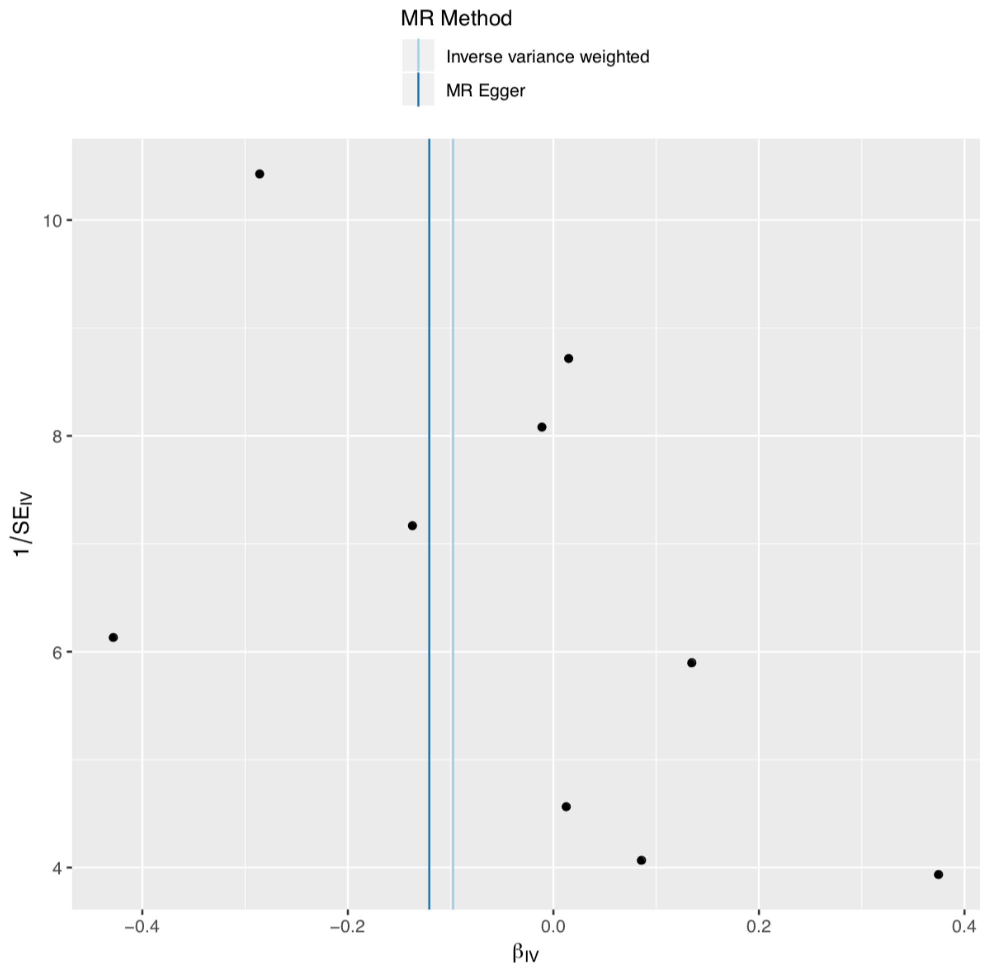

Fig. S4p.

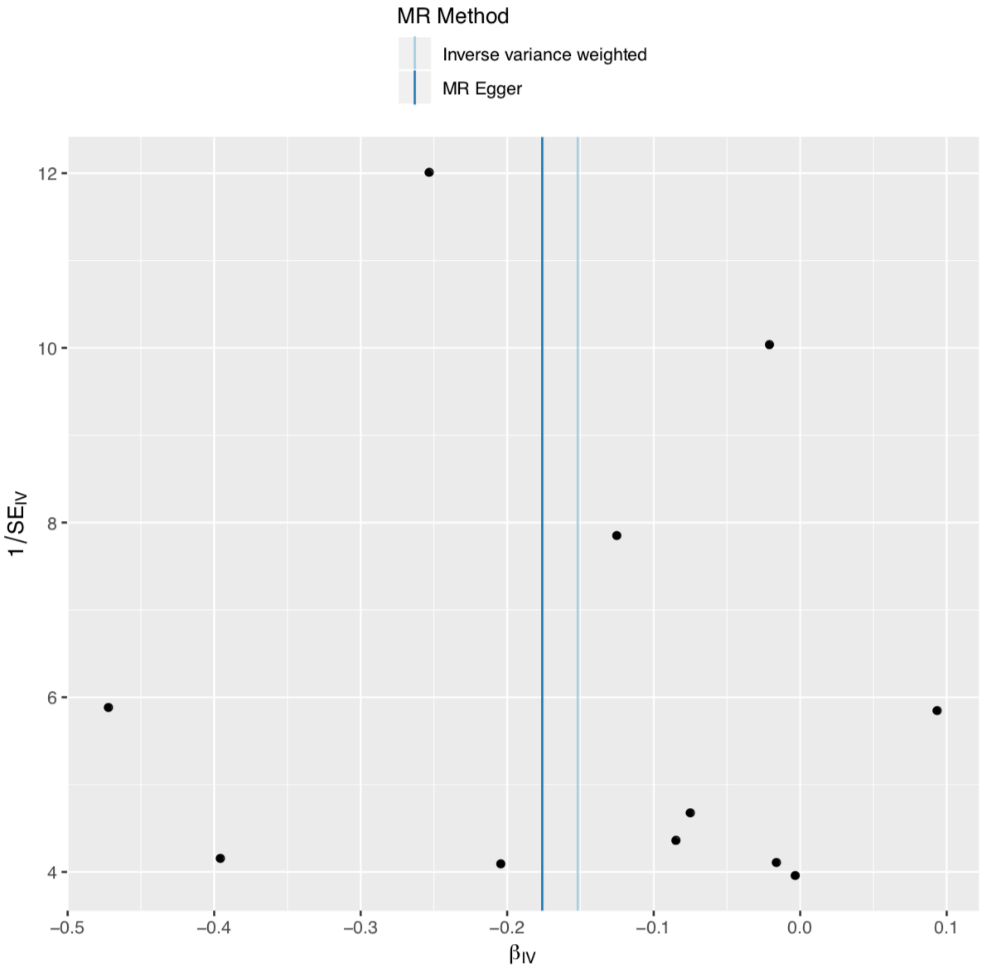

Fig. S4q.

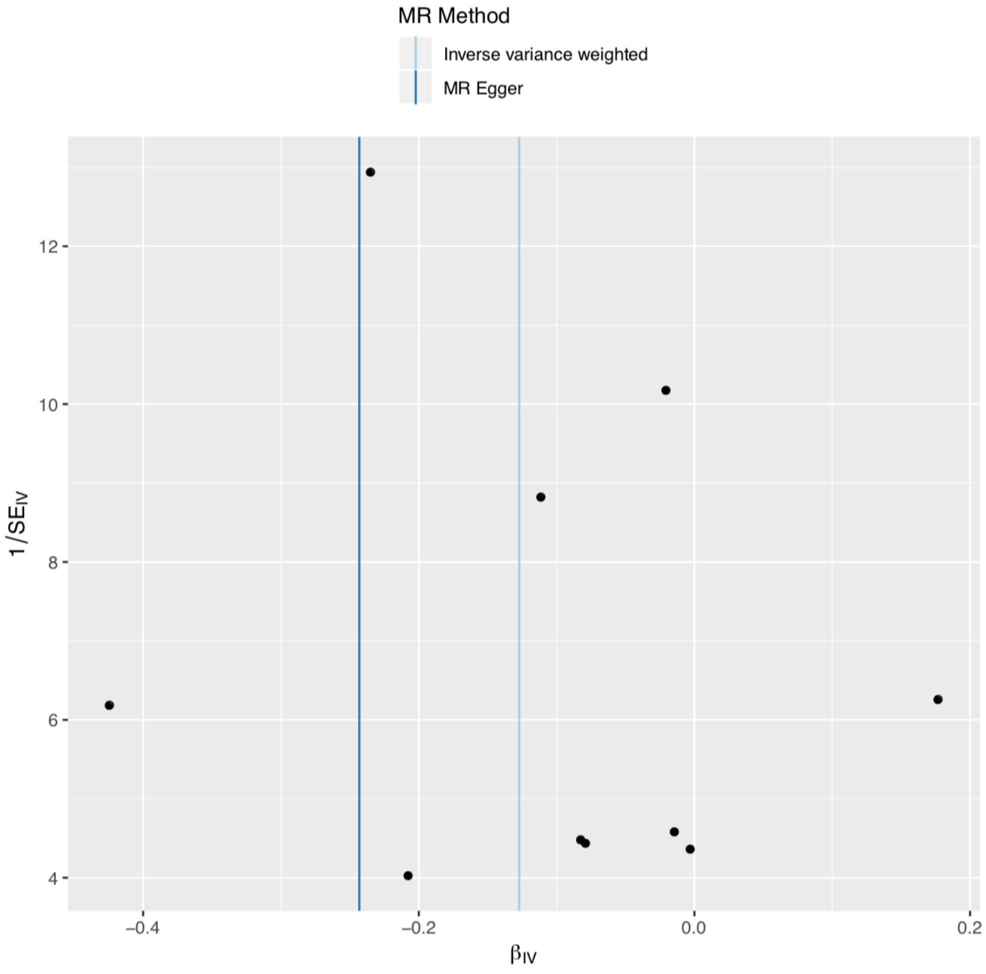

Fig. S4r.

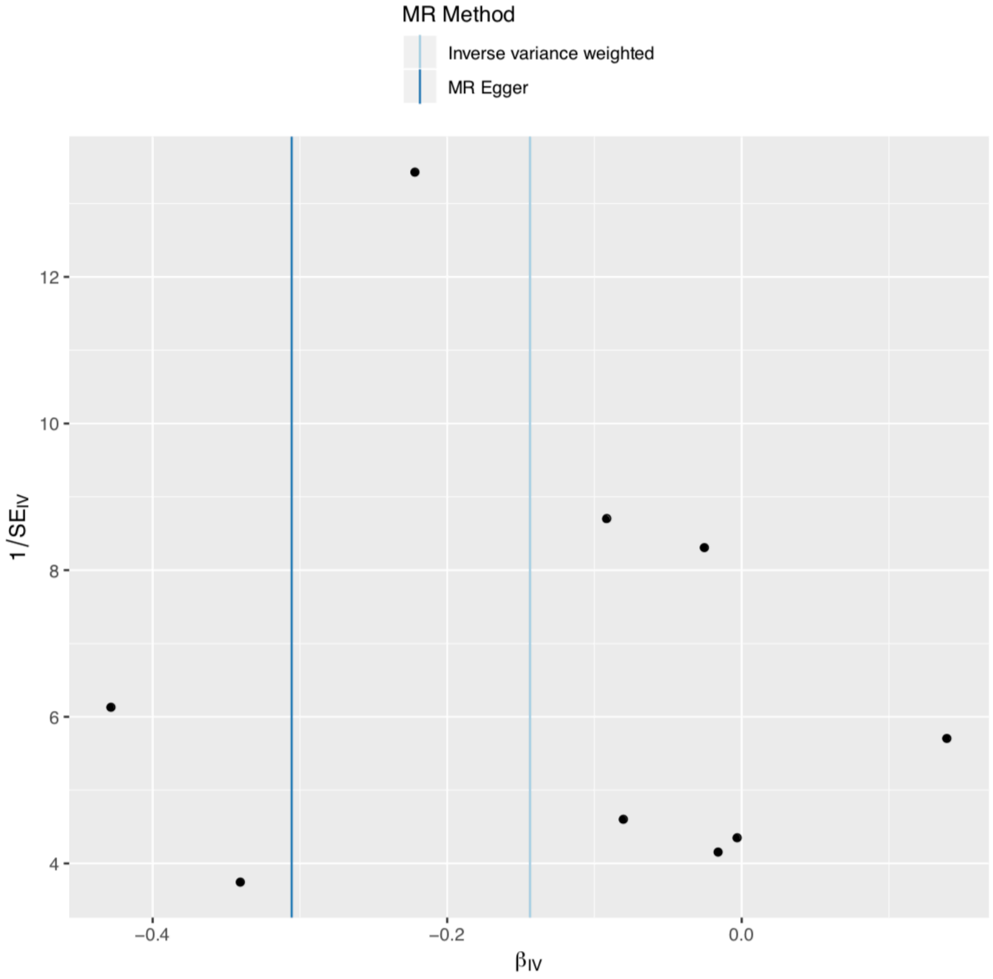

Fig. S4s.

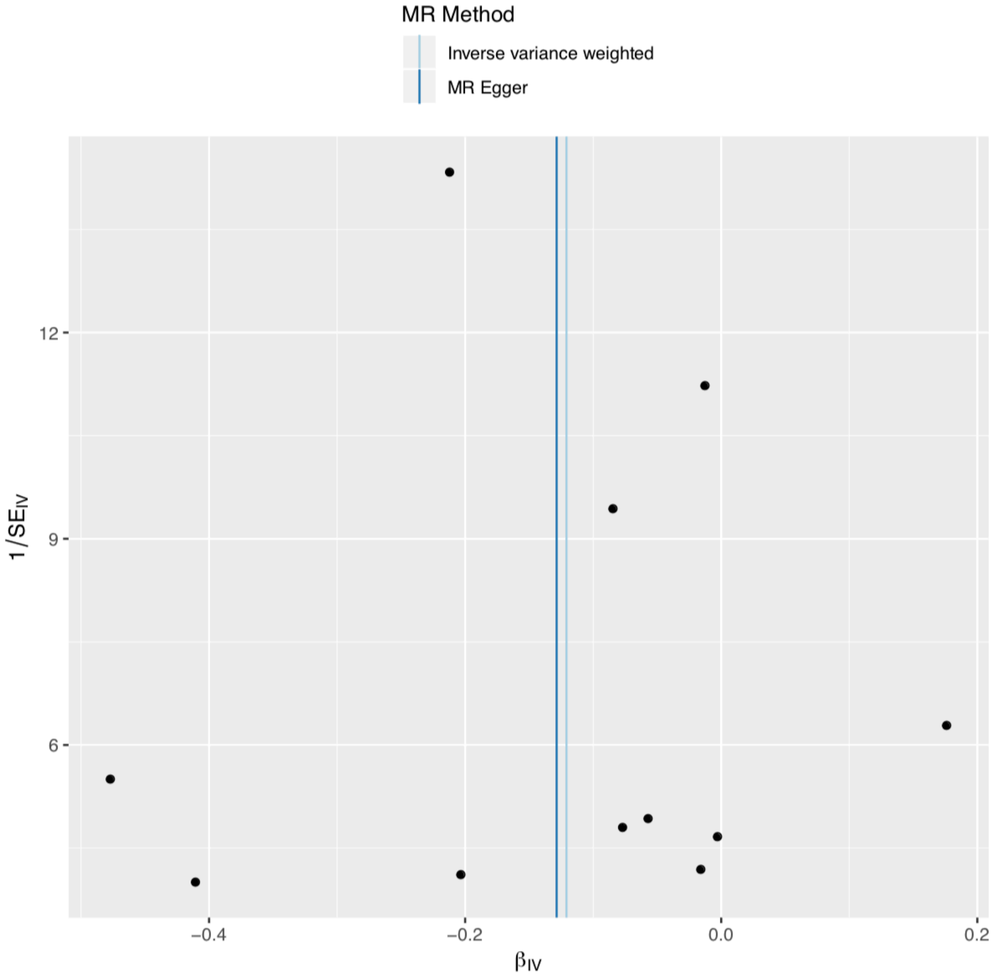

Fig. S4a-S4s. Funnel plots for each metabolite-AD pair when metabolite is the exposure and AD the outcome following univariable MR analyses.

Fig. S5a.

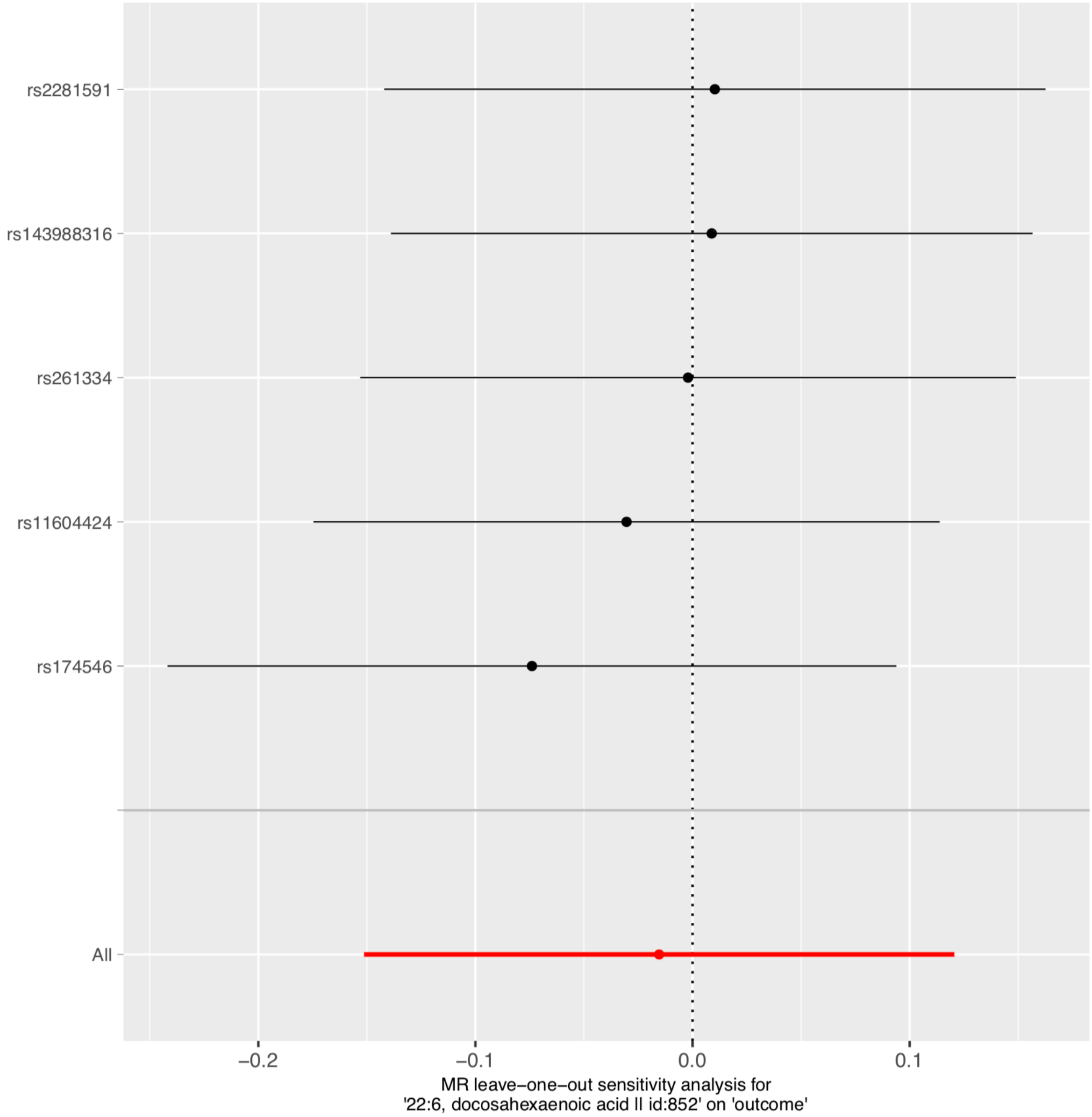
rs2281591
rs143988316
rs261334
rs11604424
rs174546
All
−0.2
−0.1
0.0
0.1
MR leave−one−out sensitivity analysis for
'22:6, docosahexaenoic acid II id:852' on 'outcome'

Fig. S5b.

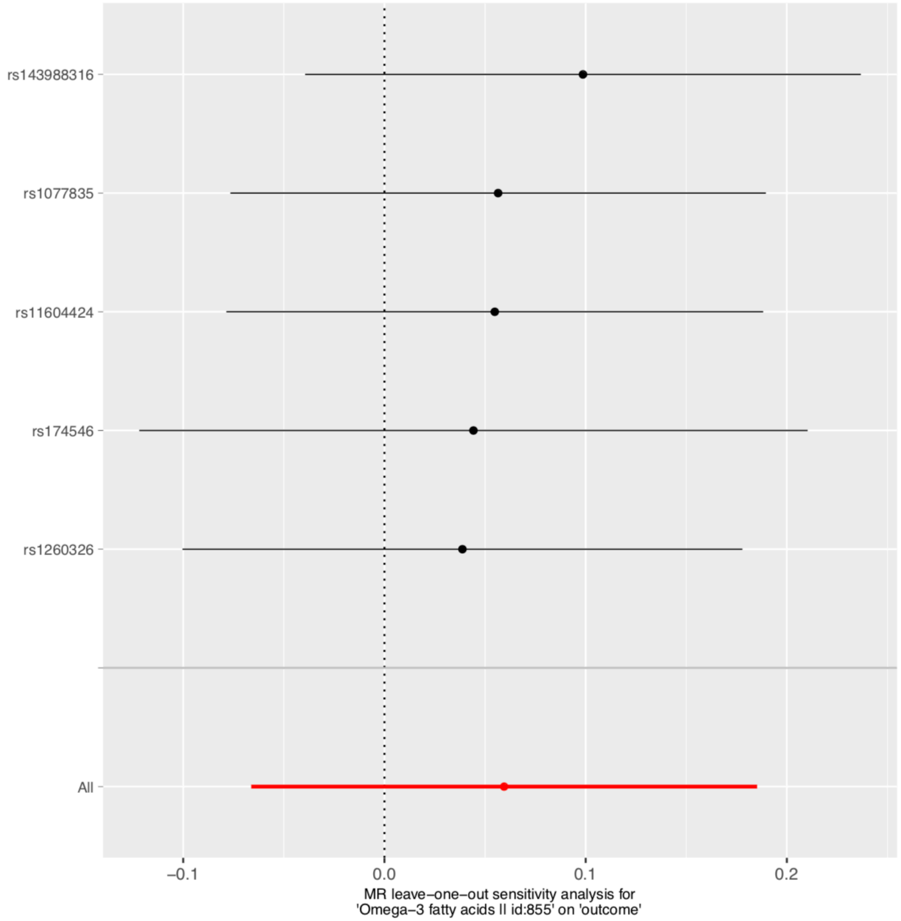

Fig. S5c.

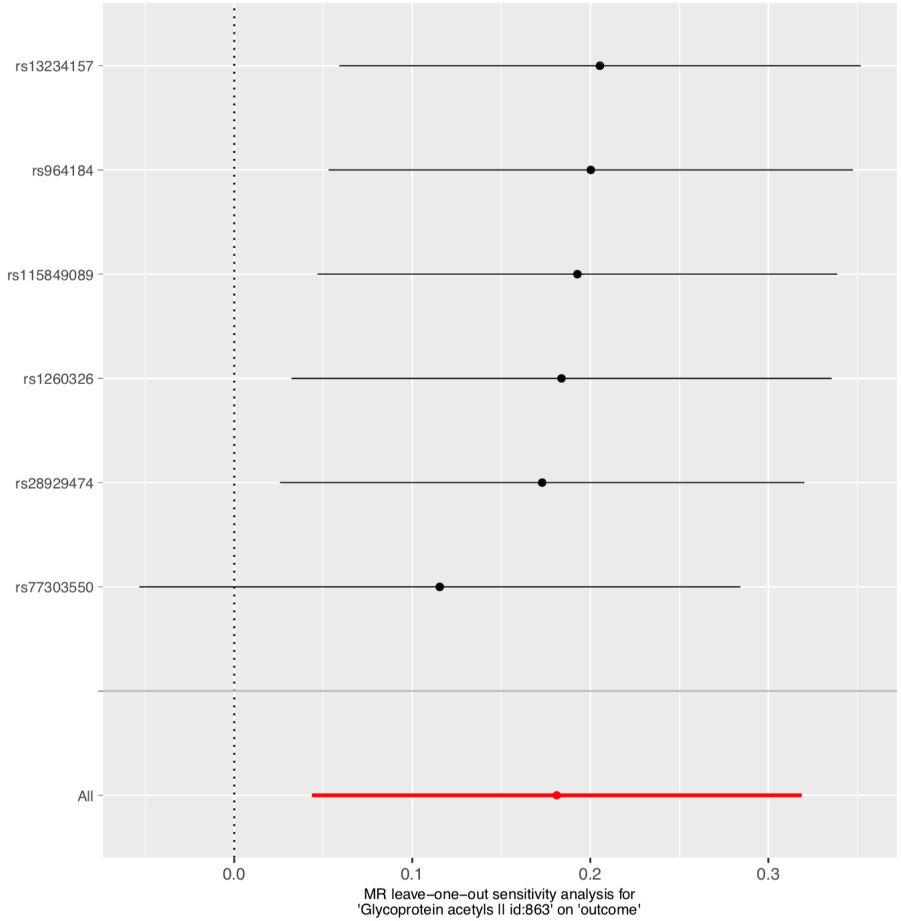

Fig. S5d.

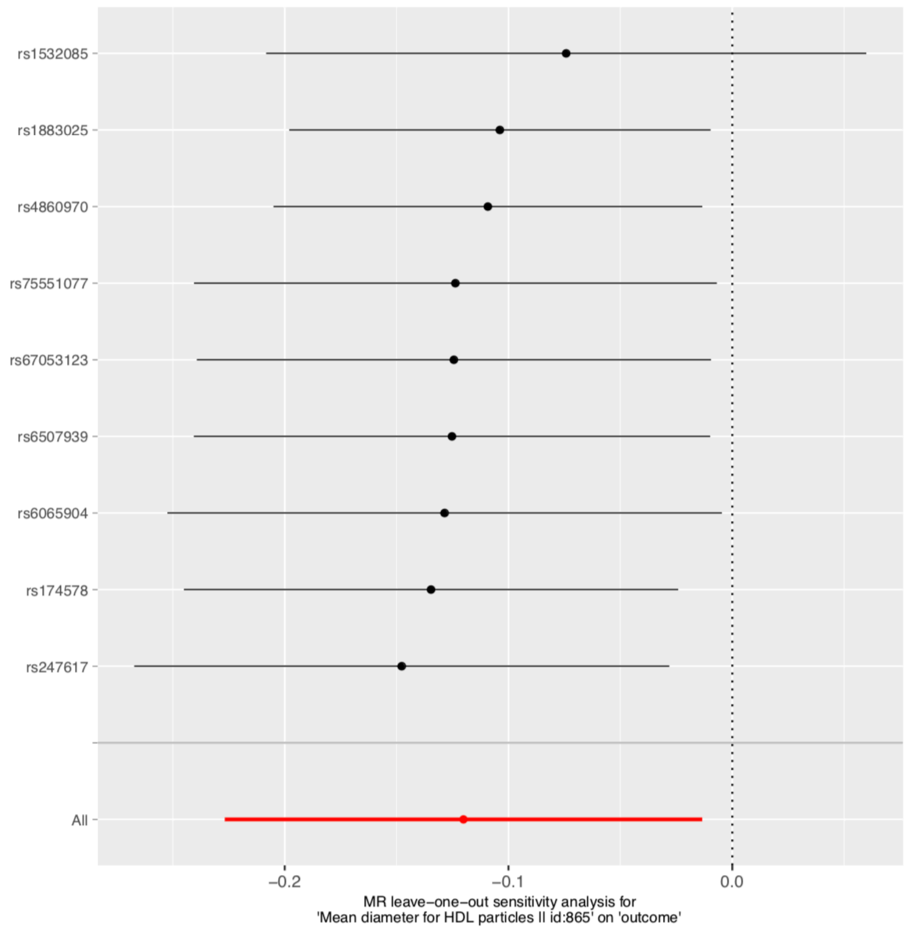

Fig. S5e.

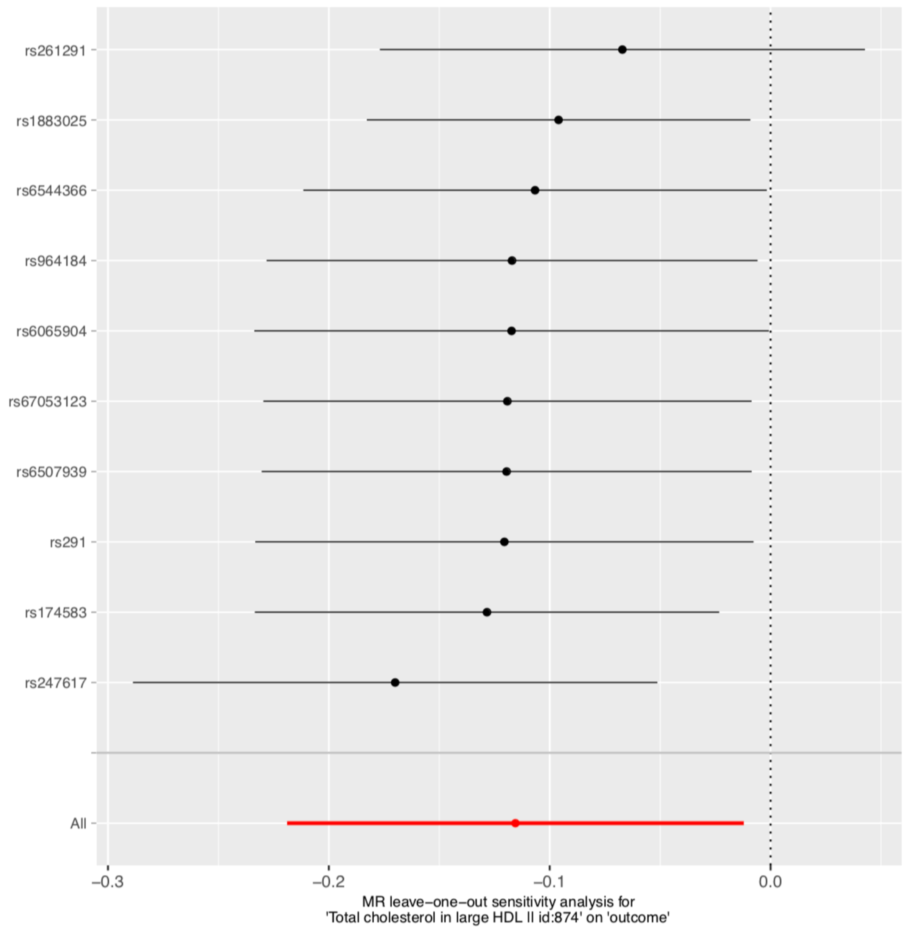

Fig. S5f.

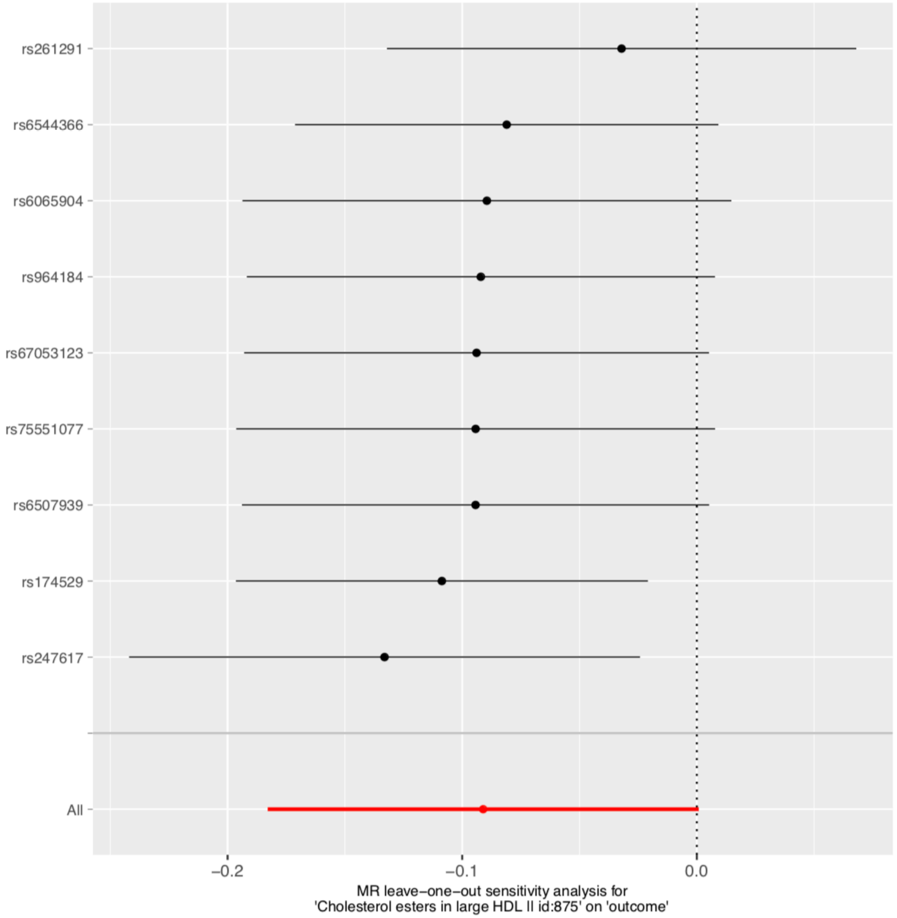

Fig. S5g.

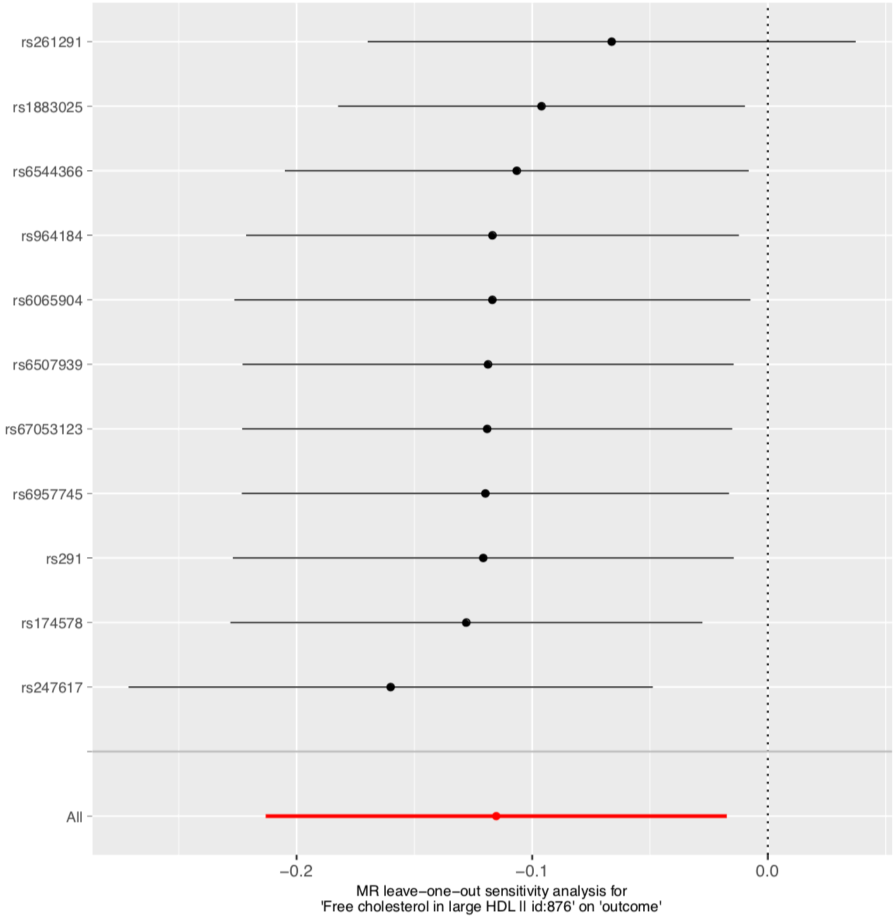

Fig. S5h.

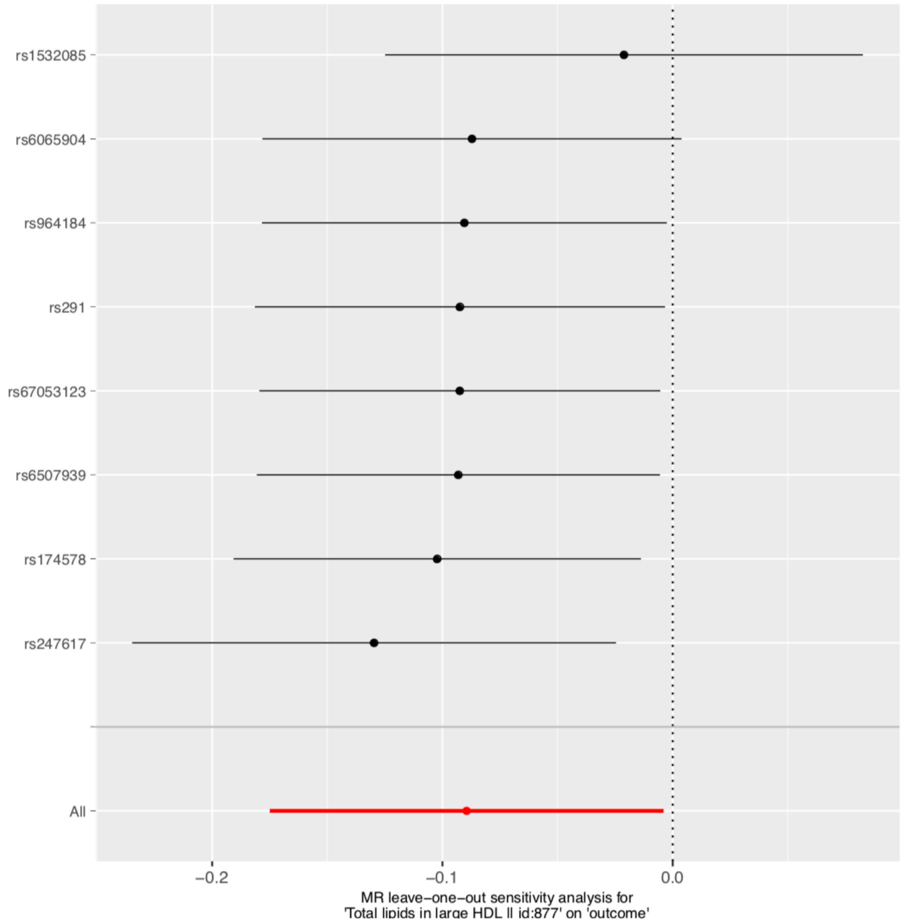

Fig. S5i.

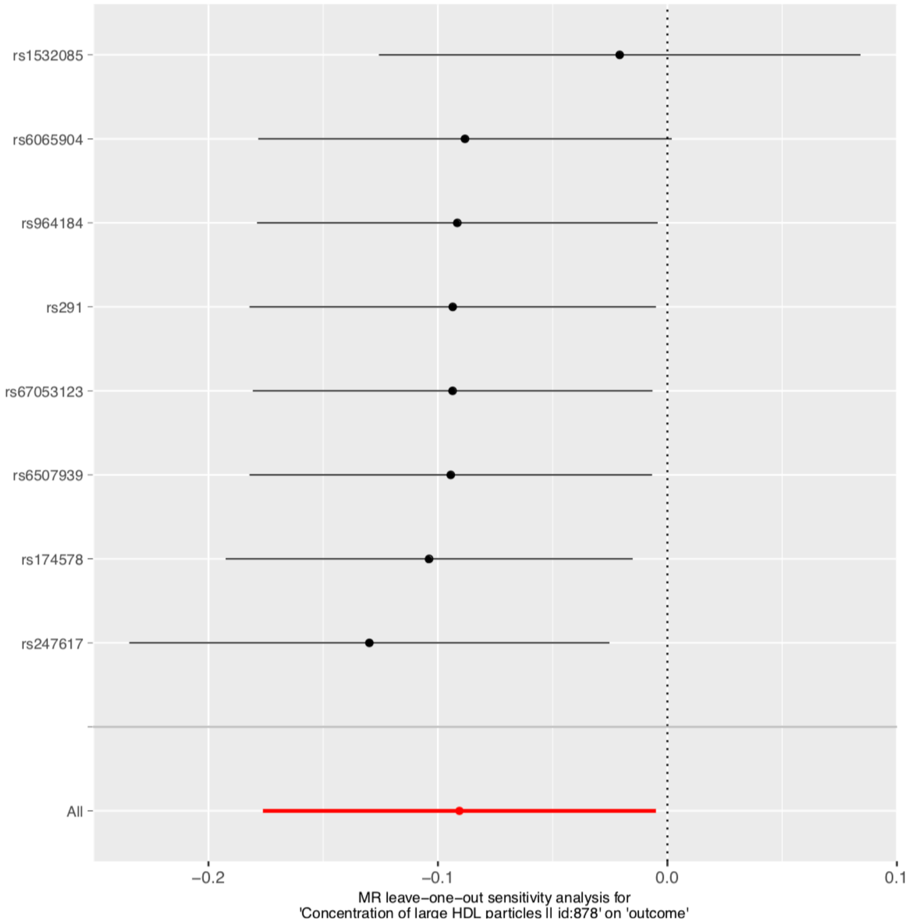

Fig. S5j.

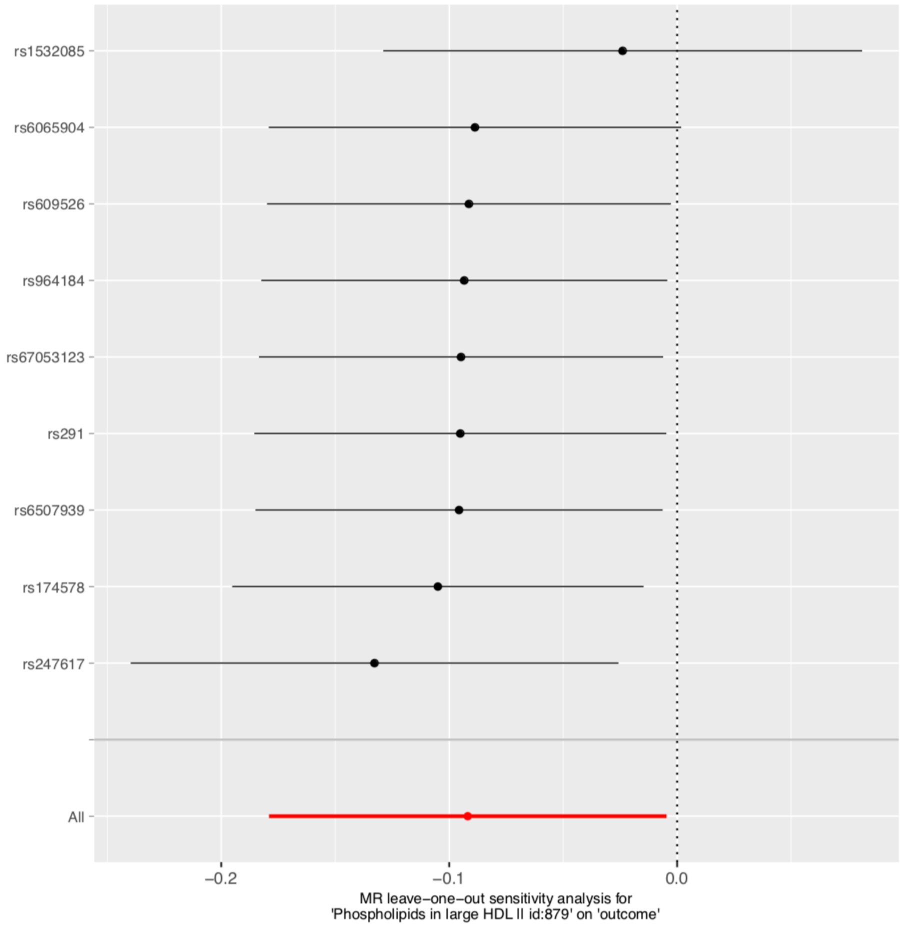

Fig. S5k.

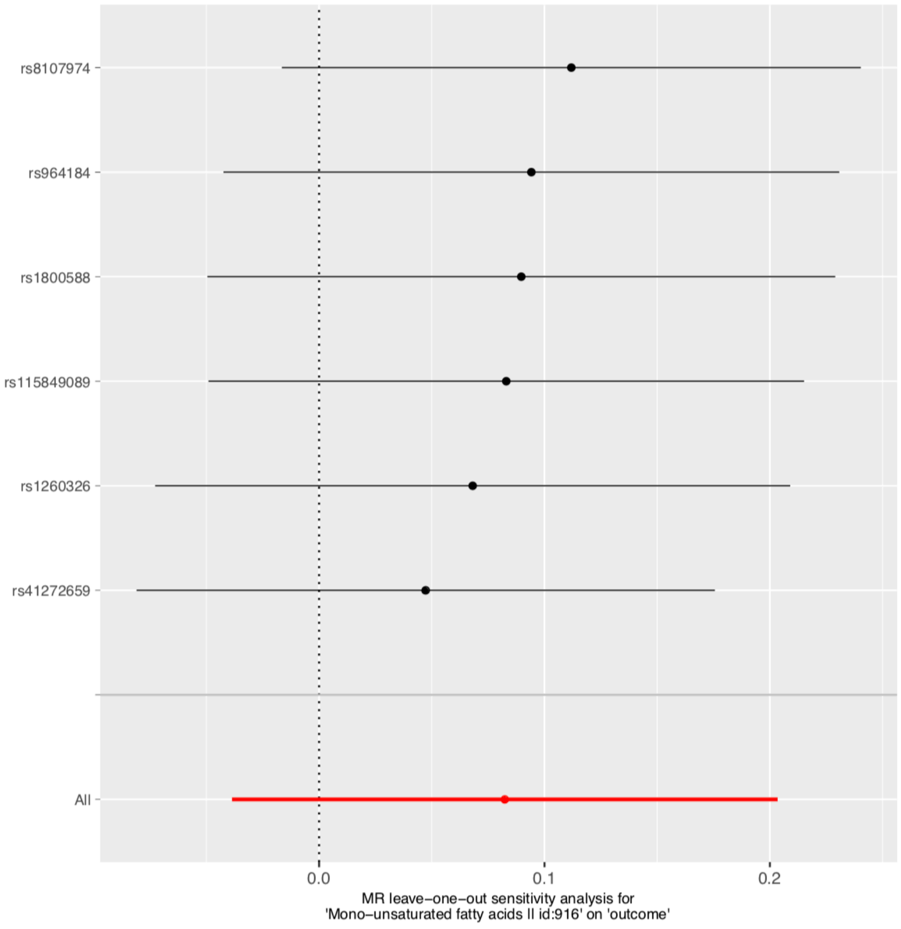

Fig. S5l.

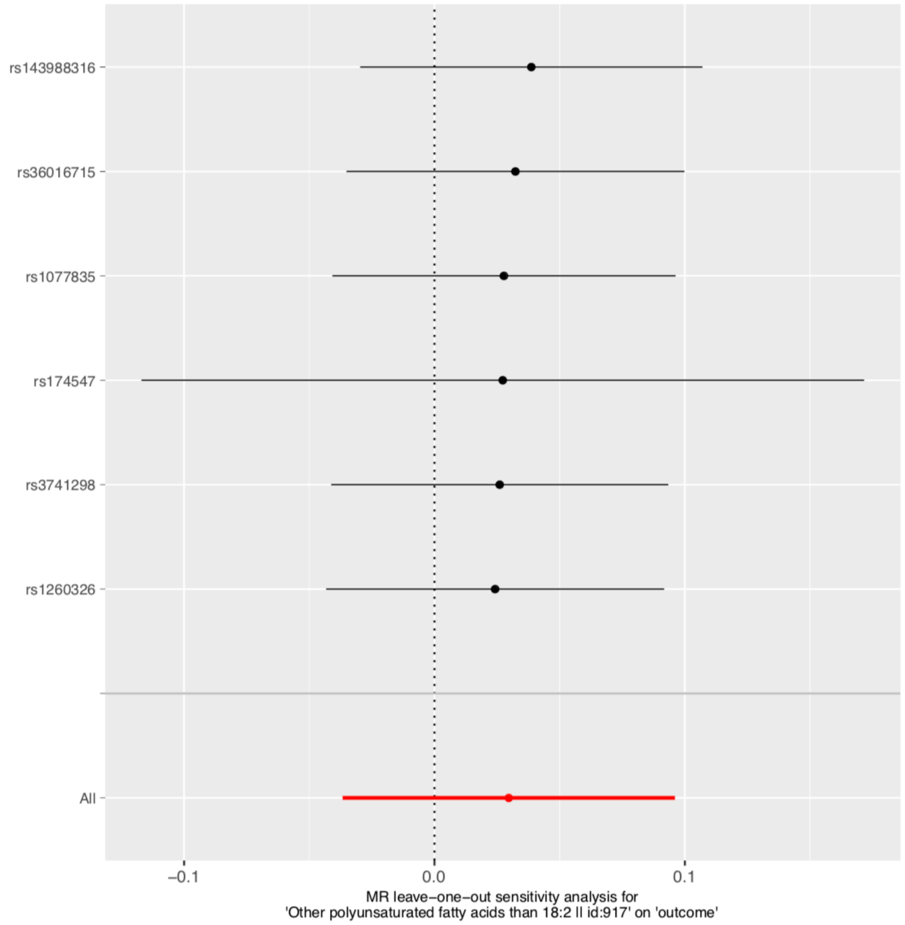

Fig. S5m.

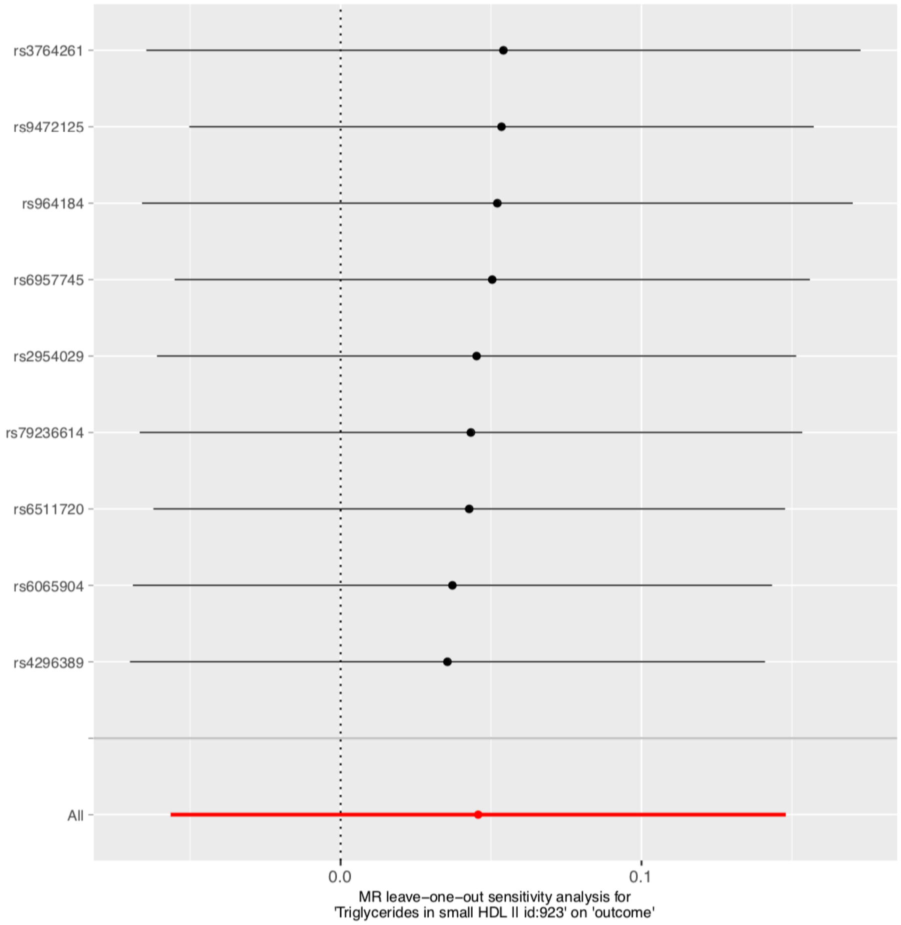

Fig. S5n.

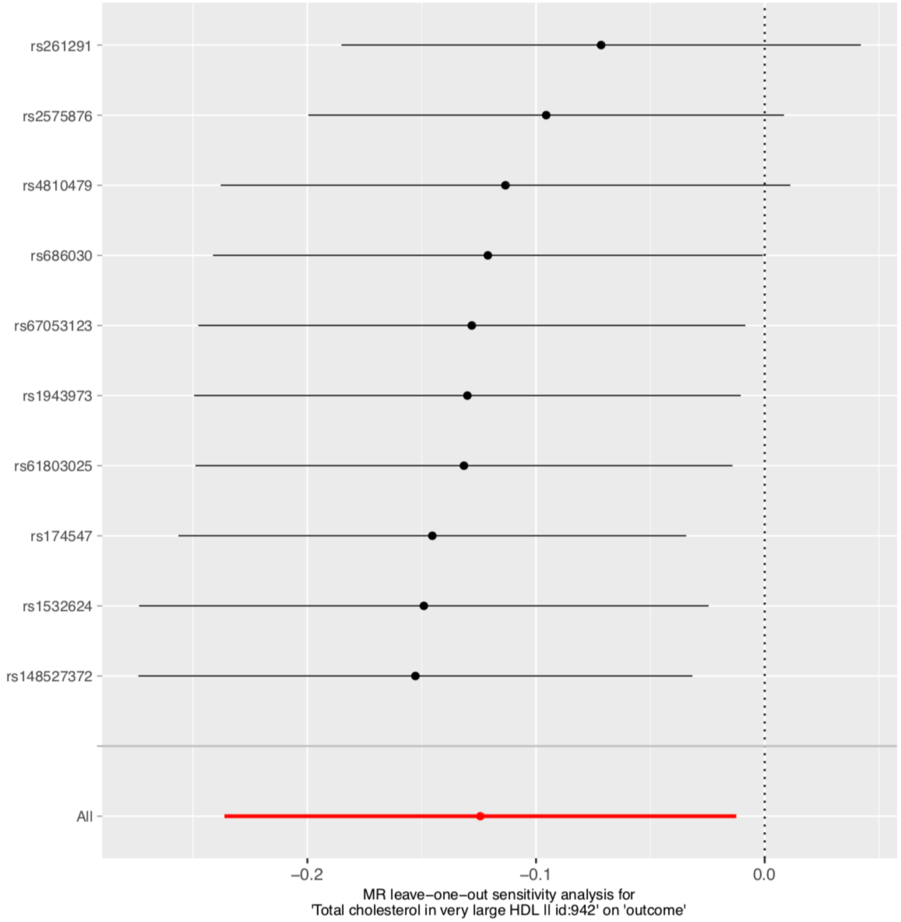

Fig. S5o.

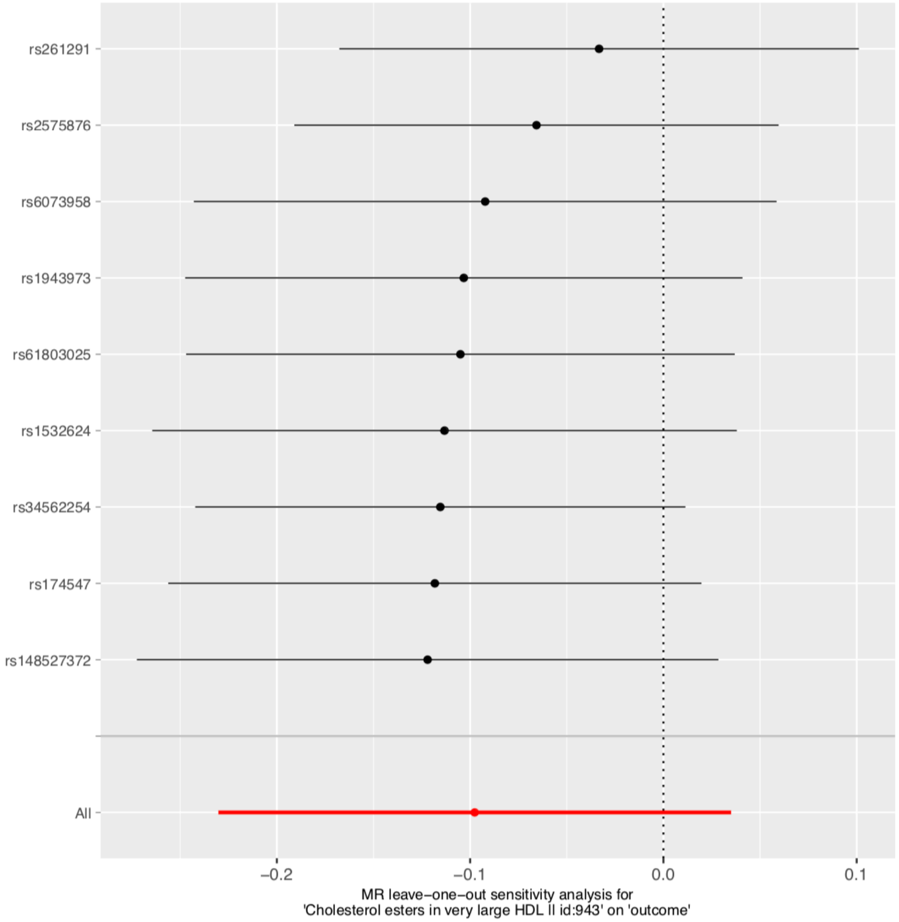

Fig. S5p.

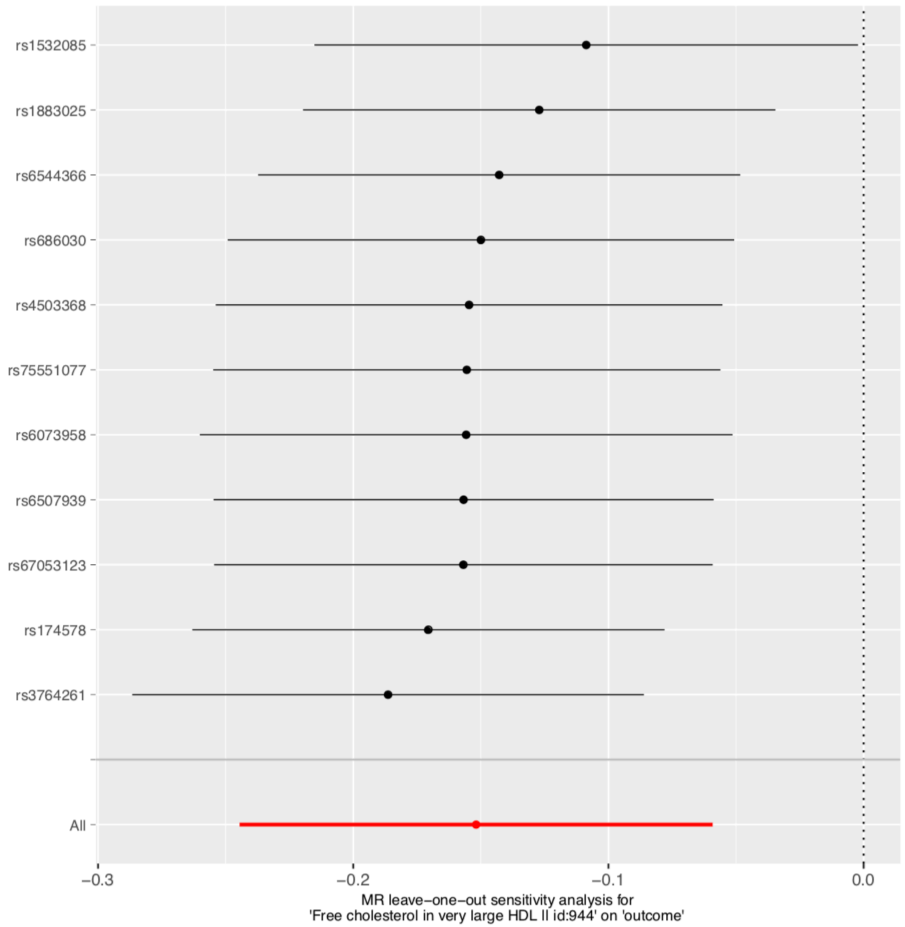

Fig. S5q.

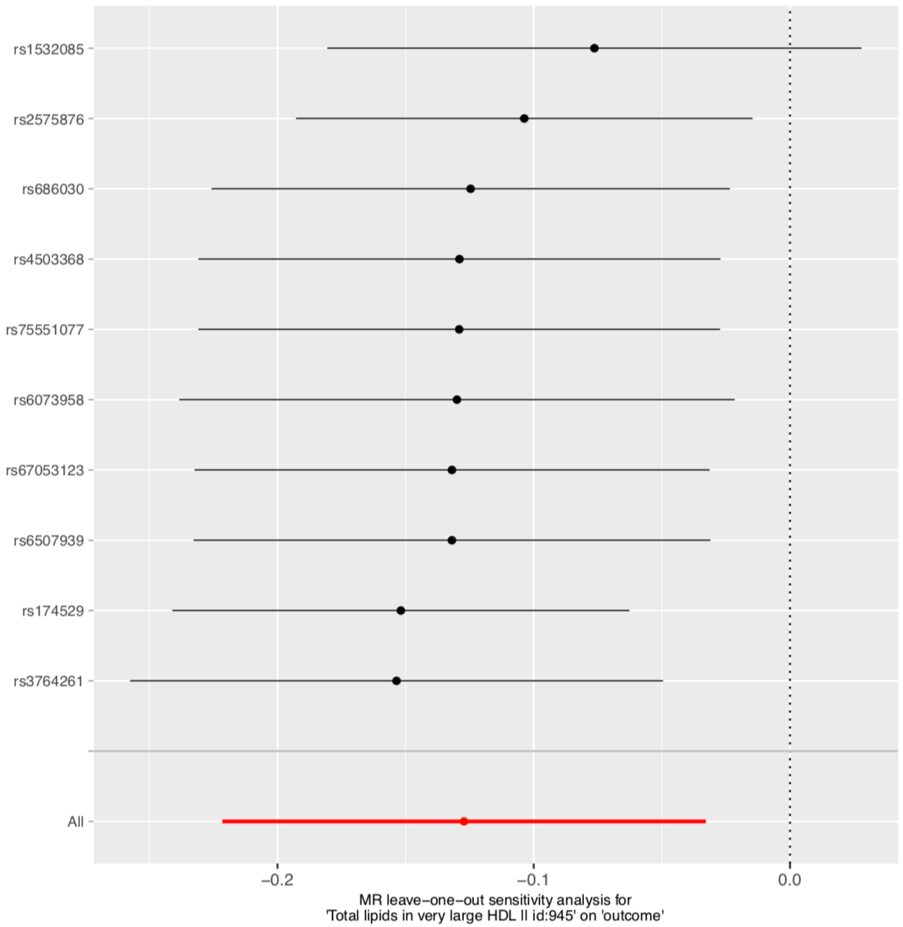

Fig. S5r.

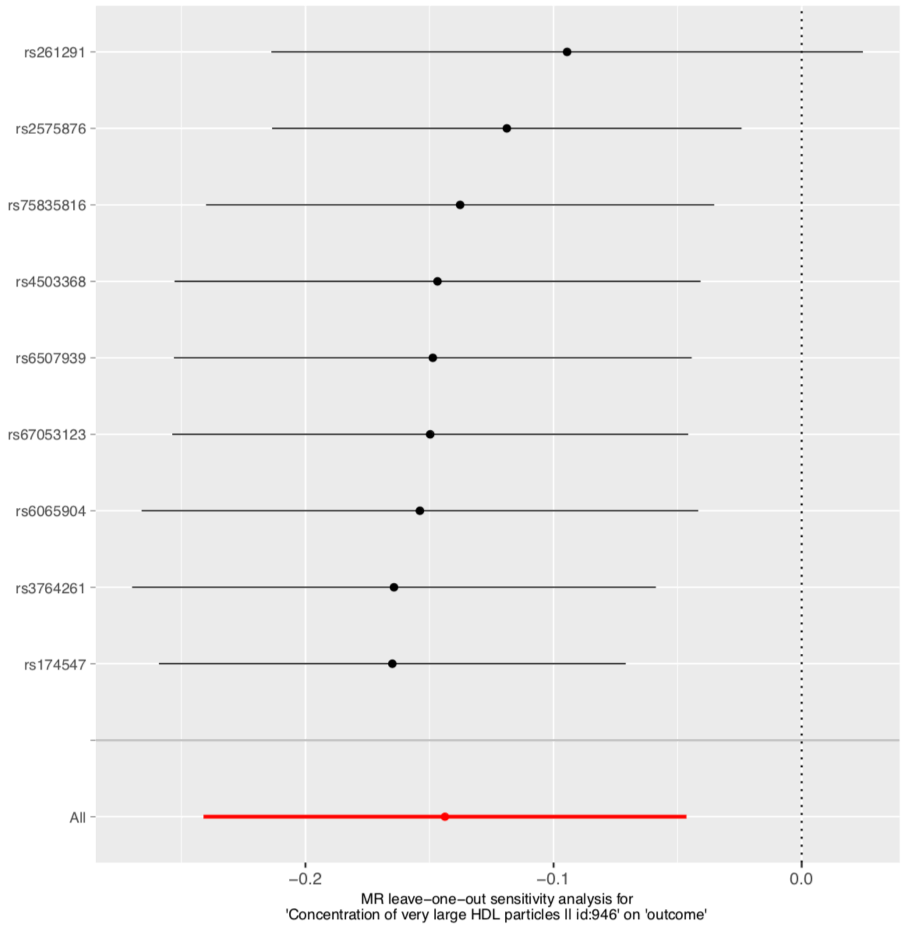

Fig. S5s.

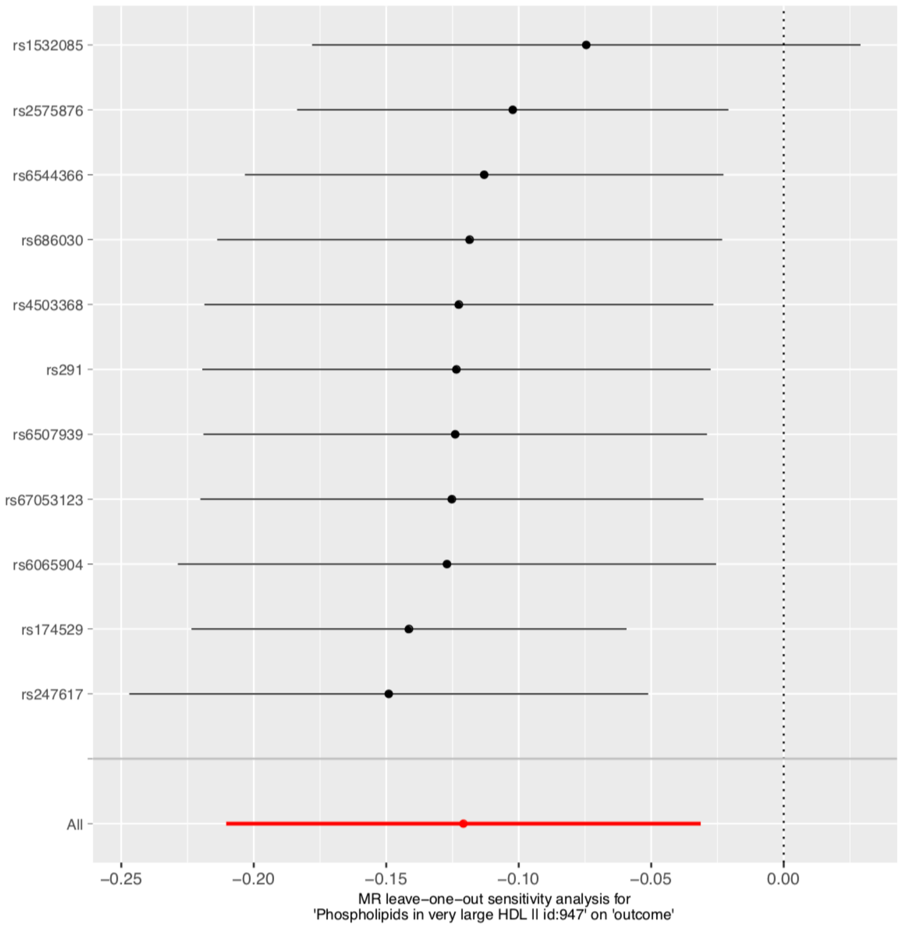

Fig. S5a-S5s. Leave-one-out (LOO) plots for each metabolite-AD pair when metabolite is the exposure and AD the outcome following univariable MR analyse

Fig. S6a.

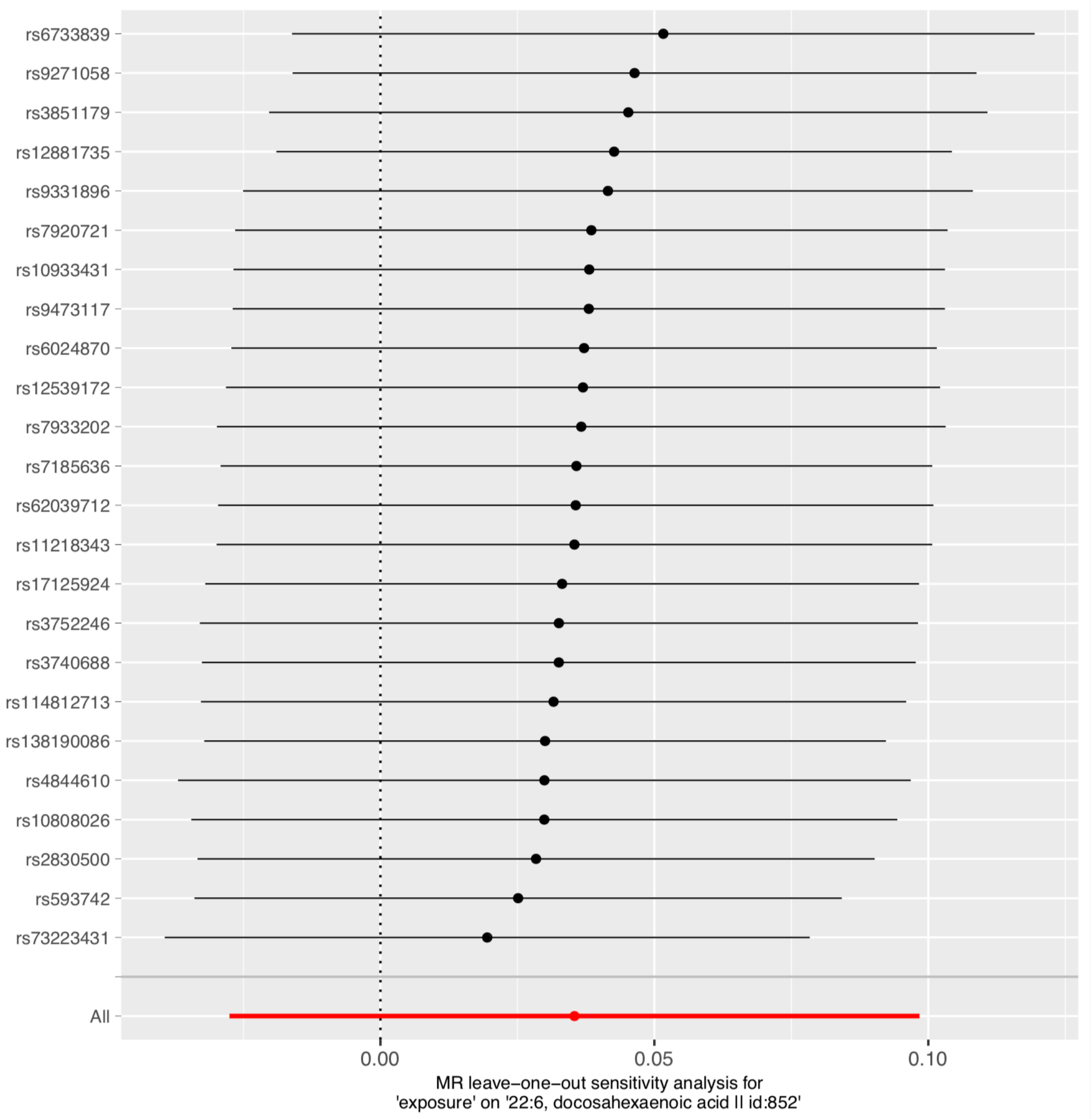
rs6733839
rs9271058
rs3851179
rs12881735
rs9331896
rs7920721
rs10933431
rs9473117
rs6024870
rs12539172
rs7933202
rs7185636
rs62039712
rs11218343
rs17125924
rs3752246
rs3740688
rs114812713
rs138190086
rs4844610
rs10808026
rs2830500
rs593742
rs73223431
All
0.00
0.05
0.10
MR leave−one−out sensitivity analysis for
'exposure' on '22:6, docosahexaenoic acid II id:852'

Fig. S6b.

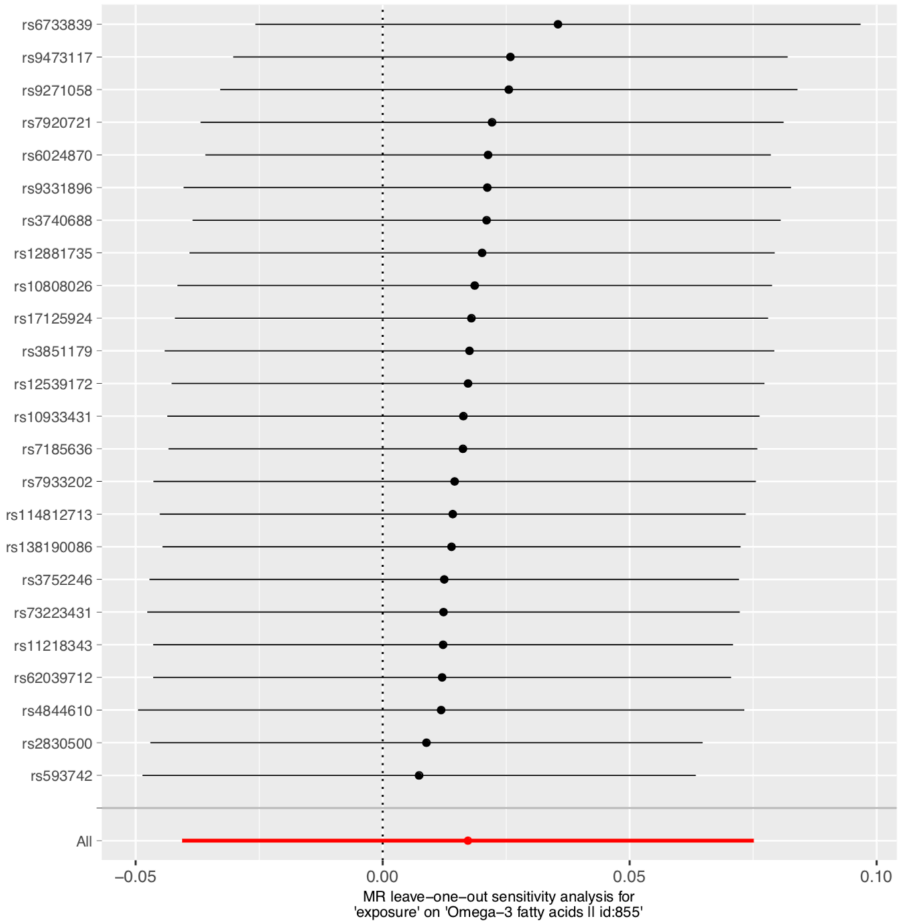

Fig. S6c.

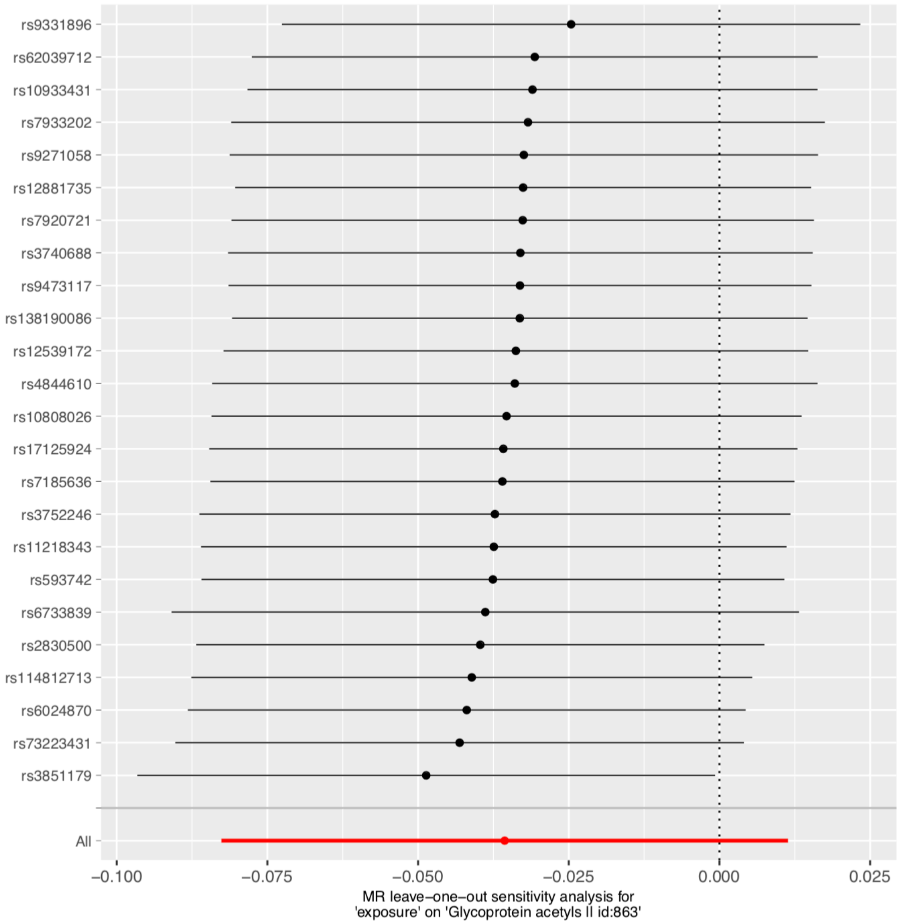

Fig. S6d.

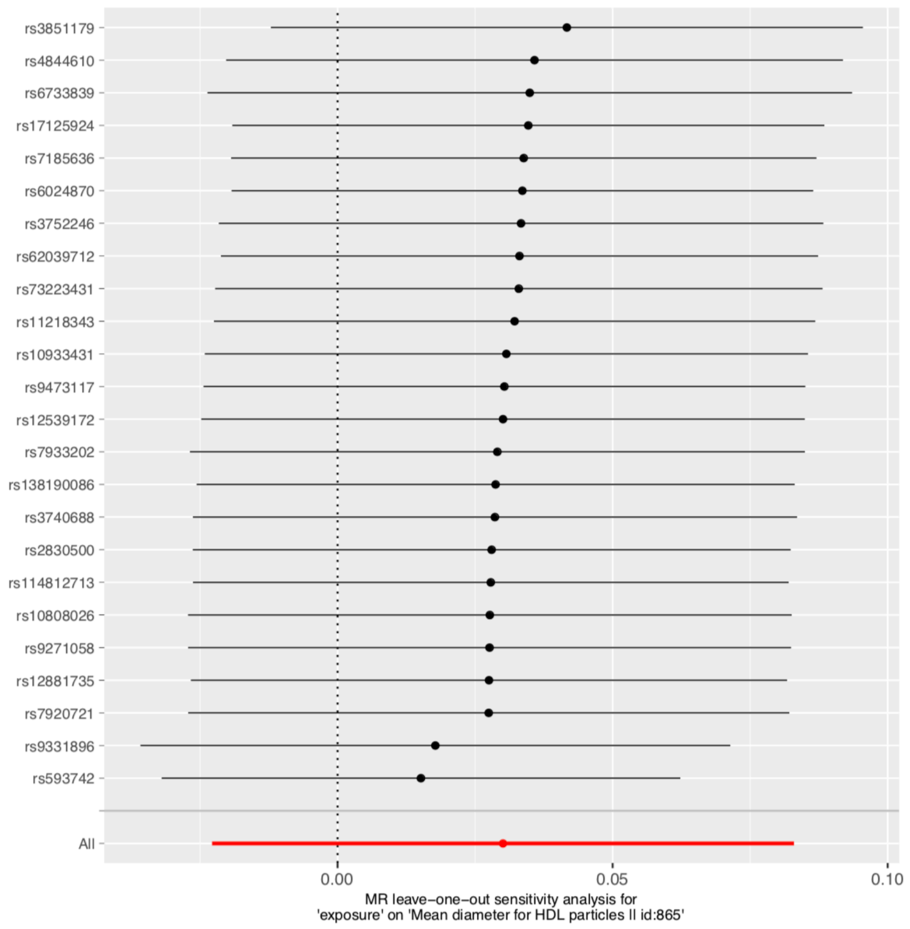

Fig. S6e.

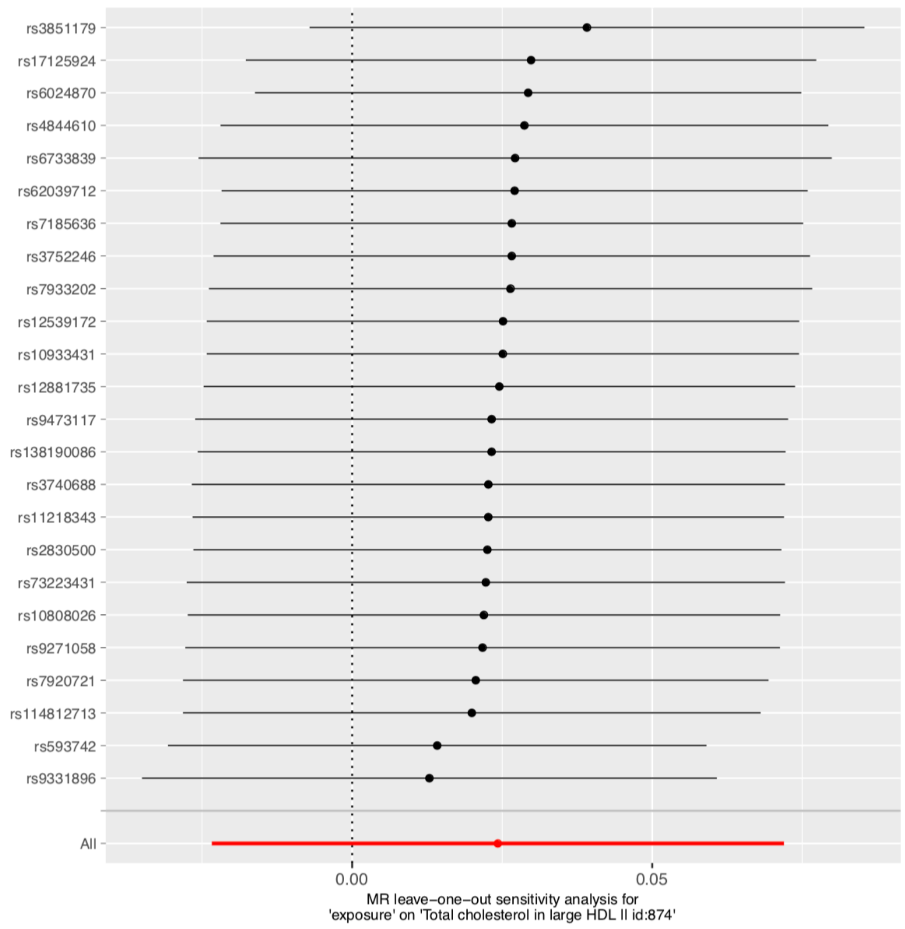

Fig. S6f.

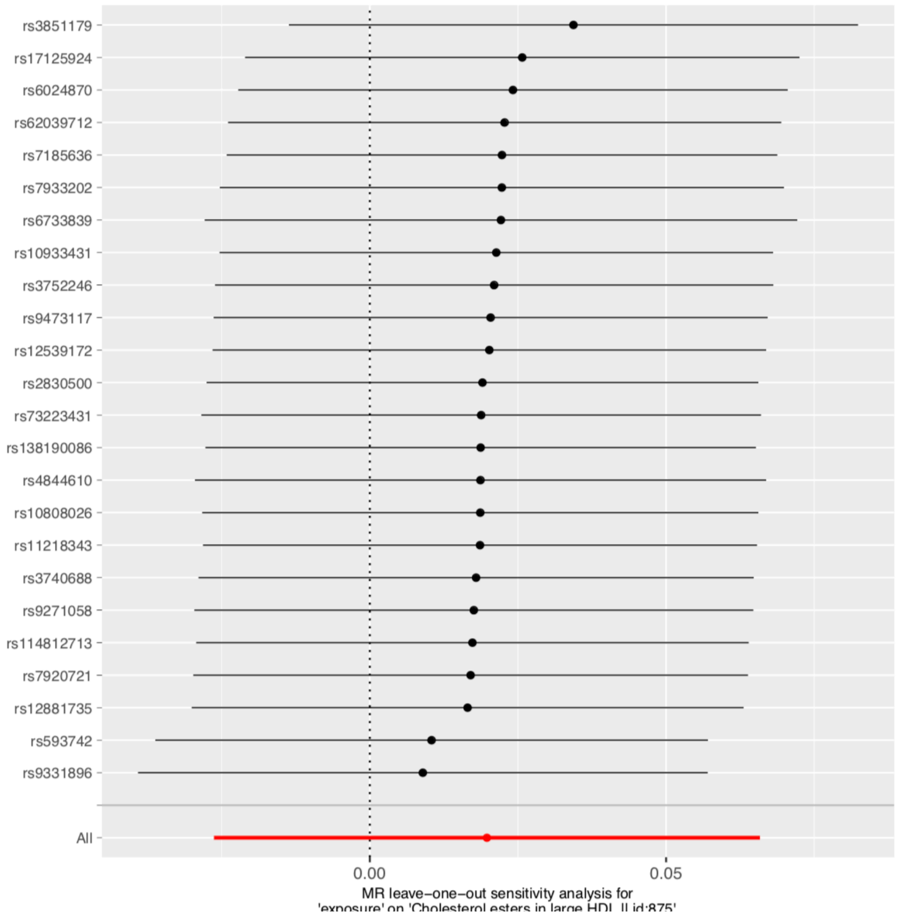

Fig. S6g.

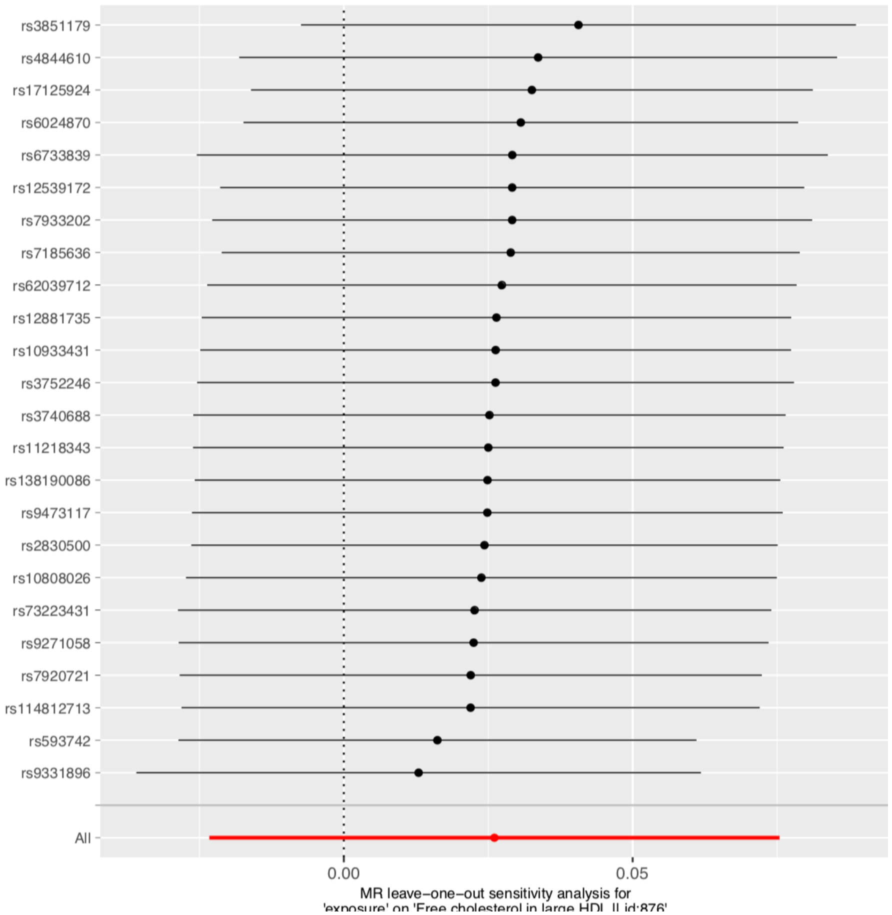

Fig. S6h.

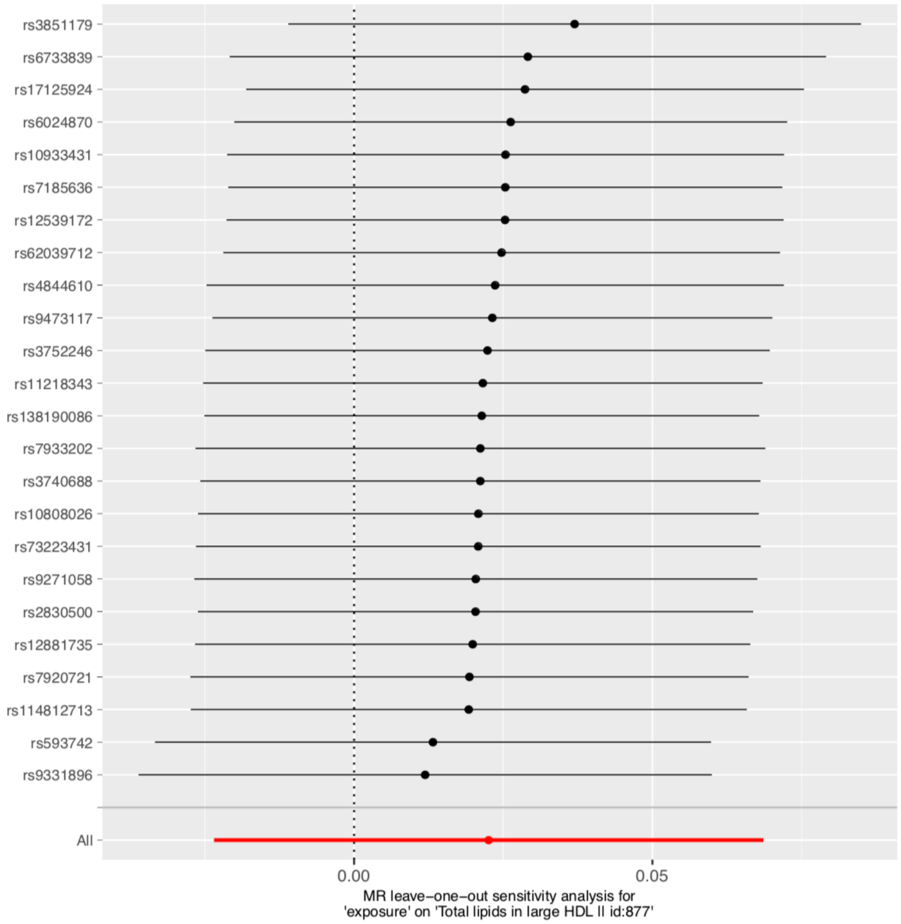

Fig. S6i.

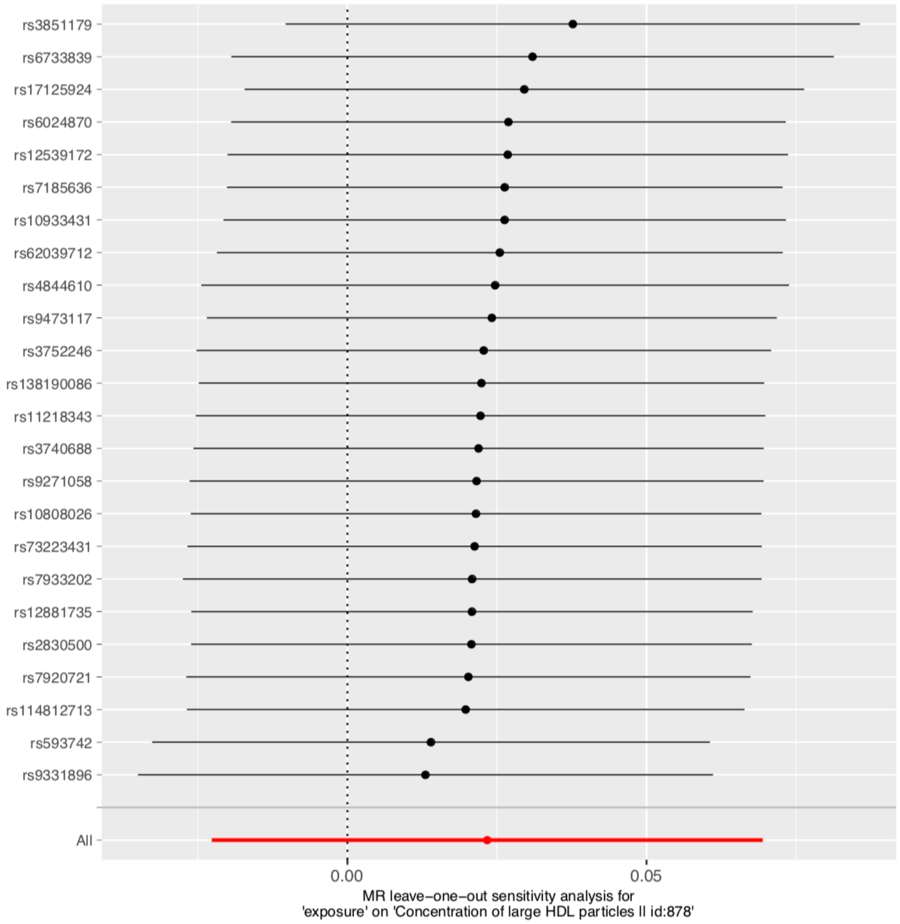

Fig. S6j.

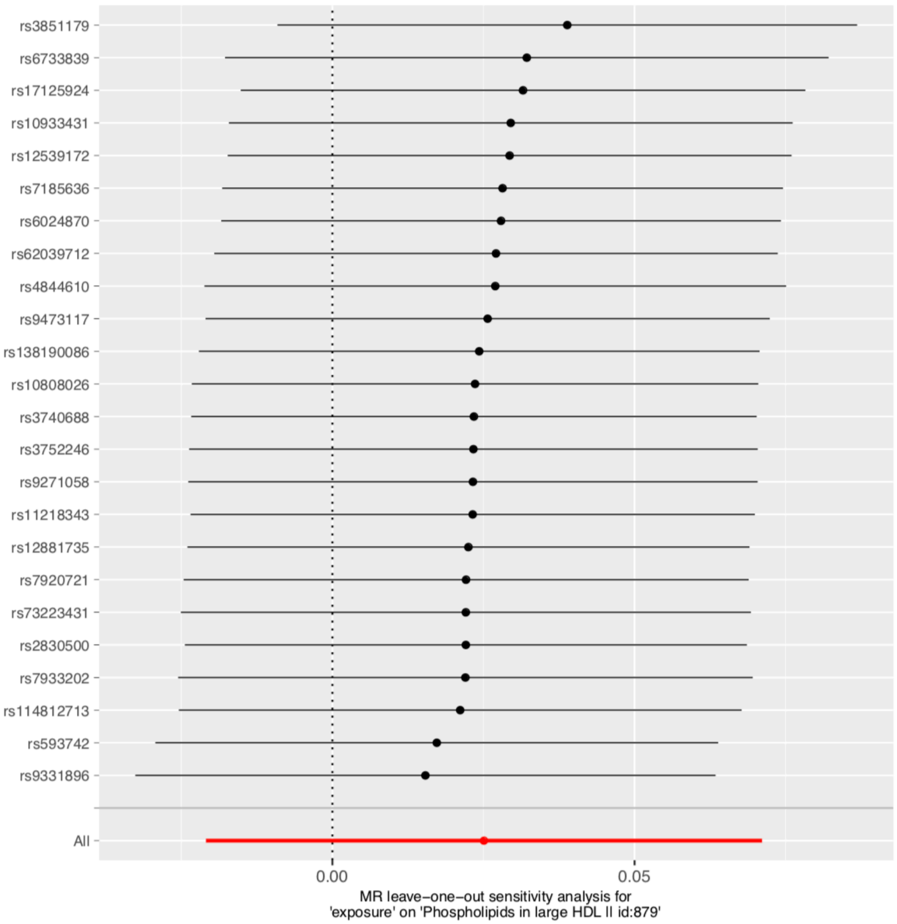

Fig. S6k.

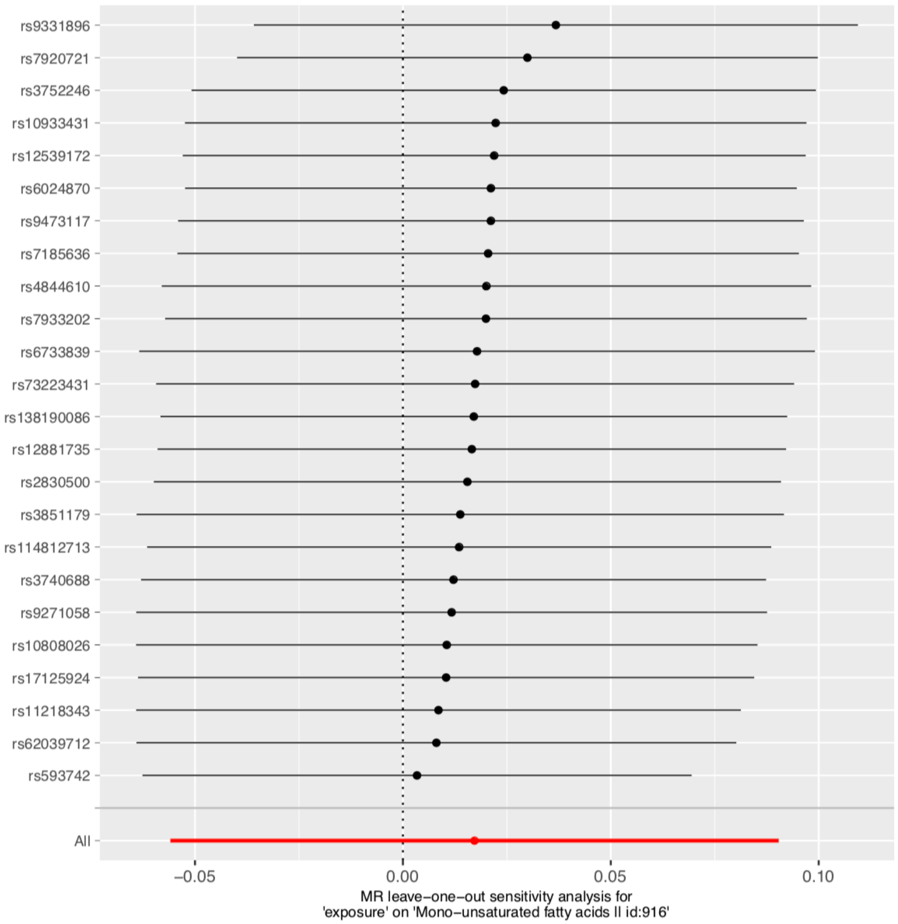

Fig. S6l.

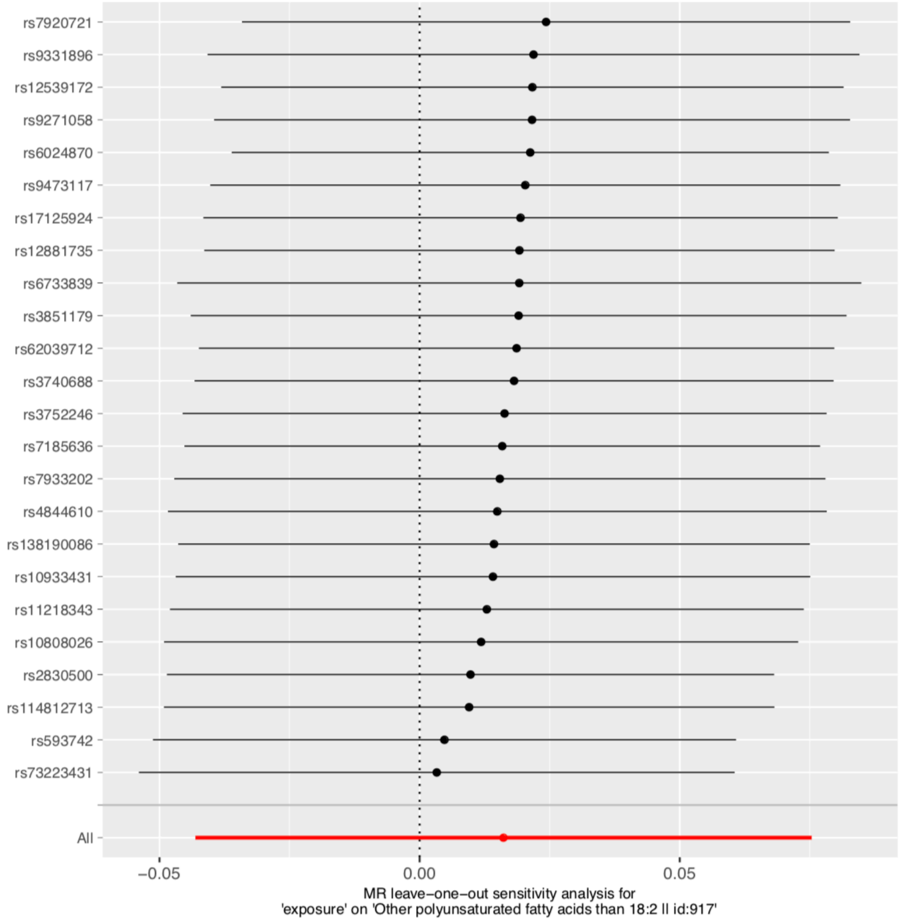

Fig. S6m.

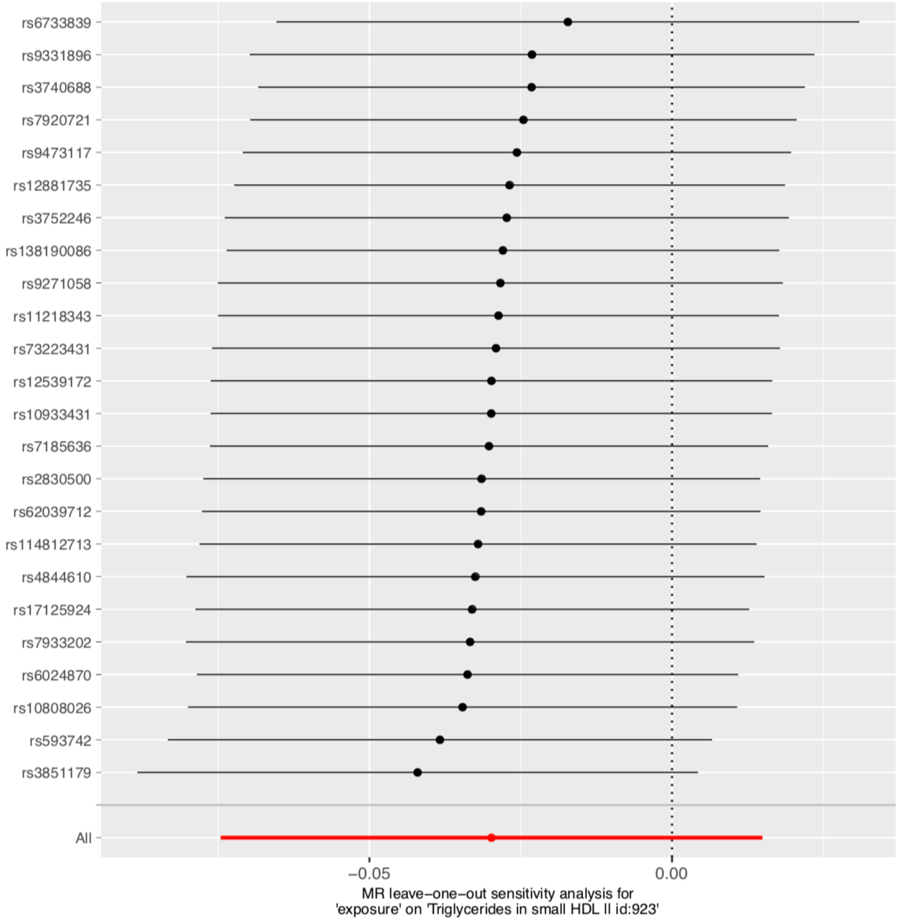

Fig. S6n.

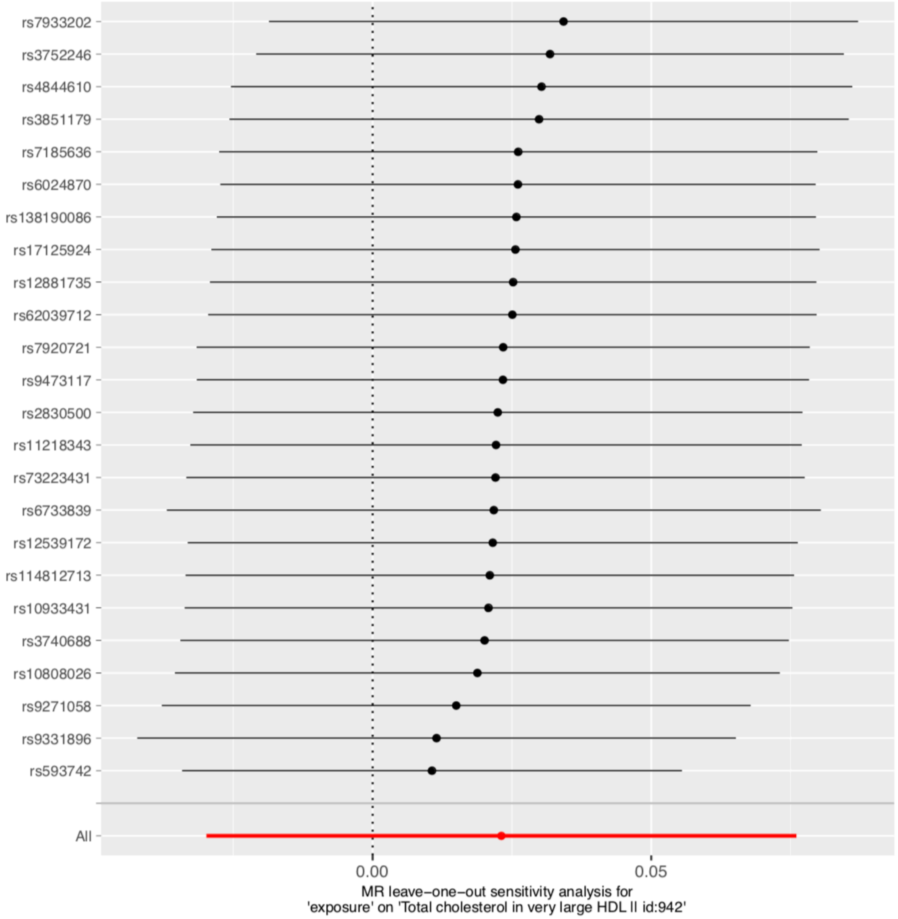

Fig. S6o.

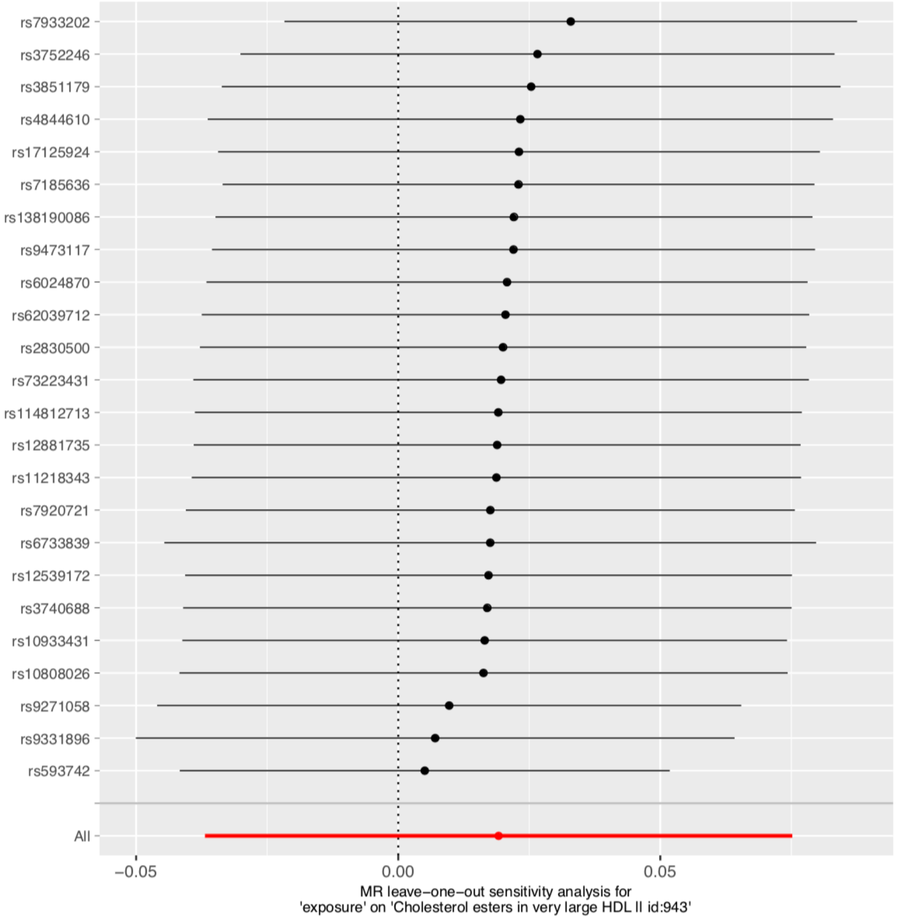

Fig. S6p.

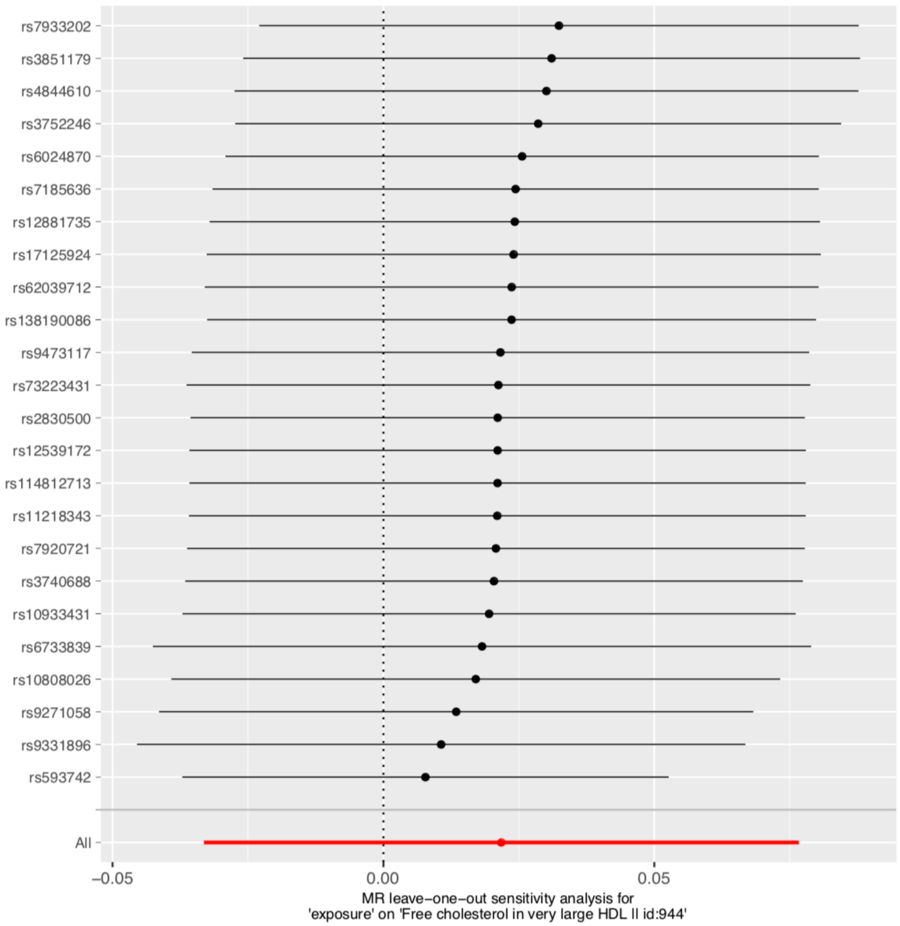

Fig. S6q.

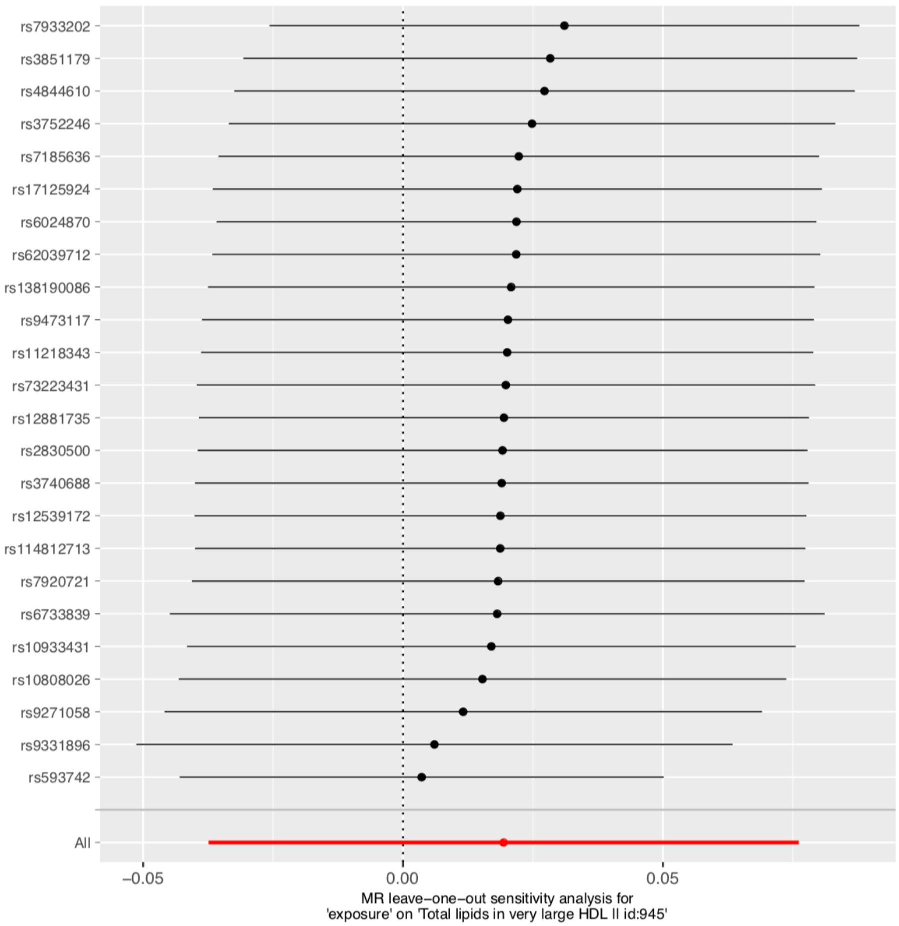

Fig. S6r.

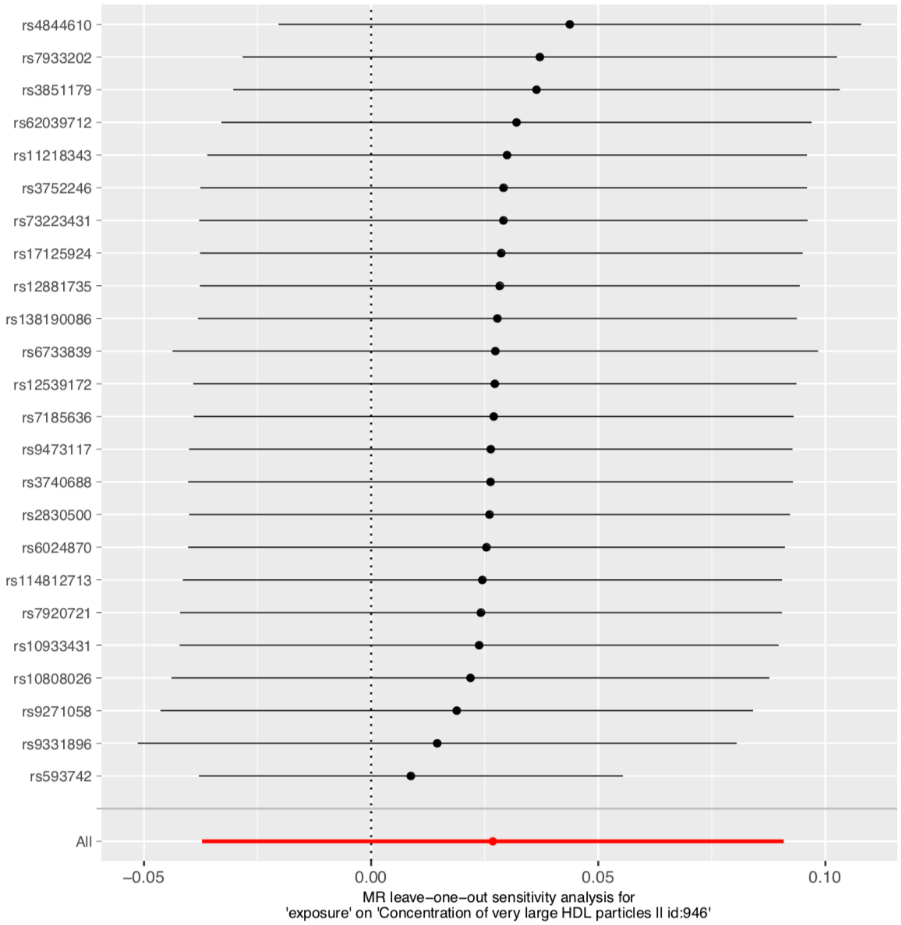

Fig. S6s.

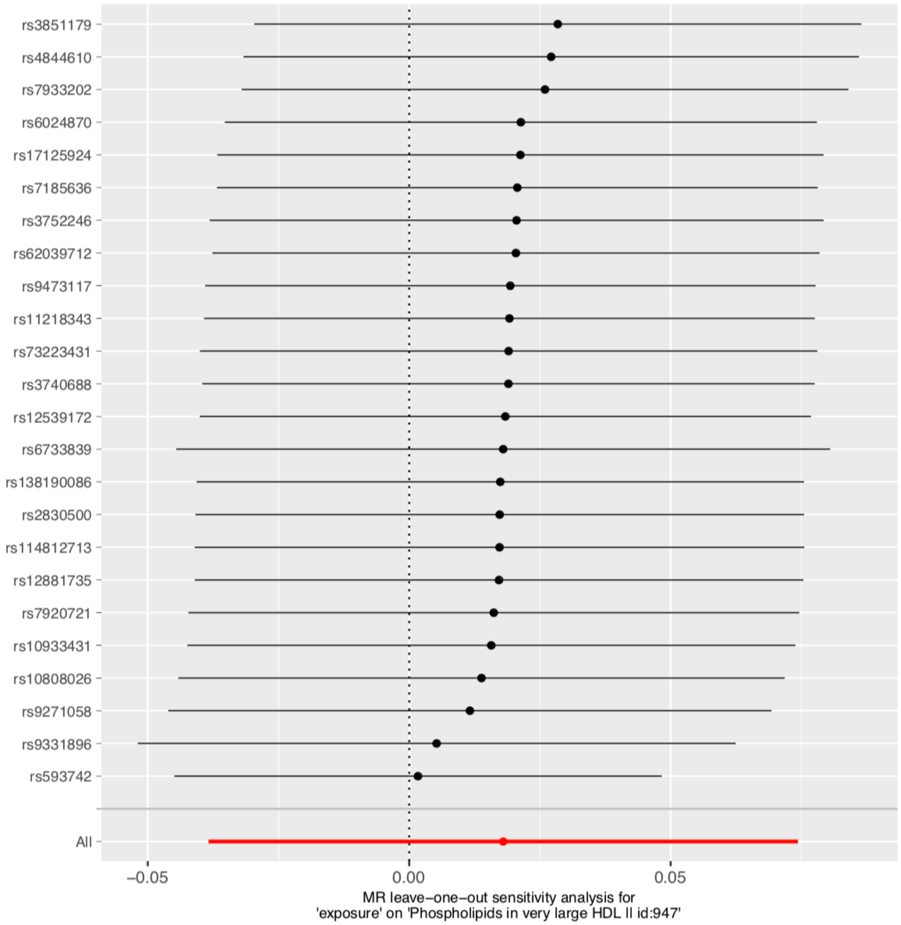

Fig. S6a-S6s. Leave-one-out (LOO) plots for each AD-metabolite pair when AD is the exposure and each metabolite the outcome following univariable MR analyses.

Fig. S7a.

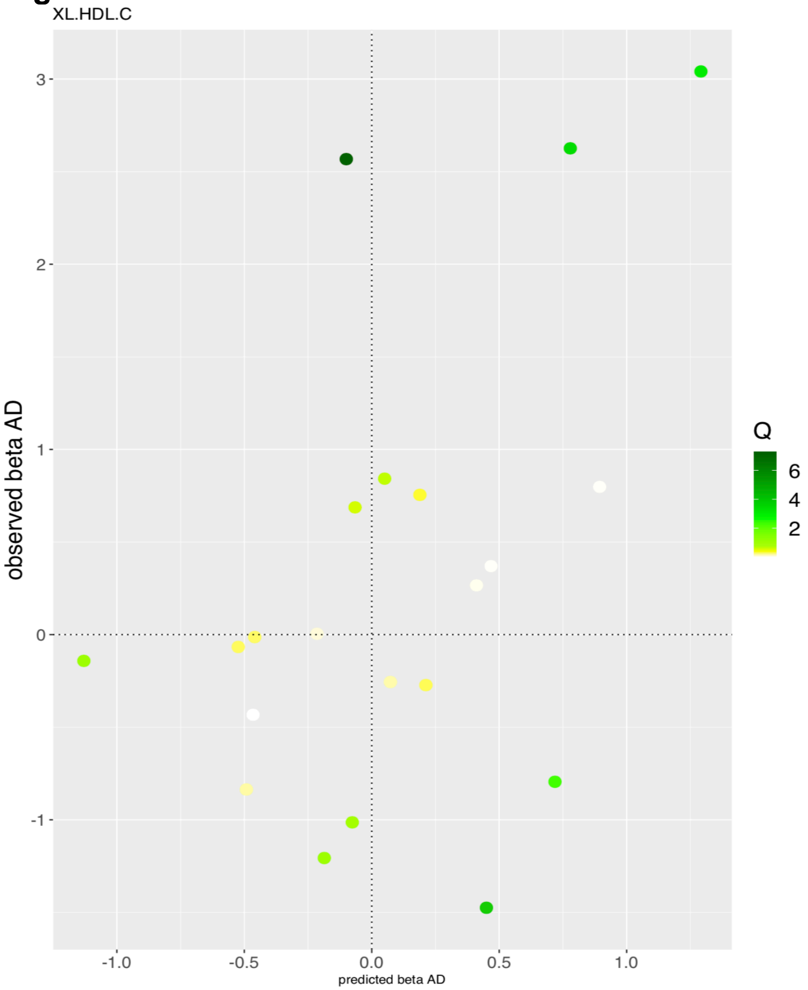

Fig. s7b.

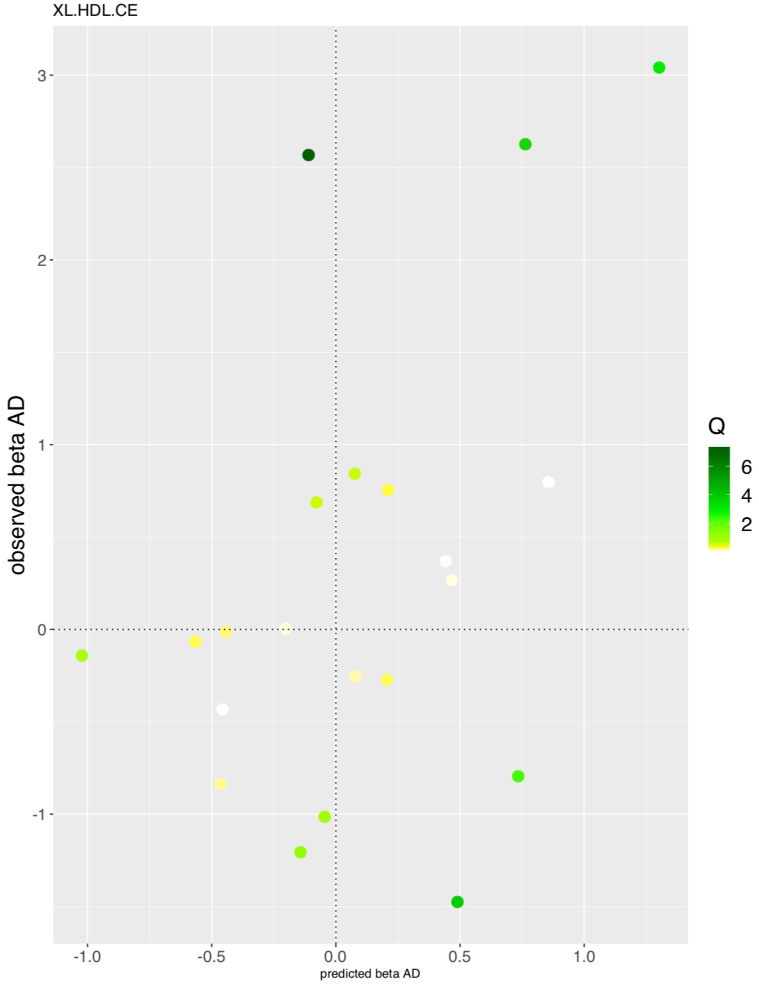

Fig. S7c.

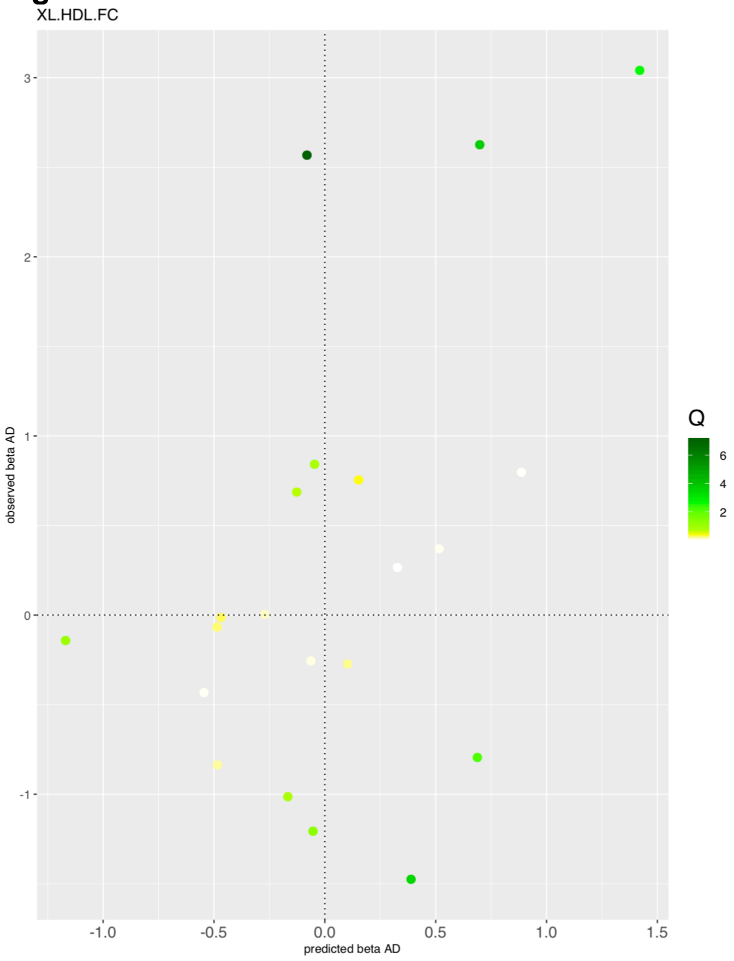

Fig. S7d.

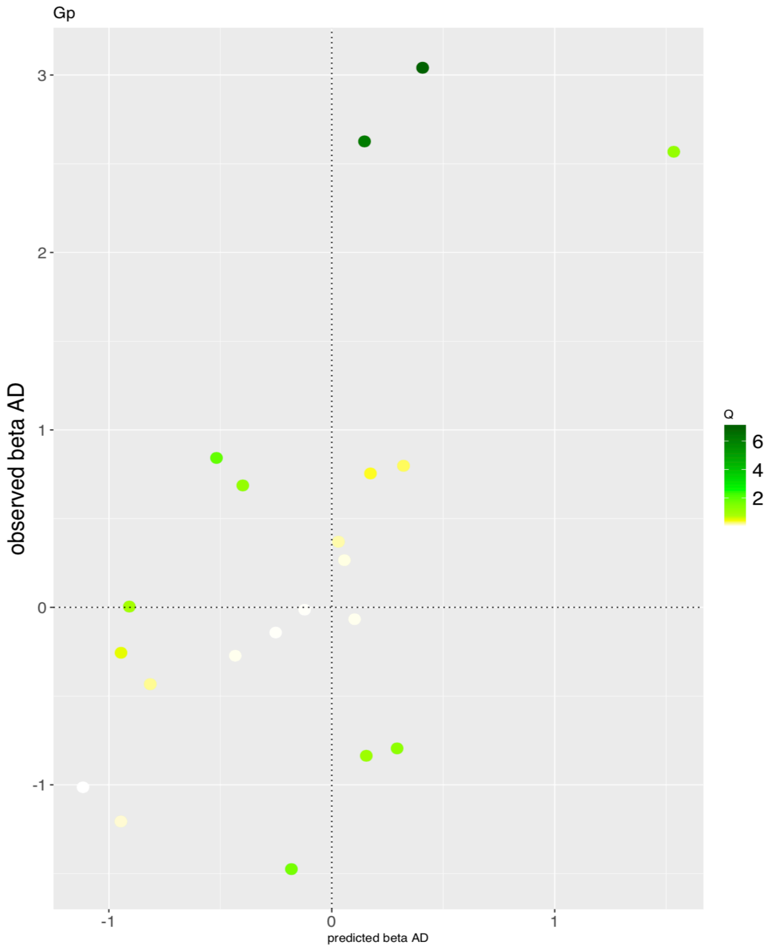

Fig. S7a-S7d. Diagnostic plots for outliers for the top four MR-BMA models.

Predicted associations (x-axis) are plotted against observed associations (y-axis) for AD. Any genetic variant with a Q-statistic>10 is marked with the name of the variant.

Fig. S8a.

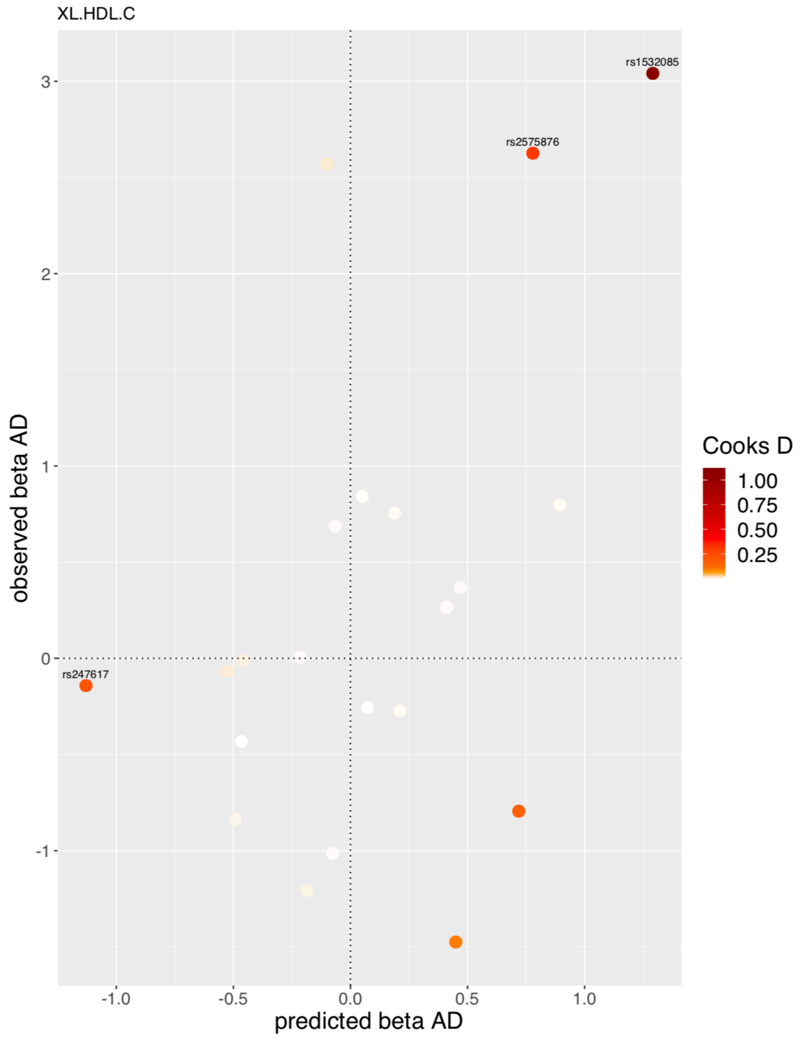

Fig. S8b.

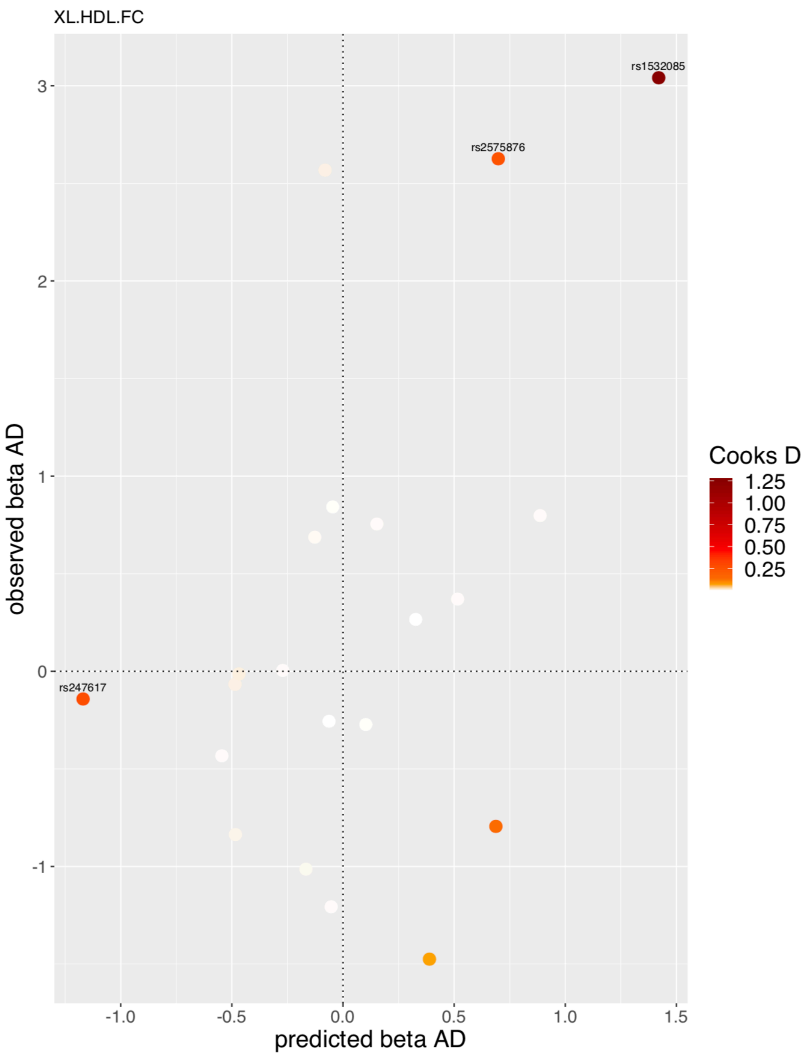

Fig. S8c.

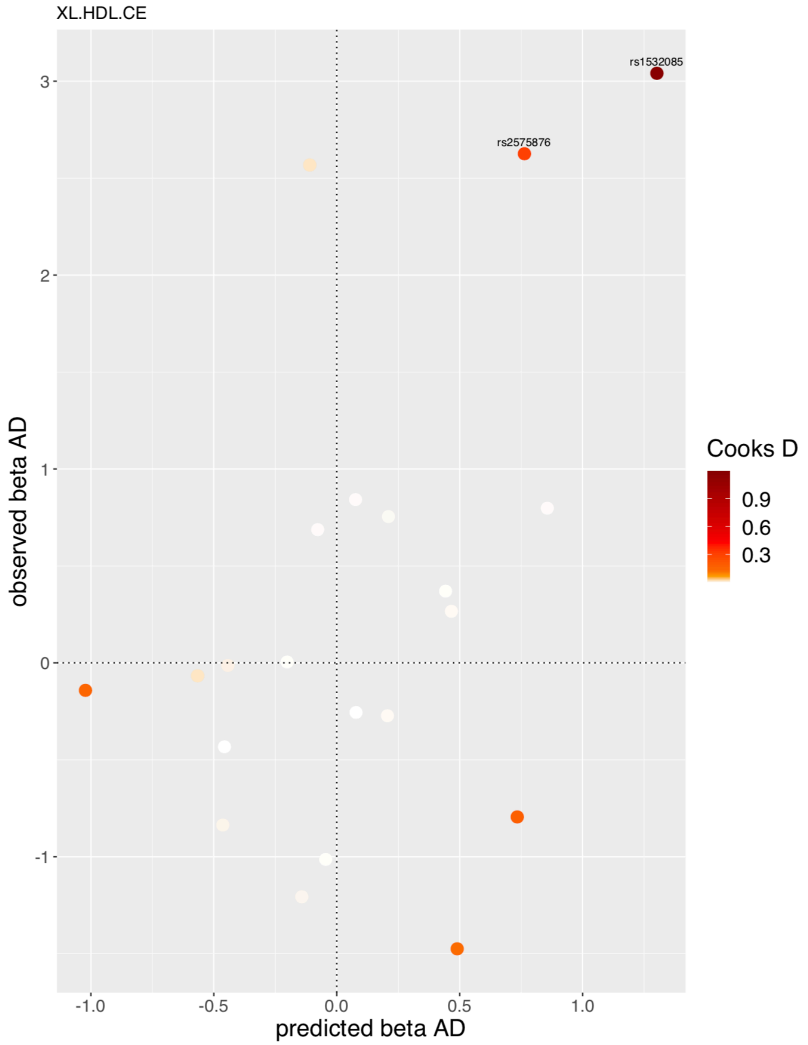

Fig. S8d

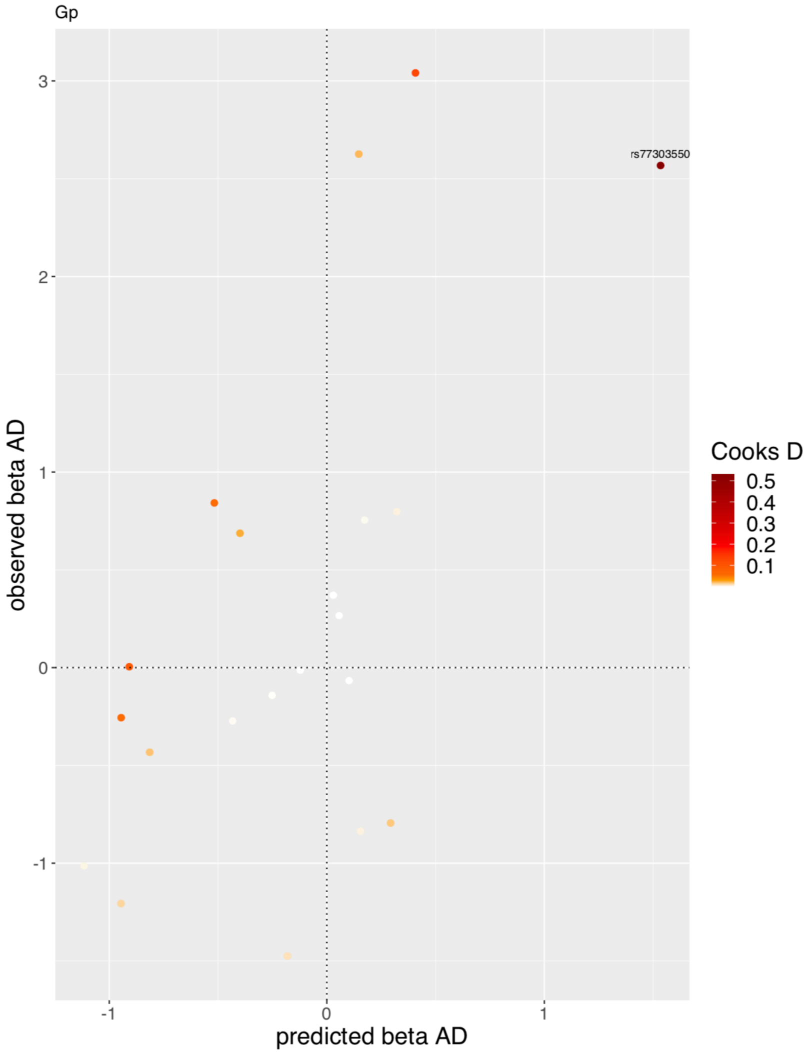

Fig. S8a-S8d. Diagnostic plots for influential genetic variants for the top four MR-BMA models.

Predicted associations (x-axis) are plotted against observed associations (y-axis) for AD. Any genetic variant with a Cook's distance >0.19 (4/21) is marked with the name of the variant.

Fig. S9a. HDLs and influential point: rs1532085 → gene LIPC

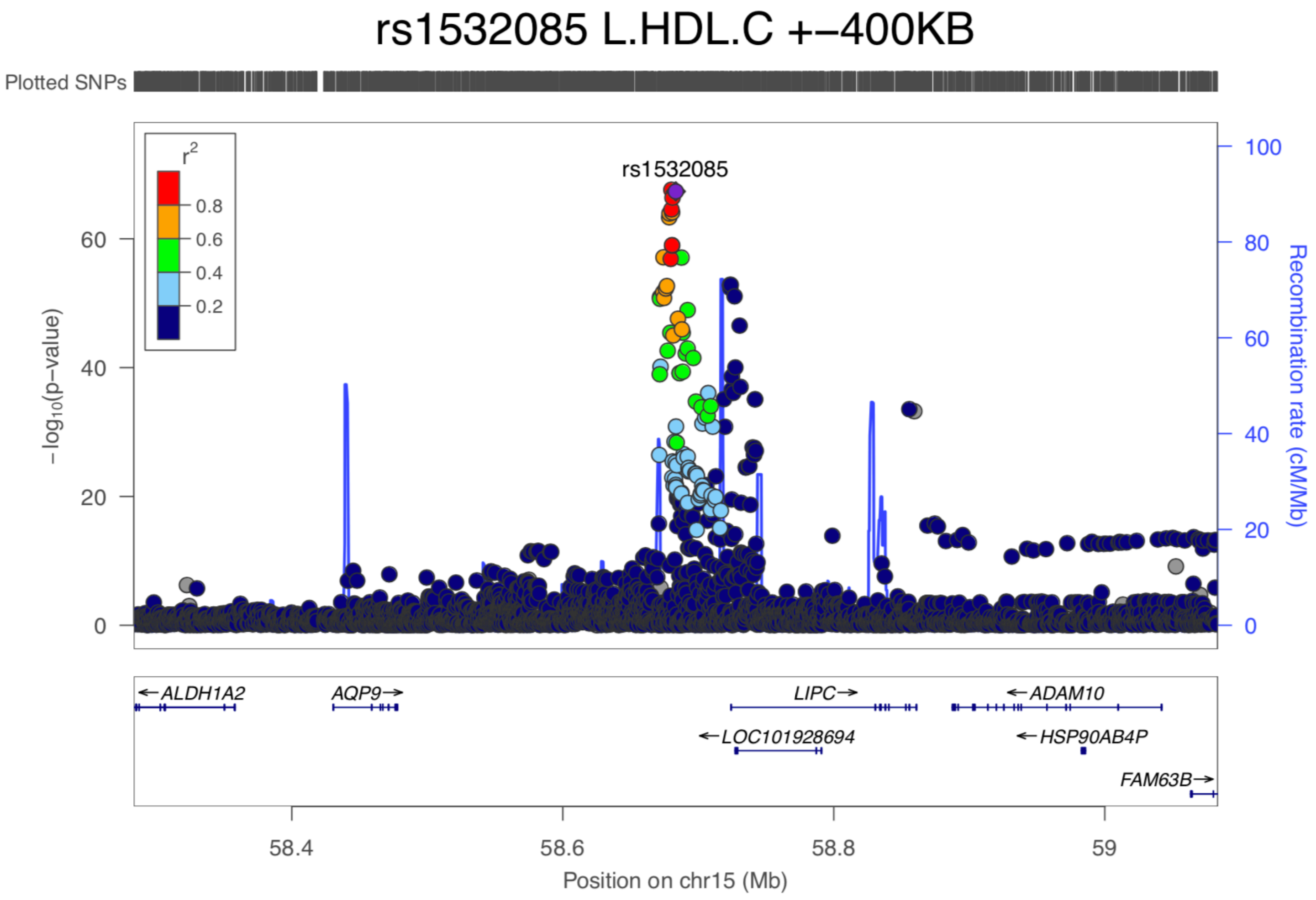

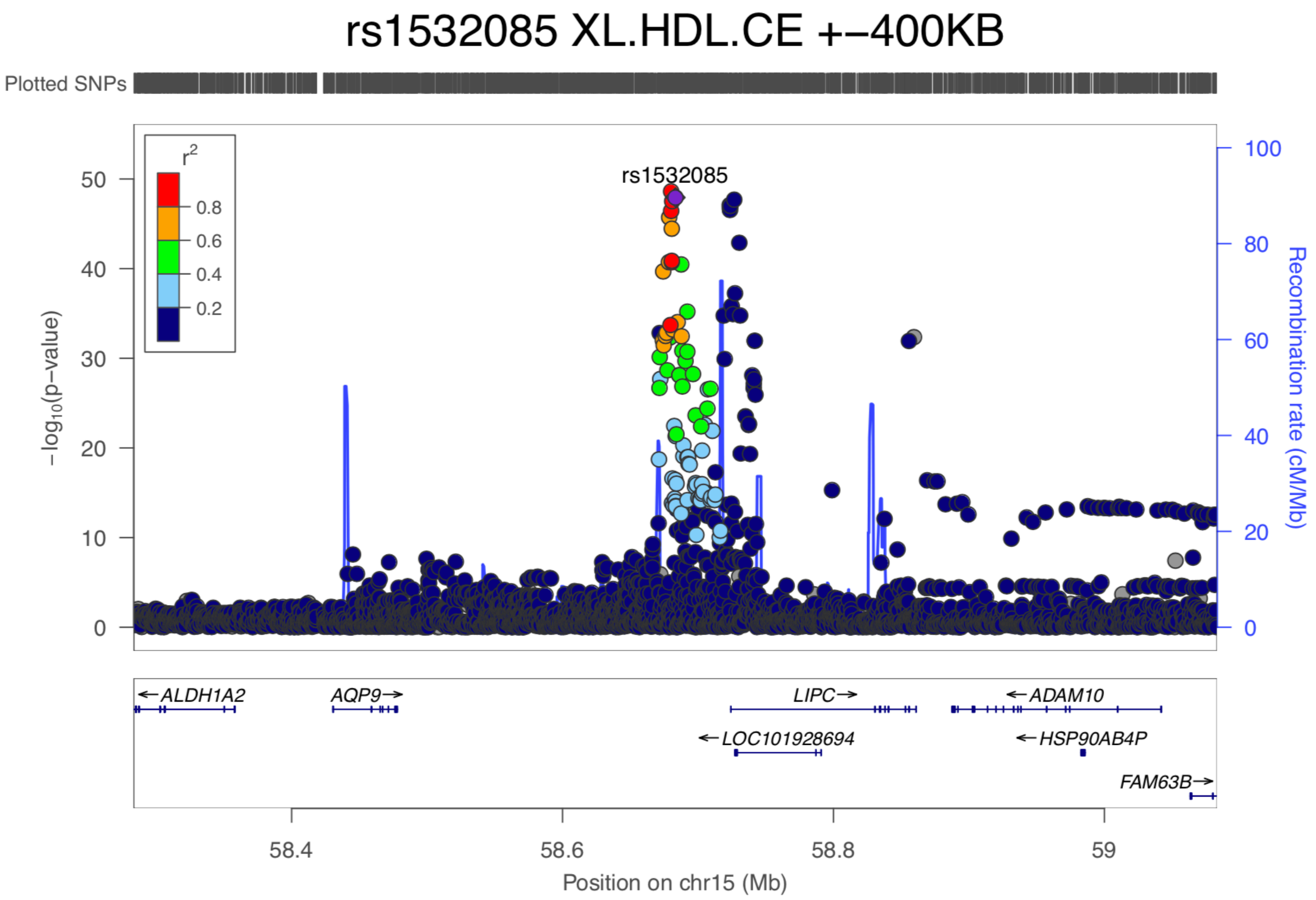
rs1532085 XL.HDL.CE +–400KB
Plotted SNPs
r²
0.8
0.6
0.4
0.2
rs1532085
50
40
30
20
10
0
−log10(p−value)
100
80
60
40
20
0
Recombination rate (cM/Mb)
←ALDH1A2
AQP9→
LIPC→
←ADAM10
←LOC101928694
←HSP90AB4P
FAM63B→
58.4
58.6
58.8
59
Position on chr15 (Mb)

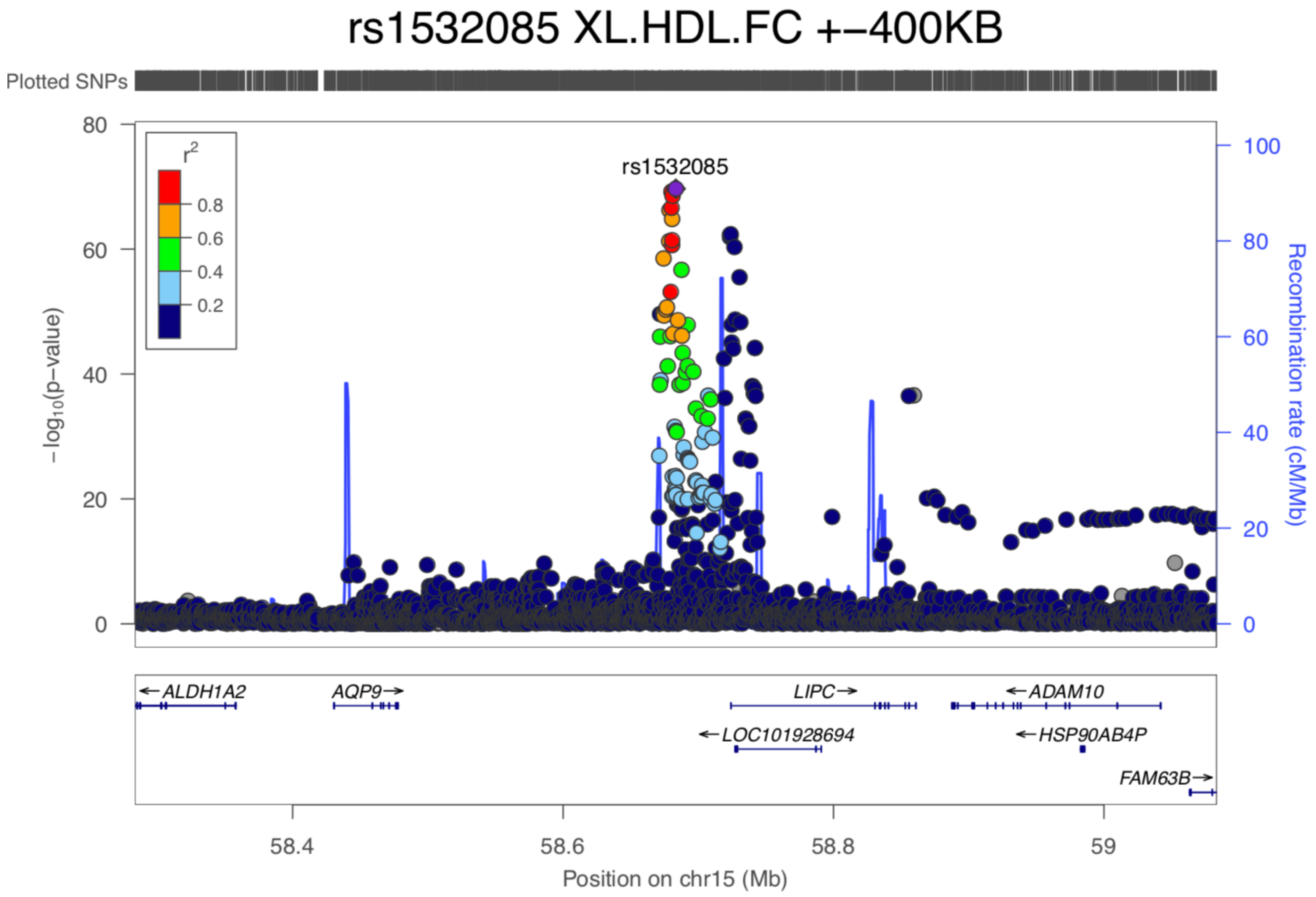
rs1532085 XL.HDL.FC +–400KB
Plotted SNPs
80
60
40
20
0
−log10(p−value)
r2
0.8
0.6
0.4
0.2
rs1532085
100
80
60
40
20
0
Recombination rate (cM/Mb)
←ALDH1A2
AQP9→
LIPC→
←ADAM10
←LOC101928694
←HSP90AB4P
FAM63B→
58.4
58.6
58.8
59
Position on chr15 (Mb)

Fig. S9b. HDLs and influential point: rs2575876 → gene ABCA1

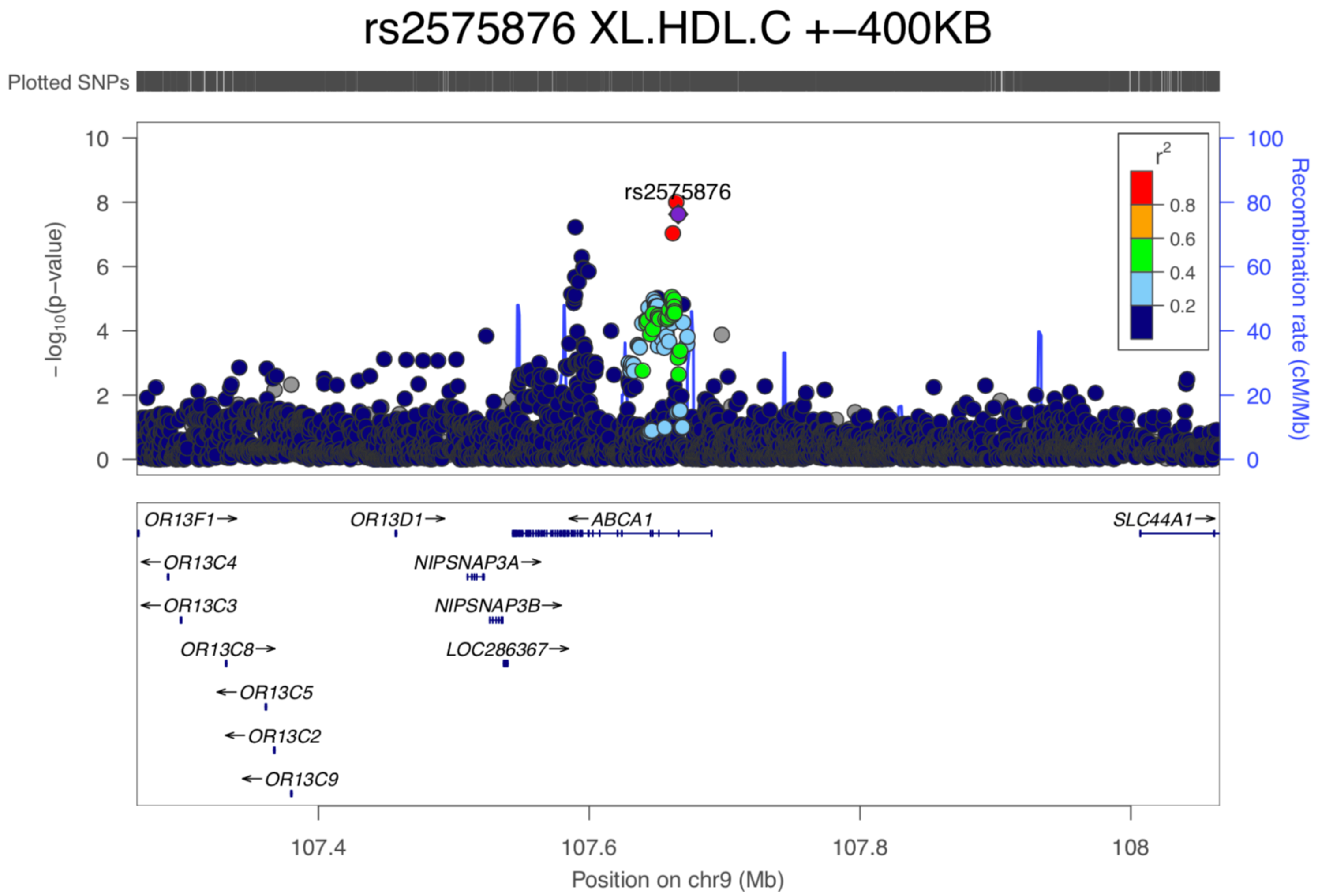

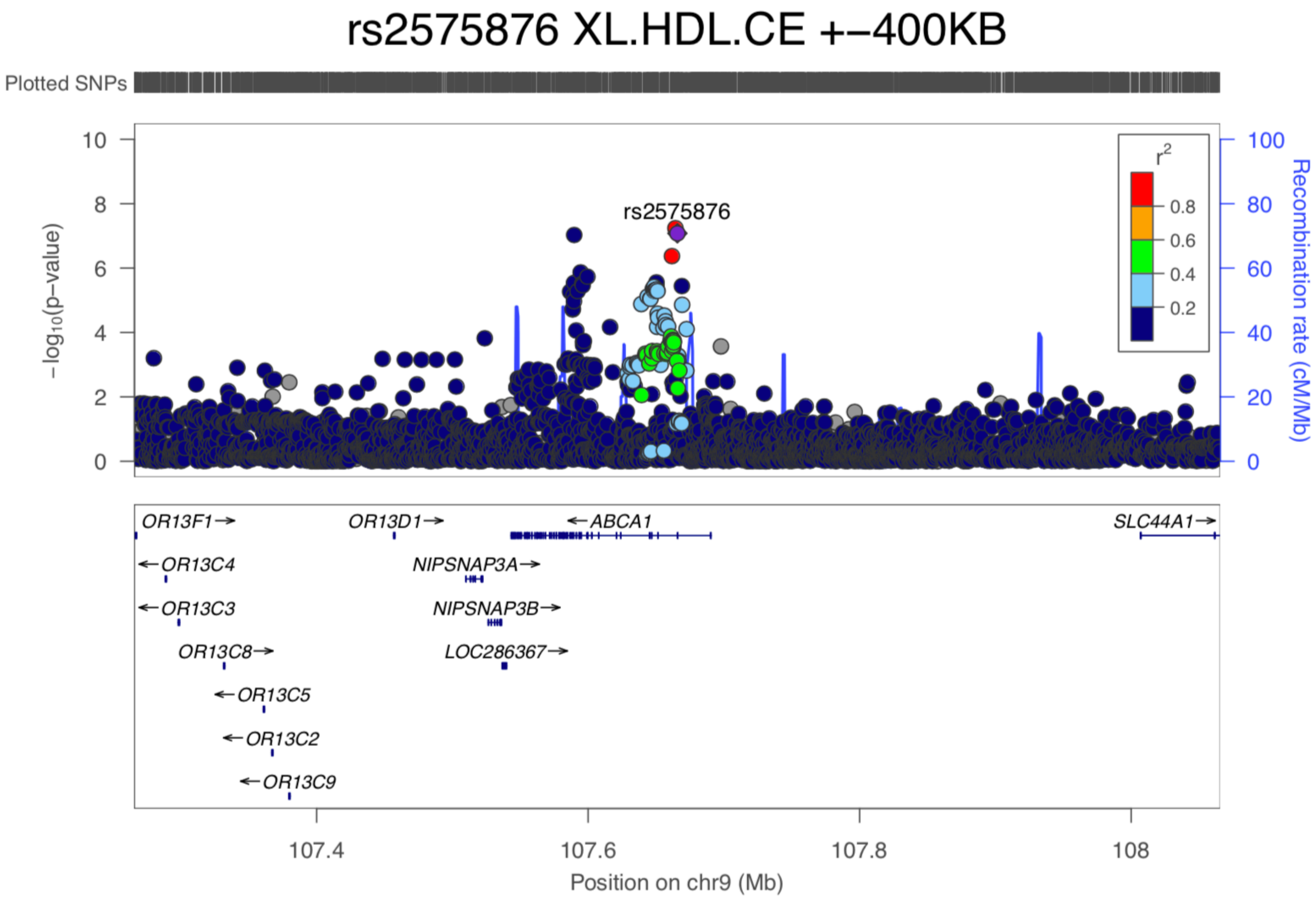

rs2575876 XL.HDL.CE +–400KB
Plotted SNPs
10
8
6
4
2
0
−log10(p−value)
rs2575876
r2
0.8
0.6
0.4
0.2
100
80
60
40
20
0
Recombination rate (cM/Mb)
OR13F1→
OR13D1→
←ABCA1
SLC44A1→
←OR13C4
NIPSNAP3A→
←OR13C3
NIPSNAP3B→
OR13C8→
LOC286367→
←OR13C5
←OR13C2
←OR13C9
107.4
107.6
107.8
108
Position on chr9 (Mb)

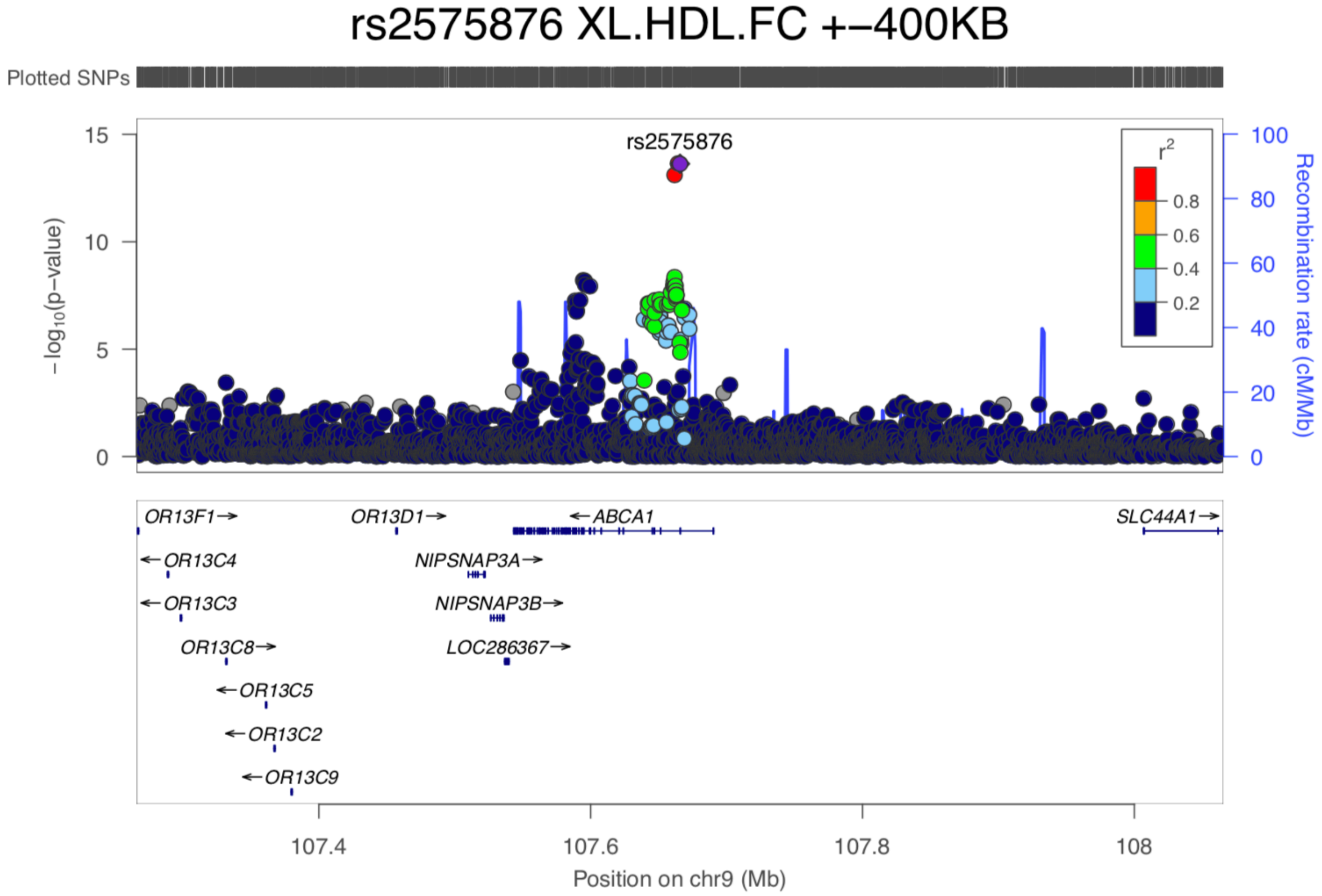
rs2575876 XL.HDL.FC +–400KB
Plotted SNPs
rs2575876
r2
0.8
0.6
0.4
0.2
−log10(p−value)
15
10
5
0
100
80
60
40
20
0
Recombination rate (cM/Mb)
OR13F1→
OR13D1→
←ABCA1
SLC44A1→
←OR13C4
NIPSNAP3A→
←OR13C3
NIPSNAP3B→
OR13C8→
LOC286367→
←OR13C5
←OR13C2
←OR13C9
107.4
107.6
107.8
108
Position on chr9 (Mb)

Fig. S9c. XL.HDL.FC & XL.HDL.C, influential point: rs247617 → gene CETP

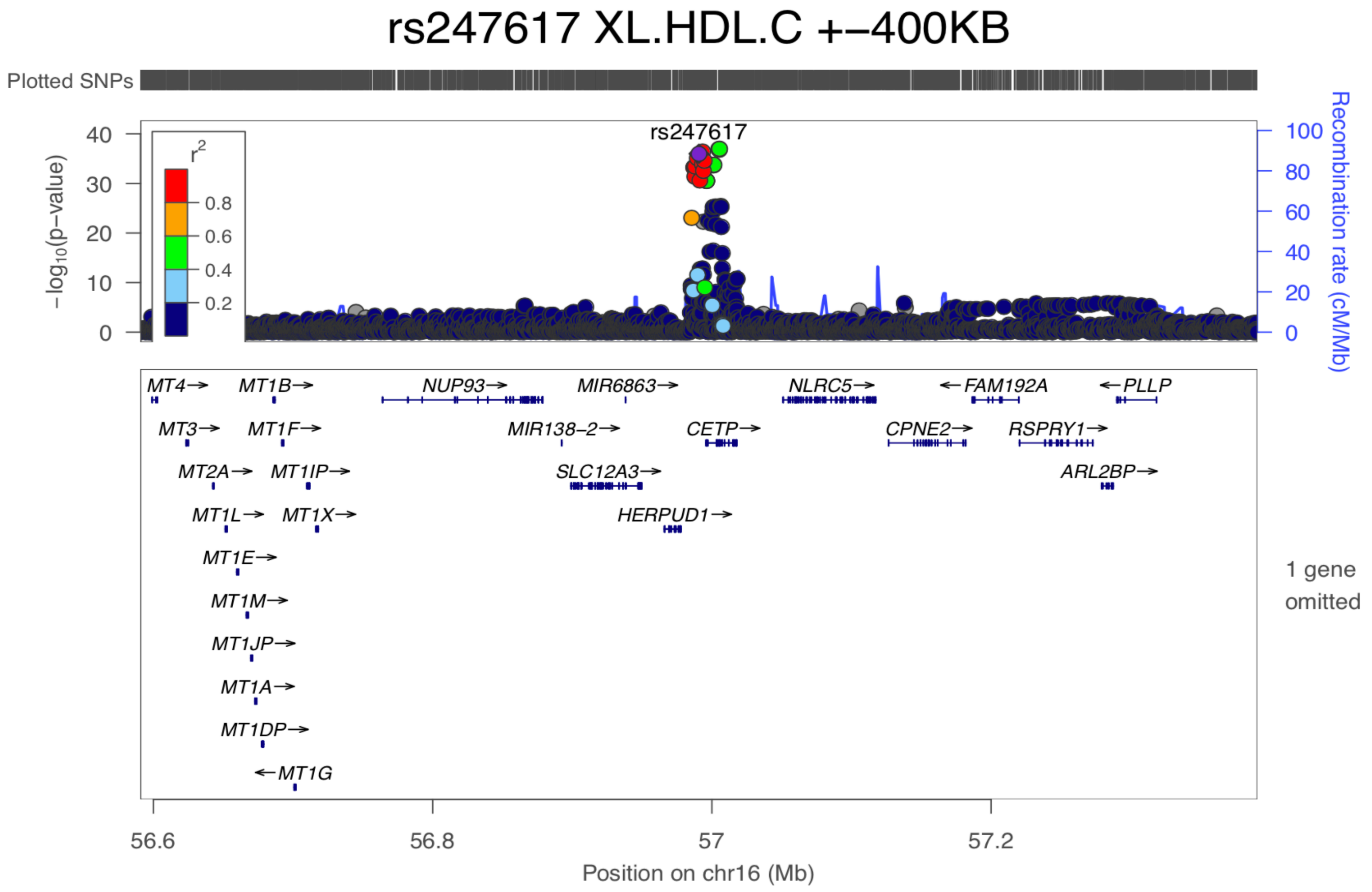

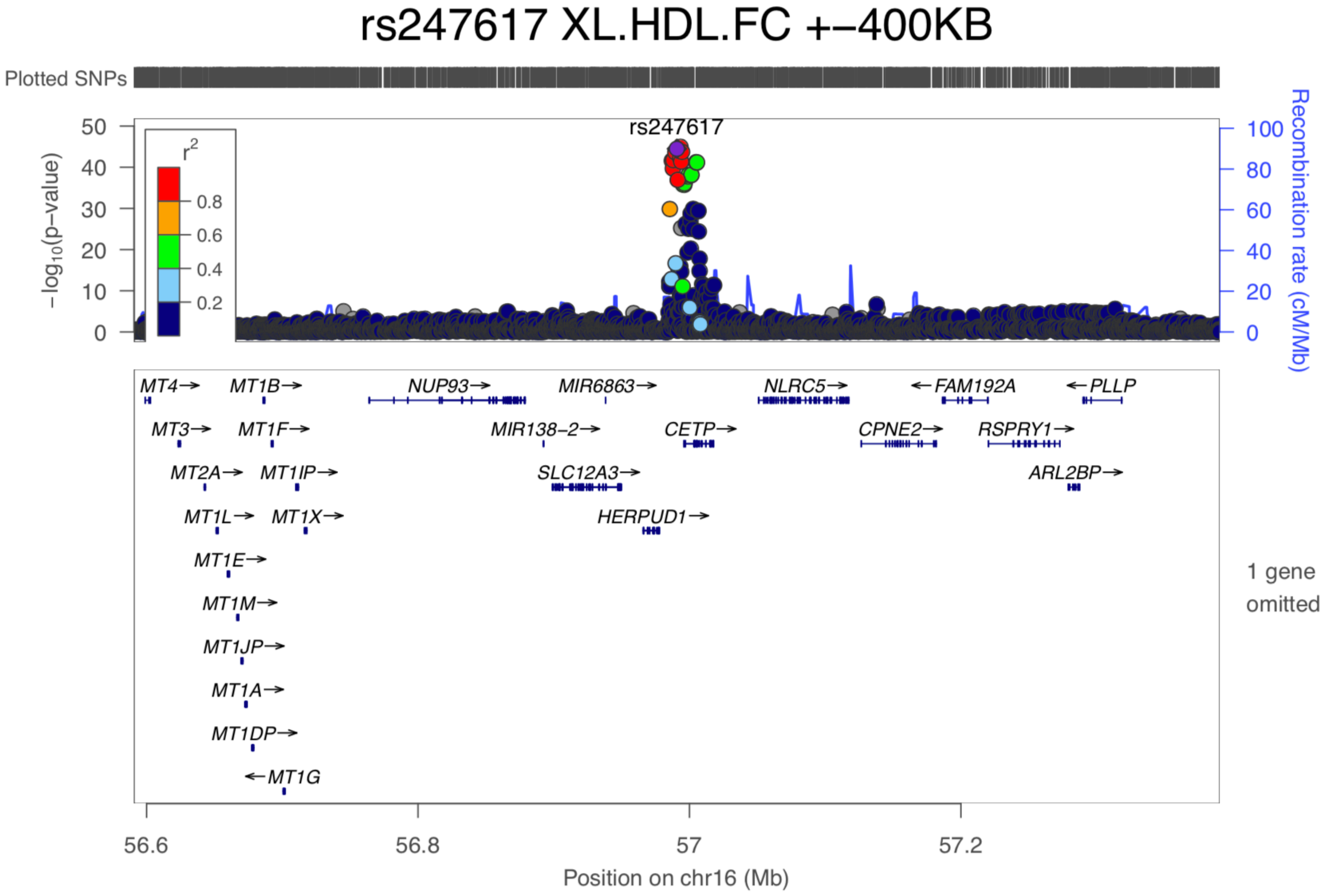
rs247617 XL.HDL.FC +–400KB
Plotted SNPs
rs247617
−log10(p−value)
50
40
30
20
10
0
r2
0.8
0.6
0.4
0.2
100
80
60
40
20
0
Recombination rate (cM/Mb)
MT4→
MT1B→
NUP93→
MIR6863→
NLRC5→
←FAM192A
←PLLP
MT3→
MT1F→
MIR138−2→
CETP→
CPNE2→
RSPRY1→
MT2A→
MT1IP→
SLC12A3→
ARL2BP→
MT1L→
MT1X→
HERPUD1→
MT1E→
MT1M→
MT1JP→
MT1A→
MT1DP→
←MT1G
1 gene omitted
56.6
56.8
57
57.2
Position on chr16 (Mb)

Fig. S9d. Glycoprotein and influential point: rs77303550 → gene TXNL4B

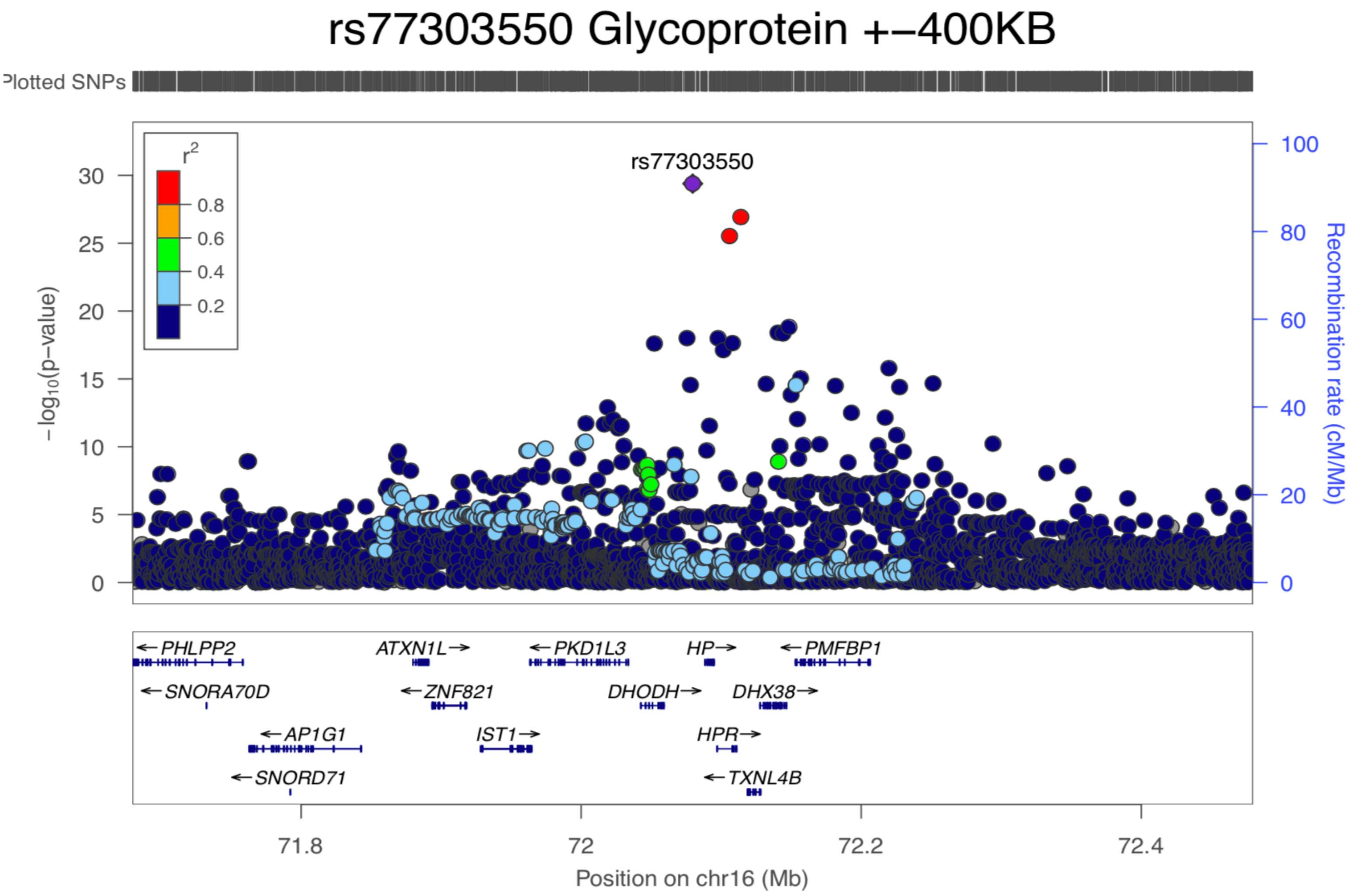

Fig. S9e. Kunkle lead SNP: rs593742 → gene ADAM10

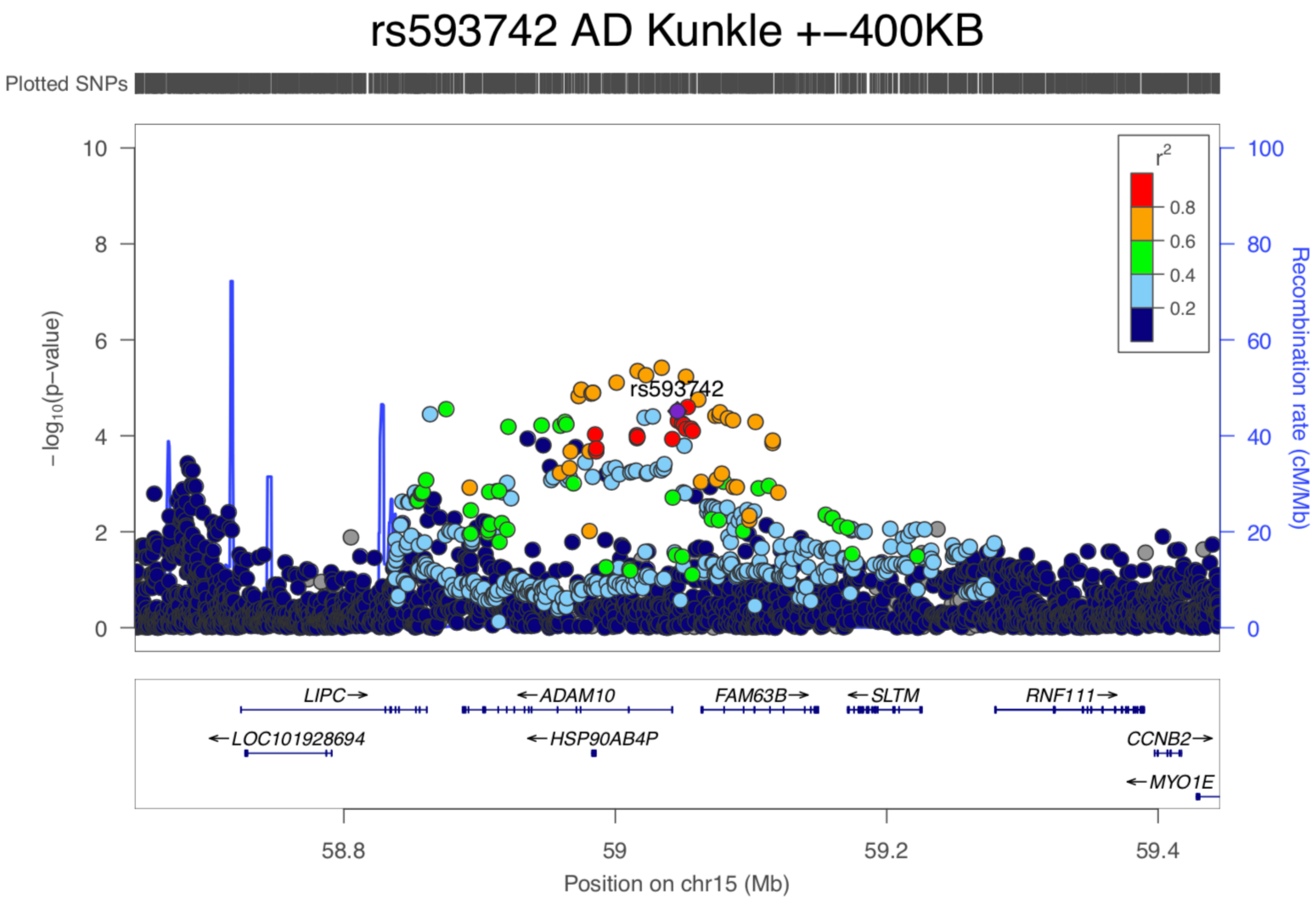

Fig. S9a-S9e. Locus zoom plots confirming LD regions for influential points in univariable and BMA-MR analyses.

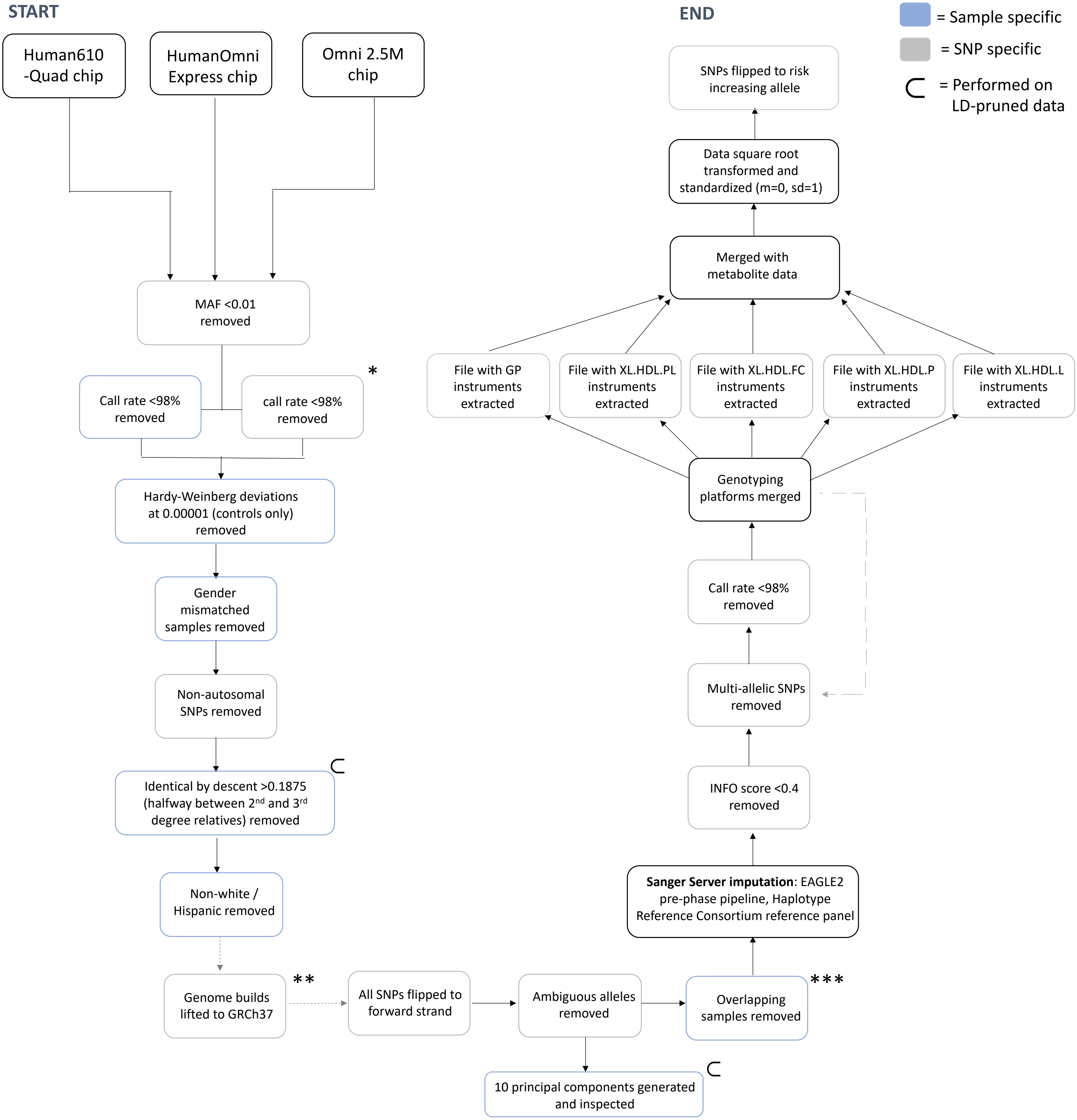

** Missingness for both SNPs and samples were inspected iteratively, from 90-98%, in steps of 1%.*
*** Required for Human610-Quad platform only.*
**** For overlaps between Human610-Quad and Omni 2.5M , duplicates in Omni 2.5M removed and Human610-Quad retained. For overlaps between HumanOmniExpress and Omni 2.5M, duplicates in HumanOmniExpress removed and Omni 2.5M retained. No overlaps observed between Human610-Quad and HumanOmniExpress.*

Fig. S10. Flow chart illustrating quality control pipeline for ADNI individual level genotype data used in post-hoc 2-stage least squares MR.

SI References

1. Bulik-Sullivan, B. *et al*. (2015). An atlas of genetic correlations across human diseases and traits. *Nature genetics*, *47*(11), 1236.

2. Burgess, S, *et al*. (2019). Guidelines for performing Mendelian randomization investigations. *Wellcome Open Research*, *4*.

3. Proitsi P*, et al.* (2018) Lifetime cognition and late midlife blood metabolites: findings from a British birth cohort. *Translational psychiatry* 8(1):203.

4. Richards M*, et al.* (2014) Lifetime affect and midlife cognitive function: prospective birth cohort study. *Br J Psychiatry* 204(3):194-199.

5. Wallace, C. (2020). Eliciting priors and relaxing the single causal variant assumption in colocalisation analyses. *PLoS Genetics, 16*(4), e1008720.

6. Kettunen J*, et al.* (2016) Genome-wide study for circulating metabolites identifies 62 loci and reveals novel systemic effects of LPA. *Nat Commun* 7:11122.

7. Kunkle BW*, et al.* (2019) Genetic meta-analysis of diagnosed Alzheimer's disease identifies new risk loci and implicates Abeta, tau, immunity and lipid processing. *Nature genetics* 51(3):414-430.

8. Weiner MW, *et al.* (2010). The Alzheimer's disease neuroimaging initiative: progress report and future plans. *Alzheimer's & Dementia,* 6(3): 202-211
